# Psychological Safety Competency Training During the Clinical Internship From the Perspective of Health Care Trainee Mentors in 11 Pan-European Countries: Mixed Methods Observational Study

**DOI:** 10.2196/64125

**Published:** 2024-10-07

**Authors:** Irene Carrillo, Ivana Skoumalová, Ireen Bruus, Victoria Klemm, Sofia Guerra-Paiva, Bojana Knežević, Augustina Jankauskiene, Dragana Jocic, Susanna Tella, Sandra C Buttigieg, Einav Srulovici, Andrea Madarasová Gecková, Kaja Põlluste, Reinhard Strametz, Paulo Sousa, Marina Odalovic, José Joaquín Mira

**Affiliations:** 1 Department of Health Psychology Miguel Hernández University of Elche Elche Spain; 2 Department of Health Psychology and Research Methodology Faculty of Medicine Pavol Jozef Šafárik University Kosice Slovakia; 3 Tartu Health Care College Tartu Estonia; 4 Wiesbaden Institute for Healthcare Economics and Patient Safety (WiHelP) Wiesbaden Business School RheinMain University of Applied Sciences Wiesbaden Germany; 5 Public Health Research Centre National School of Public Health NOVA University Lisbon Lisbon Portugal; 6 Comprehensive Health Research Center National School of Public Health NOVA University Lisbon Lisbon Portugal; 7 University Hospital Centre Zagreb University of Zagreb Zagreb Croatia; 8 Pediatric Center, Institute of Clinical Medicine Faculty of Medicine Vilnius University Vilnius Lithuania; 9 BENU Pharmacy PHOENIX Group Serbia Belgrade Serbia; 10 Faculty of Social and Health Care LAB University of Applied Sciences Lappeenranta Finland; 11 Department of Health Systems Management and Leadership Faculty of Health Sciences University of Malta Malta Malta; 12 Cheryl Spencer Department of Nursing University of Haifa Haifa Israel; 13 Institute of Applied Psychology Faculty of Social and Economic Sciences Comenius University Bratislava Bratislava Slovakia; 14 Institute of Clinical Medicine University of Tartu Tartu Estonia; 15 Faculty of Pharmacy University of Belgrade Belgrade Serbia; 16 Foundation for the Promotion of Health and Biomedical Research of the Valencia Region (FISABIO) Sant Joan d'Alacant Spain

**Keywords:** psychological safety, speaking up, professional competence, patient safety, education, adverse event

## Abstract

**Background:**

In the field of research, psychological safety has been widely recognized as a contributing factor to improving the quality of care and patient safety. However, its consideration in the curricula and traineeship pathways of residents and health care students is scarce.

**Objective:**

This study aims to determine the extent to which health care trainees acquire psychological safety competencies during their internships in clinical settings and identify what measures can be taken to promote their learning.

**Methods:**

A mixed methods observational study based on a consensus conference and an open-ended survey among a sample of health care trainee mentors from health care institutions in a pan-European context was conducted. First, we administered an ad hoc questionnaire to assess the perceived degree of acquisition or implementation and significance of competencies (knowledge, attitudes, and skills) and institutional interventions in psychological safety. Second, we asked mentors to propose measures to foster among trainees those competencies that, in the first phase of the study, obtained an average acquisition score of <3.4 (scale of 1-5). A content analysis of the information collected was carried out, and the spontaneity of each category and theme was determined.

**Results:**

In total, 173 mentors from 11 pan-European countries completed the first questionnaire (response rate: 173/256, 67.6%), of which 63 (36.4%) participated in the second consultation. The competencies with the lowest acquisition level were related to warning a professional that their behavior posed a risk to the patient, managing their possible bad reaction, and offering support to a colleague who becomes a second victim. The mentors’ proposals for improvement of this competency gap referred to training in communication skills and patient safety, safety culture, work climate, individual attitudes, a reference person for trainees, formal incorporation into the curricula of health care degrees and specialization pathways, specific systems and mechanisms to give trainees a voice, institutional risk management, regulations, guidelines and standards, supervision, and resources to support trainees. In terms of teaching methodology, the mentors recommended innovative strategies, many of them based on technological tools or solutions, including videos, seminars, lectures, workshops, simulation learning or role-playing with or without professional actors, case studies, videos with practical demonstrations or model situations, panel discussions, clinical sessions for joint analysis of patient safety incidents, and debriefings to set and discuss lessons learned.

**Conclusions:**

This study sought to promote psychological safety competencies as a formal part of the training of future health care professionals, facilitating the translation of international guidelines into practice and clinical settings in the pan-European context.

## Introduction

### Theoretical Background

#### Overview

Clinical errors are a common occurrence in health care, and many of them are preventable [1,2]. Adverse events involving health care professionals, including medical students during their internships, are unfortunately widespread [3]. These events have negative consequences for patients, health care professionals, and the health care system, commonly referred to as the first, second, and third victims, respectively [4-6]. Hence, prioritizing error prevention is crucial at both the local and national health care system levels. This focus serves to mitigate harm, reduce costs, and restore trust in health care [7].

While patient safety management in health care institutions was originally based on a reactive approach focused on acting after an incident and detecting what failed behind it, over time, this way of pursuing safety has evolved to a more positive one in which the objective is no longer just avoiding something going wrong (Safety I) but, above all, ensuring that everything goes right (Safety II). Far from being antagonistic, these 2 perspectives complement each other and allow for a more balanced and flexible approach to the reality of health care [8]. Thus, safety is linked not only to concepts such as incidents, errors, failures, or liability but also to other concepts, such as occupational well-being, resilience, or psychological safety.

#### Second Victims

Improved patient safety, in turn, has a positive impact on patient outcomes [9,10] and helps prevent health care professional burnout [11]. Health care professionals and even medical students involved in adverse events often experience symptoms akin to those of second victims, with profound implications for their emotional well-being as well as their professional and personal lives [9,12,13]. Recently, Vanhaecht et al [14], based on the literature and the expert consensus, defined a second victim as follows: “Any health care worker, directly or indirectly involved in an unanticipated adverse patient event, unintentional healthcare error, or patient injury and who becomes victimized in the sense that they are also negatively impacted.”

#### Patient Safety Culture

Despite the recognition of the need for safer patient care, there remain various barriers [15-17]. Moreover, the effective processes after the adverse event, such as reporting of an adverse event, the analysis of the event, and open disclosure, can be hindered. These effective practices stem from and contribute to the psychological safety climate within the organization, encompassing elements such as a blame-free culture and a just culture [18,19].

#### Psychological Safety

Psychological safety is an important part of the safety culture in health care organizations. It is defined as a belief that individuals can feel safe to disagree, ask questions, and report mistakes without negative consequences and that they can cooperate as a team with mutual respect and trust [20]. Psychological safety contributes to improved outcomes in clinical training (eg, willingness to report adverse events [21] and speak up [22]) and, therefore, may be associated with better patient outcomes and improved safety culture [23]. Thus, comprehensive patient safety management combining Safety I and Safety II approaches requires psychologically safe clinical environments [8].

#### Speaking Up for Ensuring Patient Safety

Psychological safety is pivotal in transitioning from a proactive to a generative patient safety culture [24]. It enables health care professionals to speak up without fear of consequences, which is crucial for overcoming barriers to safe care practices, including effective adverse event reporting and analysis [25]. Institutions must also prioritize the support and consideration of second victims as failing to do so can compromise patient safety [24].

Creating safe learning environments is essential for fostering psychological safety among future health care professionals. Training programs emphasizing open communication, mutual respect, and psychological safety equip students and professionals with the skills necessary for a safer health care system. By prioritizing these elements, health care organizations can establish a culture that supports both patient and provider well-being, ultimately enhancing safety and quality of care [24,25].

In summary, integrating psychological safety into health care practices creates a comprehensive safety framework, emphasizing a supportive culture, proactive error management, and the development of resilient health care professionals. This approach not only addresses immediate safety concerns but also contributes to the long-term sustainability of health care systems by nurturing a safety culture and psychological well-being [24].

### Current Gap

Poor attention has been paid to promoting psychological safety in health care and medical education [26,27]. Future health care professionals’ perceptions, attitudes, skills, and knowledge regarding safety culture cocreate a safe health care environment and contribute to better patient safety as well as a psychologically safer climate [28,29]. Strategies to establish psychological safety in clinical supervision were introduced to improve future health care professionals’ training [30]. Little is known about how health care professionals are trained in psychological safety in European countries, which competencies and skills promoting psychological safety they obtain during their clinical internship, and which of them are considered important from the perspective of health care professionals in practice.

### Methodological Framework

In the health care field, competence is defined as *“*the habitual and judicious use of communication, knowledge, technical skills, clinical reasoning, emotions, values, and reflection in daily practice to benefit both individuals and the community*”* [31]. This study’s methodology aligns with the taxonomy by Bloom et al [32], which identifies 3 domains of competencies: cognitive (knowledge), affective (attitudes), and psychomotor (skills), collectively referred to as knowledge, skills, and attitudes or knowledge, attitudes, and skills (KAS) [33].

**Figure 1:**
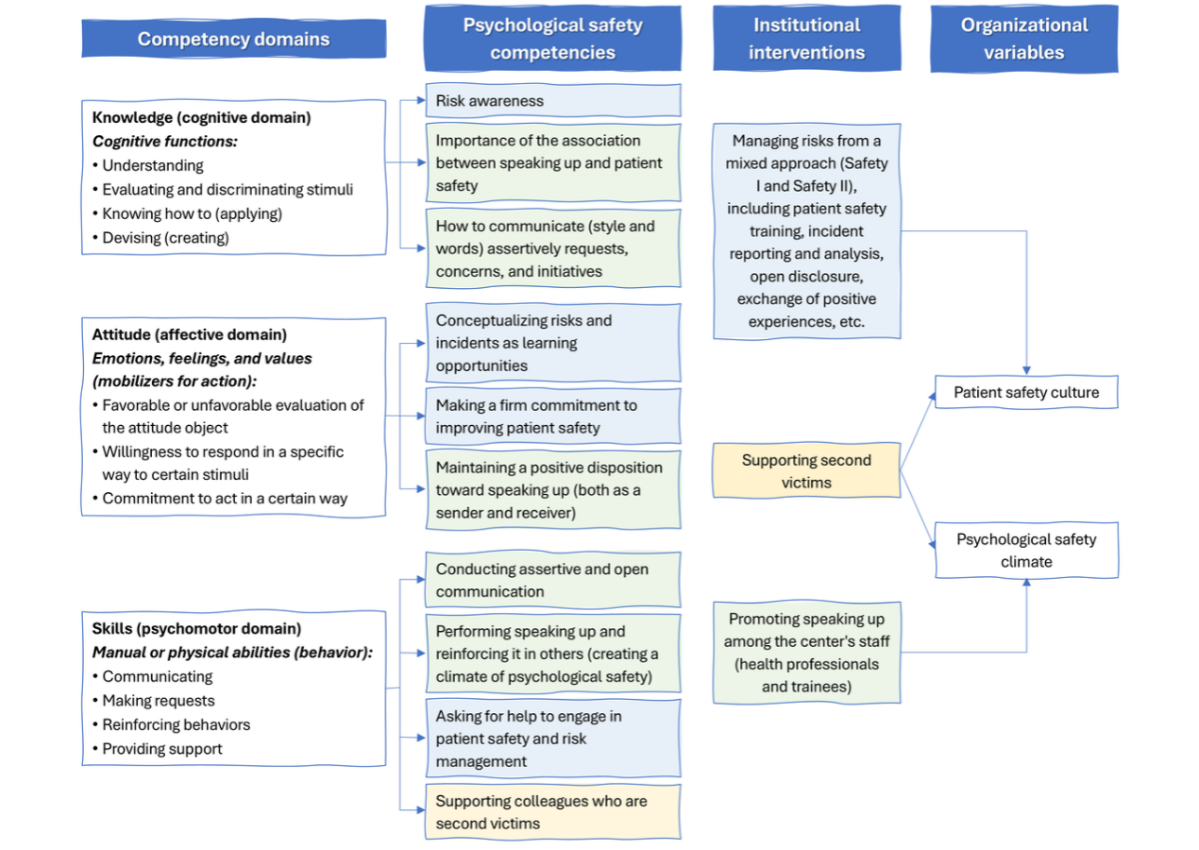
Integrated proposal of the study’s theoretical background on psychological safety and the methodological framework based on the taxonomy of competencies by Bloom et al [32] and the knowledge, skills, and attitudes model.

Figure 1 [32] shows the integration of this study’s theoretical background and methodological framework. To assess the professional competence of trainees in psychological safety, they must demonstrate that they know (knowledge), know how to be (attitudes), and know how to do (skills) regarding the topics related to psychological safety described in the theoretical framework. To our knowledge, none of the instruments or frameworks explicitly define competencies in psychological safety. Consequently, this study proposed their measurement based on integrating the KAS model with the variables considered in the instruments available to evaluate speaking up, psychological safety, and support for second victims. Moreover, this approach finds that the culture and climate of the organization play a crucial role in implementing psychological safety competencies. For this reason, we proposed evaluating institutional interventions to promote psychological safety in clinical settings in parallel to assessing these competencies.

### Objectives and Hypotheses

The aim of this study was to determine the extent to which health care trainees acquire psychological safety competencies during their internships in clinical settings and identify what measures can be taken to promote their learning.

Our previous hypothesis was that mentors perceive that the level of training and acquisition of psychological safety competencies (as defined in our framework) among European trainees during their internships in clinical settings is low. This hypothesis is supported by the absence of a previous framework that explicitly conceptualizes psychological safety competencies, the relatively low frequency with which trainees speak up on patient safety [34,35], and the generalized lack of formal content on patient safety in the curricula of health care studies in Europe [36]. Regarding measures to promote the learning of these competencies, we expect mentors to identify some of the barriers that currently limit their acquisition by trainees, among which are organizational variables. Therefore, their suggestions will incorporate not only specific initiatives of an educational nature but also others oriented toward cultural forms and patterns of institutional behavior that promote a climate of psychological safety in clinical settings.

## Methods

### Study Design

We combined quantitative and qualitative research methods and approaches to understand the wideness and depth of the problem of psychological safety training in health care research with mentors of residents and students in health care disciplines from 11 pan-European countries. This study was conducted in 2 phases from February 2022 to July 2023. First, we conducted a web-based consensus conference using a structured questionnaire to prioritize psychological safety competencies and institutional interventions to foster their acquisition among trainees. Subsequently, we performed a second consultation based on an open-ended survey to explore recommended actions to enhance the development of those psychological safety competencies that, according to the results of the consensus conference, were considered significant but the training on them was still lacking or incomplete during internships.

This manuscript was developed in accordance with the Checklist for Reporting Results of Internet E-Surveys [37] (phase 1, survey study) and the COREQ (Consolidated Criteria for Reporting Qualitative Research) [38] (phase 2, qualitative study).

### Ethical Considerations

We followed national regulations to obtain ethics approvals in Estonia (Research Ethics Committee of the University of Tartu, approval 364/T-11; May 16, 2022), Israel (Ethics Committee of the Faculty of Social Welfare and Health Sciences, University of Haifa, approval 036/22; December 21, 2021), Slovakia (Ethics Committee of Pavol Jozef Šafárik University in Košice, approval 11N/2022; March 28, 2022), and Spain (Research Ethics Committee of the San Juan de Alicante University Hospital, approval 22/012; February 23, 2022). In other countries, previous ethics approvals were also considered valid.

Before the participants were registered on the web platform, informed consent was obtained. Likewise, the questionnaire only allowed access to the questions after the participants had explicitly confirmed consent to participate in the study. Participants were given a contact email to exercise their right to withdraw from participation at any time during the study. To allow traceability of the data in the different phases of the study, but at the same time, to guarantee the confidentiality of the participants and their responses, these were deidentified. No form of financial compensation was provided for participation or recruitment.

### Recruitment

We invited 256 health care trainee mentors from 11 pan-European countries (20-25 mentors per country), including Croatia, Estonia, Finland, Germany, Israel, Lithuania, Malta, Portugal, Serbia, Slovakia, and Spain, to participate in this study. The invitation to these countries allowed for the collection of information representative of educational models from Northern, Eastern, Southern, and Central Europe. We enrolled professionals assigned to health care institutions (inpatient or outpatient, community, and social care settings) associated with universities or other formal training institutions who were responsible for mentoring and supervising residents and students during their clinical internships in the following areas: family medicine, obstetrics, midwifery, pharmacy, surgery, and other medical fields such as pediatrics. These specialties were chosen based on the main rotation areas of the training and residency programs. Academic-only mentors were excluded. Participants were recruited through convenience sampling by 1 or 2 members of the European Researchers’ Network Working on Second Victims (ERNST) Consortium (European Cooperation in Science and Technology Action 19113) [39] from each participating country, who acted as local coordinators. The ERNST includes 28 European countries, integrating experienced research teams focused on patient safety issues, with most working in clinical and academic settings. This network was the vehicle for coordinating the study but not an inclusion criterion for recruiting participants. Thus, all national coordinators were network members, but not all participants involved in the study belonged to ERNST. Only the countries in this European consortium (11/28, 39%) that voluntarily decided to join the study were involved.

In each participating country, trainee mentors were contacted by the national study coordinator through an invitation letter providing information about the study and its objective, content, and procedure. Those participants who agreed to be involved in the study voluntarily completed the questionnaire or survey used for data collection depending on the study phase.

### Phase 1: Degree of Acquisition or Implementation and Significance of Psychological Safety Competencies and Institutional Interventions (Consensus Conference)

#### Procedure

For the first phase of the study, consisting of a web-based consensus conference, we developed an ad hoc questionnaire whose content was structured into 4 blocks of items, the first 3 describing KAS related to psychological safety competencies and the last one describing institutional actions to promote the acquisition of these competencies among trainees. For the development of the questionnaire items, we relied on the validated instrument by Richard et al [40] on psychological safety and speaking up behavior and on those by Lee et al [41] and Schnall et al [42], which propose the assessment of patient safety competencies based on the measurement of KAS that make up each core competency.

The national coordinators from Croatia, Estonia, Finland, Germany, Israel, Portugal, Serbia, Slovakia, and Spain were responsible for the translation and back translation process of the English questionnaire into the language of their country (Multimedia Appendix 1). Participants from Malta and Lithuania completed the English questionnaire.

Before the consensus conference, the questionnaire’s readability and face validity were tested between February 2022 and March 2022. In total, 3 mentors per country completed the questionnaire. Given the readability test results, several changes to the questionnaire were made, including rewording some items, reducing the response scale levels, deleting 1 original item, and adding 2 new items for clarity. The overall evaluation of the questionnaire (scale of 0-5) was positive in terms of satisfaction (mean 4.0, SD 0.9), usefulness of the information (mean 4.6, SD 0.7), and usefulness for curricular improvement (mean 4.4, SD 0.6) and somewhat less favorable in terms of length (mean 3.6, SD 0.7).

The final version of the questionnaire consisted of 29 items grouped into the 4 original blocks (Textbox 1). Mentors were asked to assess the degree of acquisition among the trainees of their institution of knowledge (7 items), attitudes (6 items), and skills (7 items) in psychological safety and their degree of significance from their point of view using a 5-point Likert response scale (1=no acquisition at all or not important at all; 5=fully acquired or very important). For institutional interventions (9 items), participants were asked to determine the degree of implementation and significance using a 5-point Likert response scale (1=not yet implemented or not important at all; 5=fully implemented or very important). In the questionnaire instructions, we provided respondents with the definition of the psychological safety concept. We also added a brief conceptual clarification in those items that referred to other secondary terms (such as *second victim*). The questionnaire was distributed via email and using a web-based platform owned by the research group for conducting opinion studies (password-protected survey) between October 2022 and December 2022. A total of 3 reminders were sent during this period to ensure an adequate response rate.

**Final version of the questionnaire to evaluate the degree of acquisition or implementation and significance of psychological safety competencies and institutional actions based on the knowledge, skills, and attitudes framework (phase 1).**

**Knowledge—in my opinion, internships in my work environment provide trainees with the competency to:**

- Understand that an open and direct expression of concerns about patient safety can prevent the occurrence of incidents that could cause harm to the patient.
- Know how to communicate assertively a concern about patient safety to another health care professional (of the same level or higher; eg, what words to choose, how to start and finish the conversation, and what tone of voice or gestures to use).
- Distinguish between situations that could cause avoidable harm to the patient and those that do not represent a high risk for patient safety.
- Choose the best moment to communicate specific concerns about patient safety to another health care professional (of the same level or higher).
- Know how to assertively warn another health care professional (of the same level or higher) of the risk of ignoring an important patient safety rule (eg, words to choose, how to start and finish the conversation, and what tone of voice or gestures to use).
- Know how to deal constructively with the possible negative reaction of a health care professional (of the same level or higher) after having warned them that they were overlooking an important rule for patient safety.
- Know how to express specific proposals that could improve the patient safety in the unit.

**Attitude—in my opinion, internships in my work environment provide trainees with the competency to:**

- Commit to the identification and prevention of risks for patient safety.
- Perceive risk situations in daily work as an opportunity to highlight the risk and take appropriate measures to prevent harm to patients.
- Respond positively to the expression of warnings or concerns by other health care professionals (of the same level or higher) in relation to patient safety.
- Maintain a positive attitude toward warning other health care professionals if, with their actions, they are ignoring an important patient safety rule.
- Be willing to openly and directly share specific proposals to improve patient safety.
- Be willing to learn from mistakes and patient safety incidents in which other professionals have been involved instead of judging them.

**Skills—in my opinion, internships in my work environment provide trainees with the competency to:**

- Communicate openly and directly to other professionals (of the same level or higher) specific concerns about patient safety by presenting information, asking questions, or expressing opinions.
- Request the responsible professionals’ advice to report, in the appropriate system, the occurrence of a patient safety incident that has been witnessed and make the report (if necessary).
- Warn assertively another health care professional (of the same level or higher) that, with their actions, they are ignoring an important patient safety rule.
- Respond assertively to the negative reaction of a health care professional (of the same level or higher) whom they have warned about ignoring an important patient safety rule.
- Verbally support and reinforce the initiative of other health care professionals (of the same level or higher) to share their specific concerns about patient safety with the rest of the team.
- Set and communicate concrete proposals to improve patient safety in their own unit or service.
- Offer peer support to a colleague involved in an adverse event to reduce the second victim syndrome (characterized by feelings of guilt, inadequacy, anxiety, shame, hypervigilance, or grief).

**Interventions—my health care institution:**

- Implements a training program for new staff (especially trainees) to foster a positive patient safety culture and a psychological safety climate.
- Appoints an influential group of people to design an intervention plan to foster a trusting climate among health care professionals to ensure patient safety.
- Holds regular clinical sessions with trainees to share patient safety concerns and lessons learned. This measure translates into the set of shared spaces to exchange experiences on patient safety incidents, devise barriers to minimize risks, and provide emotional and instrumental support among peers.
- Raises awareness among the center’s professionals, with the collaboration of heads of service, of the need to encourage trainees and colleagues to express their concerns regarding patient safety openly and directly and warn other professionals of the risks they identify in their daily work.
- Raises awareness among the center’s professionals, with the collaboration of heads of service, of the importance of responding positively to warnings from other professionals regarding compliance with relevant patient safety rules and reinforcing the open expression of specific patient safety concerns by trainees.
- Provides trainees with the opportunity to participate as observers during the planning of adverse event disclosure conversations with the affected patient and family.
- Allows trainees to have the opportunity to be present during the discussion and analysis following a patient safety incident.
- Provides trainees with specific training on reporting patient safety incidents through appropriate means.
- Offers institutional support to health care professionals involved in an adverse event to contribute to better safety at the workplace.

#### Statistical Analysis

For each item, the mean, SD, and coefficient of variability (CV) were obtained. We considered that those competencies with a score of <3.4 in acquisition required more effort to teach during trainees’ internships. These areas were selected to explore possible recommended actions for fostering the acquisition of psychological safety competencies among health care students and residents in a second consultation.

### Phase 2: Exploration of Measures and Actions to Promote the Acquisition of Psychological Safety Competencies Among Trainees (Open-Ended Survey Questions)

#### Procedure

On the basis of the results of the web-based consensus conference, a survey of 3 open-ended questions was developed to explore recommended actions to foster the learning of the psychological safety competencies necessary for trainees to be able to communicate their patient safety concerns or initiatives, observe another health care professional ignoring an important patient safety rule and assertively warn them of the risks of their behavior, and support a colleague who is emotionally affected after being involved in an adverse event (Multimedia Appendix 2). The survey was administered on the web between January 2023 and July 2023 to mentors who had participated in the consensus conference.

#### Information Categorization and Analysis

For the processing of the information obtained in this second phase, we used the qualitative methodology of content analysis, which consists of systematically transforming a large amount of text into a highly organized and concise summary of the key findings through a process of abstracting the data in consecutive steps to move from manifest and literal content to latent meanings [43]. In total, 2 researchers were involved in the coding process. The first step consisted of reading and rereading the survey data to obtain a sense of the whole and gain a general understanding of what the participants were referring to in their responses. Then, the raw meaning units or ideas were coded into categories agreed upon by the 2 researchers. In a second round, the researchers grouped these categories into themes. Thus, the coding hierarchy used, from least to most abstract, was meaning units, categories, and themes. The coding of the raw data was carried out independently for each of the questions as, although they were related, they focused on different competencies and problem situations. The formation of themes was carried out through an iterative process and was determined by the productivity of ideas and the weight of categories in the set of each separate question. Thus, the same topic could be represented in one question by a single theme and in another by 2. However, once all the ideas per question had been coded, a consistency analysis was carried out to prioritize the themes for the study as a whole and establish interquestion consistency. For each category and theme, we specified the spontaneity (understood as the number of times that the same idea was repeated independently by more than one participant) and the number of countries whose participants agreed on the same idea. We also calculated the overall productivity of ideas and the relative importance of the different themes identified within each question.

## Results

### Phase 1: Degree of Acquisition or Implementation and Significance of Psychological Safety Competencies and Institutional Interventions (Consensus Conference)

A total of 173 mentors (response rate: 173/256, 67.6%) from 11 countries in the pan-European environment participated in the study. Table 1 shows the participants’ sociodemographic characteristics and other variables of interest for the study.

**Table 1:** Characteristics of participating mentors and their work centers (N=173).

			Values
**Country, n (%)**			
	Croatia	15 (8.7)	
	Estonia	24 (13.9)	
	Finland	12 (6.9)	
	Germany	18 (10.4)	
	Israel	4 (2.3)	
	Lithuania	17 (9.8)	
	Malta	10 (5.8)	
	Portugal	15 (8.7)	
	Serbia	15 (8.7)	
	Slovakia	20 (11.6)	
	Spain	23 (13.3)	
**Sex, n (%)**			
	Female	129 (74.6)	
	Male	44 (25.4)	
**Age (y), mean (SD)**			43.6 (9.9)
**Years being responsible for trainees, mean (SD)**			10.7 (7.5)
**Number of personally supervised or mentored trainees in the last 3 years (2019-2021), mean (SD)**			23.1 (45.4)
**Professional profile, n (%)**			
	Medicine	72 (41.6)	
	Nursing	60 (34.7)	
	Pharmacy	24 (13.9)	
	Midwifery	4 (2.3)	
	Physiotherapy	3 (1.7)	
	Psychology	4 (2.3)	
	Dentistry	1 (0.6)	
	Microbiology	1 (0.6)	
	Occupational health care	1 (0.6)	
	Public health and organization	1 (0.6)	
	Radiology	2 (1.2)	
**Setting of clinical and mentoring performance, n (%)**			
	Primary care	37 (21.4)	
	Specialized care (hospital)	132 (76.3)	
	Social care	4 (2.3)	
**Specific patient safety training program in place at the center, n (%)**			
	Yes	48 (27.7)	
	No	125 (72.3)	

Regarding the degree of acquisition of psychological safety competencies among the trainees during their internships, the mean scores assigned by the mentors ranged from 2.9 to 3.8 points, suggesting a medium to low level of assimilation (global mean 3.4, SD 0.8; knowledge mean 3.4, SD 0.8; attitude mean 3.5, SD 0.8; skills mean 3.3, SD 1.0). Concerning knowledge, mentors believed that trainees had difficulties in knowing how to assertively warn another health care professional (of the same or a higher level) of the risk of ignoring an important patient safety rule (mean 3.1, SD 1.0; CV=33.1) and how to deal constructively with their possible bad reaction (mean 2.9, SD 1.1; CV=38.6). Mentors perceived among their trainees a medium level of development of attitudes favorable to creating a psychological safety climate. The aforementioned knowledge gap was expected to be reflected in the trainees’ lack of skills to warn others about unsafe practices (mean 3.2, SD 1.1; CV=35.6) and manage the possible interpersonal conflict arising from such verbalization (mean 3.1, SD 1.2; CV=37.8). In the mentors’ opinion, another of the least trained skills was offering peer support to a colleague involved in an adverse event to prevent or minimize the second victim response (mean 3.1, SD 1.3; CV=41.5).

Without being high, the most widespread knowledge among trainees in the eyes of their mentors was the understanding that open and direct communication of patient safety concerns could prevent adverse events (mean 3.8, SD 1.0; CV=25.0) and the distinction between situations that could cause avoidable harm to the patient and those that pose no risk (mean 3.7, SD 0.8; CV=22.3). Consequently, mentors noted that the most prevalent attitude among residents and students was the commitment to identify and prevent patient safety risks (mean 3.8, SD 1.0; CV=25.3). Of all the skills explored, consulting and sharing patient safety concerns obtained the highest score from mentors, although not reflecting a high acquisition level (mean 3.5, SD 1.1; CV=30.5).

In general, the significance assigned by mentors to the different psychological safety competencies was high, with mean scores ranging from 4.4 to 4.8 points (global mean 4.6, SD 0.6; knowledge mean 4.5, SD 0.6; attitude mean 4.6, SD 0.6; skills mean 4.6, SD 0.7). According to the mentors’ opinions, the most relevant components of psychological safety competencies included understanding the importance of openly communicating patient safety concerns to prevent adverse events (mean 4.8, SD 0.6; CV=25.0), adopting a firm commitment to identify and proactively manage patient safety risks (mean 4.7, SD 0.7; CV=14.9), showing a willingness to learn from safety incidents involving other professionals in a nonjudgmental manner (mean 4.8, SD 0.6; CV=13.2), and openly communicating one’s patient safety concerns regardless of the hierarchical level of the recipient (mean 4.7, SD 0.7; CV=15.0).

According to the mentors, the level of implementation of actions aimed at promoting the acquisition of psychological safety competencies among trainees in their institutions was low (mean 2.6, SD 1.1; range 2.4-2.8). Despite its perceived importance, mentors indicated that the least implemented action was holding regular clinical sessions with trainees to share patient safety concerns and lessons learned (mean 2.4, SD 1.3; CV=56.9). Overall, mentors rated the 9 institutional interventions explored in the questionnaire as highly significant (mean 4.4, SD 0.8; range 4.2-4.6). Among the interventions assessed as most significant were implementing a patient safety and psychological safety training program for new trainees (mean 4.6, SD 0.9; CV=19.0) and offering institutional support to trainees involved in an adverse event (mean 4.6, SD 0.9; CV=20.0).

The results of the descriptive analysis (mean, SD, and CV) for each questionnaire item are shown in the tables in Multimedia Appendix 3.

### Phase 2: Exploration of Measures and Actions to Promote the Acquisition of Psychological Safety Competencies Among Trainees (Open-Ended Survey Questions)

#### Overview

In this second consultation, 36.4% (63/173) of the mentors who completed the phase 1 questionnaire participated. The participating countries were Croatia, Estonia, Germany, Portugal, Serbia, Slovakia, and Spain. The overall productivity was 353 ideas or meaning units grouped into 9 joint themes. More than half (210/353, 59.5%) were related to training activities, environmental and structural conditions (safety culture and work climate), and individual attitudes. The rest of the proposals referred to the trainees’ reference person, institutional resources to support the second victim, curricula content and training pathways, systems and mechanisms to give trainees a voice, institutional management of clinical risks (incident reporting and analysis), regulations and standards, supervision, and resources to support trainees (Table 2).

**Table 2:** Productivity and distribution of meaning units by theme overall and per question (N=353).

Theme	Spontaneity (relative priority), n (%)			
	Question 1: communicate patient safety concerns (n=154)	Question 2: warn about an unsafe behavior (n=101)	Question 3: support a second victim (n=98)	Total
Training	46 (29.9)	52 (51.5)	29 (29.6)	127 (36)
Individual attitudes and environmental conditioning factors^a^	35 (22.7)	23 (22.8)	25 (25.5)	83 (23.5)
Person of reference	19 (12.3)	6 (5.9)	15 (15.3)	40 (11.3)
Institutional resources to support second victims	—^b^	—	27 (27.6)	27 (7.6)
Curricula	16 (10.4)	7 (6.9)	—	23 (6.5)
Systems and mechanisms to give a voice to trainees	20 (13)	—	—	20 (5.7)
Institutional risk management, patient safety reporting systems, and incident analysis	8 (5.2)	5 (5)	—	13 (3.7)
Regulations, guidelines, protocols, standards, and policies	10 (6.5)	—	2 (2)	12 (3.4)
Supervision and resources to support trainees	—	8 (7.9)	—	8 (2.3)

^a^In question 1, this theme comprises themes 1.2—safety culture (spontaneity: n=26) and 1.7—organizational structure and culture (spontaneity: n=9).

^b^This theme did not emerge among the ideas proposed by the participants in response to this question.

Independent analysis of each of the questions yielded an individual productivity of 154 ideas for the first question (communication of patient safety concerns or initiatives) classified into 8 themes and 26 categories, 101 ideas for the second question (observing another health care professional ignoring an important patient safety rule and assertively warning them about the risks of their behavior) classified into 6 themes and 22 categories, and 98 ideas for the third question (offer support to a colleague suffering emotionally after being involved in an adverse event) classified into 5 themes and 16 categories. Multimedia Appendix 4 presents a summary figure with the main results of the qualitative analysis and a set of tables with the coding of the ideas per question with the specification of spontaneity, the number of participating countries per theme and category, and some examples of meaning units.

The following is a brief presentation of the themes that emerged from the analysis of the information provided by the mentors in this second phase of the study. With a practical vision and purpose, the relevant information was transformed into recommendations for fostering the learning of psychological safety competencies among trainees in clinical settings (Textbox 2).

**Recommendations for fostering the learning of psychological safety competencies among trainees in clinical settings extracted from the information provided by mentors in the study’s second phase.**

**Training**

- What:
  - Focus on patient safety, interpersonal communication, assertiveness, active listening, and social support skills.
  - Include emotional intelligence, critical thinking, reflective skills, argument formation, and achievement recognition.
  - Train in clinical interviewing skills, using open and closed questions and standardized questionnaires.
- How:
  - Implement innovative strategies such as seminars, lectures, workshops, simulation learning, role-playing, case studies, videos, panel discussions, clinical sessions, and debriefings.
  - Provide ongoing training tailored to the health care context rather than one-off sessions.
  - Practice these skills in real-world scenarios, including daily professional exchanges, challenging conversations, and conflict resolution.
  - Address communication in the context of patient safety events to prevent risks and ensure safety.
- For whom:
  - Sensitize and train the entire health care organization, including teams, mentors, and management.
  - Implement top-down training strategies, starting with senior management. Ensure that managers and team leaders receive regular compulsory training in patient safety, leadership, and staff management.
- Who:
  - Use psychologists for communication and interpersonal support skill training.
  - Engage patient safety experts for specialized training.
  - Supervision by mentors should cover communication skills, interactions with professionals, patient safety information collection, and reflection on clinical risks.
- When:
  - Begin introductory training in patient safety, communication, and teamwork skills upon joining the center.
  - Ensure comprehensive and continuous training throughout the professional career regardless of professional profile to maintain a shared understanding among all team members.

**Individual attitudes and environmental conditioning factors**

- *Supportive, respectful, and trustful climate:*
  - Leaders and middle managers should foster positive relationships, teamwork, openness, trust, honest communication, and mutual support.
  - Inclusive and ethical leadership helps integrate trainees into work teams, allowing them to ask questions without fear and express views assertively.
  - Organize social and informal activities to improve team relationships.
- *Positive attitudes toward patient safety and peer support:*
  - Cultivate respect and equality among professionals to facilitate the understanding that everyone’s contributions are relevant for quality and safe care.
  - Foster a positive attitude toward seeking and providing peer support in challenging situations, addressing the second victim phenomenon.
- *Just and nonpunitive safety culture:*
  - Move away from a punitive and blame culture; adopt a systemic approach to adverse events and honest mistakes to prevent recurrences.
  - Recognize human fallibility and the multifactorial origins of patient safety incidents.
  - Encourage open communication without fear of punishment, judgment, stigma, or rejection.
  - Middle managers, mentors, supervisors, and senior professionals must act as role models and change agents, promoting a just safety culture.
  - Launch internal campaigns to create a just culture and make patient safety a regular topic of conversation.
- *Open and constructive communication channels:*
  - Promote assertive expression and acceptance of constructive criticism as learning opportunities.
  - Establish direct communication channels between all team members.
- *Leadership and management involvement:*
  - Leaders and managers should actively seek feedback and suggestions before initiating procedures.
  - Encourage managers and superiors to share personal experiences with adverse events to demonstrate that these issues are a reality in clinical practice.
  - Appoint an institutional patient safety officer and ensure that trainees are aware of their presence.
- *Structural measures to enhance patient safety:*
  - Promote a culture in which all team members regardless of hierarchy are encouraged to contribute to patient safety.
  - Use cocreated checklists to standardize practice and establish a common language across different roles.

**A reference person for trainees**

- *Role and responsibilities of mentors:*
  - Mentors are ideal figures to establish trust and promote patient safety supported by institutional resources.
  - Act as role models, explaining safety rules, managing risks, and using tools (eg, reporting systems).
  - Define standard behaviors in critical scenarios: safety concerns, adverse events, mistakes, noncompliance with safety rules, unsafe acts, and emotional distress.
- *Support and accessibility:*
  - Mentors should be approachable, providing support, information, and guidance.
  - Link trainees with their teams and institutional resources, guiding interactions with other professionals.
  - Support trainees as peer supporters during emotional distress from incidents.
- *Training and resources:*
  - Mentors need specialized training, resources, and mechanisms for supervising trainees.
  - Legislation may be needed to create specific supervisor positions in medical institutions.

**Institutional resources to support the second victim**

- *Support program and format:*
  - Raise awareness and train professionals to act as peer supporters providing emotional first aid through individual meetings or confidential group debriefings.
  - Focus on active listening, emotional validation, positive language, empathy, and companionship without investigating the event.
  - Establish multidisciplinary peer teams with personal experience of such events for a sense of identification.
  - Organize Balint groups for critical and self-reflective discussions on emotional reactions to challenging situations facilitated by a psychotherapist [44].
  - Ensure availability of psychological support through mental health, occupational health, or risk prevention structures.
  - Provide clear referral channels to connect grieving trainees with resources, with mentors acting as bridges.
- *Institutional programs for staff well-being:*
  - Implement programs promoting employee well-being and personal development.
  - Host social and recreational activities such as service dinners and hiking.

**Curricula**

- *Incorporate patient safety into education:*
  - Integrate patient safety and communication skill training into health care degree programs, postgraduate studies, specialization pathways, and doctoral studies.
  - Foster cooperation between academia and health care training institutions for comprehensive competency development.
- *Guidelines and regulations:*
  - Establish European and national guidelines to systematically regulate and implement patient safety training in all health faculties in alignment with World Health Organization recommendations [29].
- *Assessment and certification:*
  - Include patient safety competency assessment, including communication skills, in state and certification tests using methodologies such as the objective structured clinical examination.

**Systems and mechanisms to give trainees a voice (highlights)**

- *Create common spaces or forums:*
  - Hold regular meetings to discuss patient safety cases, significant issues, and preventive initiatives.
  - Involve all relevant parties: supervisors, mentors, professors, residents, and students.
  - Use forums for supervisors to share personal experiences and learning.
- *Encourage patient safety ownership:*
  - Foster ownership and meaning in trainees’ work to encourage communication on patient safety.
  - Involve trainees in supportive supervision roles and patient safety projects.
- *Collect trainee feedback:*
  - Conduct regular one-to-one mentor-trainee interviews.
  - Create an anonymous mailbox for concerns and suggestions.
  - Maintain a trainee diary to document experiences and feedback.

**Institutional risk management, patient safety reporting systems, and incident analysis**

- *Anonymous reporting systems:*
  - Create systems for anonymously reporting adverse events.
  - Focus on proposing improvement plans with corrective, not punitive, measures.
  - Ensure legal certainty for reporters to mitigate fear of legal repercussions.
- *Trainee involvement in incident analysis:*
  - Allow trainees to be participant observers in the incident analysis process.
  - Ensure that trainees receive feedback on their reports and the resulting preventive and corrective measures.
- *Debriefing and seminars:*
  - Conduct joint and individual debriefings or seminars for trainees involved in critical incidents to review what happened.

**Regulations, guidelines, protocols, standards, and policies**

- *Institutional level:*
  - Ensure the availability of standardized protocols for safe clinical procedures, emphasizing risk detection and prevention measures.
  - Establish formal channels and communication procedures to enhance patient safety.
  - Structure patient safety rules hierarchically: elemental and mandatory for all professionals and complementary for specific procedures or situations.
  - Reinforce the center’s quality and safety policy supporting the health care quality unit.
- *National level:*
  - Advocate for national legislative changes to ensure legal protection for professionals and trainees involved in patient safety processes (eg, incident reporting and open disclosure).
  - Develop and implement accreditation standards for certifying health care institutions as patient safety promoters in teaching and training.

**Supervision and resources to support trainees’ learning process**

- Conduct regular interviews, knowledge checks, and feedback sessions during joint practice.
- Use clinical scenarios to prepare and execute procedures under safe conditions.
- Encourage trainees to detect errors and practice providing feedback.

#### Training

Most of the proposals made by the mentors to encourage trainees to acquire psychological safety competencies and develop skills in communicating patient safety concerns, warning a professional that their behavior compromises patient safety, and supporting a colleague who is a second victim were related to training actions.

The proposed ideas focused on the content, target audience, teaching methodologies, career stage, and parties involved in training.

#### Individual Attitudes and Environmental Conditioning Factors (Organizational Structure, Safety Culture, and Work Climate) That Determine Behavioral Patterns

The acquisition of psychological safety competencies by trainees and their commitment to patient safety seems to have a circular and bidirectional relationship with a positive safety culture and a work climate based on trust and respect in the institution. Most mentors identified these 2 contextual factors as prerequisites for trainees to openly discuss adverse events and honest mistakes and support each other.

#### A Reference Person for Trainees

In environments as changing, novel, and challenging as clinical settings, mentors mentioned the need for trainees to have a permanent reference person with whom they can establish a trusting bond from the moment they join the health care center.

#### Institutional Resources to Support the Second Victim

Concerning the approach to the second victim phenomenon, the mentors’ contributions referred to implementing institutional resources and programs to minimize the impact of adverse events on health care professionals, including trainees. Most of the proposals were along the lines of the reference programs based on the Scott Three-Tiered Interventional Model of Second Victim Support [45].

#### Curricula

The mentors agreed on the need to incorporate a subject on patient safety in the curricula of health care degree programs.

#### Systems and Mechanisms to Give Trainees a Voice

The mentors made several proposals to facilitate bottom-up communication and the expression of patient safety concerns and initiatives among trainees.

#### Institutional Risk Management, Patient Safety Reporting Systems, and Incident Analysis

The mentors agreed on the appropriateness of involving trainees in adverse event reporting and analysis to promote awareness of health care risks and the adoption of a clinical practice style committed to patient safety.

#### Regulations, Guidelines, Protocols, Standards, and Policies

As additional measures, the mentors highlighted the need to reinforce the institutions’ patient safety through regulations, protocols, and policies at the institutional (meso) and national (macro) levels.

#### Supervision and Resources to Support Trainees’ Learning Process

Mentors attached particular importance to supervision as a mechanism for training students and residents in psychological safety competencies.

## Discussion

### Principal Findings

Our study’s first objective was to find out whether health care trainees in a pan-European environment acquire, from the point of view of their mentors, competencies in psychological safety during their internships in clinical settings. This study’s results show that, according to the mentors, the competency acquisition level is moderate to low, so in their opinion, the training currently offered in the pan-European context does not guarantee the systematic acquisition of the competencies needed to foster a psychological safety climate in health care institutions. A total of 40% (8/20) of the competency elements analyzed presented a low acquisition value, being more pronounced in the psychomotor domain (skills). Such lack of KAS reported by mentors can prevent residents and health care students from engaging in challenging conversations in clinical settings (eg, warning a senior professional that their behavior poses a risk to the patient, communicating concerns and initiatives, or asking questions related to patient safety) or supporting a colleague who is suffering emotionally after their involvement in an event that caused or could have caused harm to a patient.

A second question not initially raised explicitly in our study but that emerged from the first-phase findings and connects them to the second objective concerns the reasons for the low acquisition of psychological safety competencies among pan-European health care trainees. In the opinion of the mentors, the optimal development of these competencies is hampered by deficiencies in formal patient safety content in health care curricula and training pathways, modeling by trainers, trust, cohesion, team spirit (safe people and safe environments), decentralization of the health care institutions’ structure, specific patient safety policies, and institutional resources to support the creation of a psychological safety climate and a proactive patient safety culture.

In response to what needs to be done to promote the training and learning of psychological safety competencies, the mentors offered an extensive list of wide-ranging measures and recommendations to address the deficiencies identified. These proposals ranged from specific practices, methodologies, models, and content for training in psychological safety competencies to higher-level measures related to the organization’s structural, strategic, cultural, and environmental aspects.

These findings support a conceptualization of psychological safety competencies based on the integration of purely academic and formative actions with others of an institutional nature aimed at fostering cultural patterns and work climates that encourage the manifestation of these competencies. In their proposals, the mentors emphasized that both pillars are essential for trainees to show optimal performance in psychological safety. In this sense, an exclusively academic approach is doomed to failure as, if the health care environment does not allow for the implementation of what has been learned, competence acquisition will only be possible in the cognitive and affective domains but not the psychomotor one. The framework that we present in this study for measuring competencies in psychological safety is, to our knowledge, the first to specify what a competent trainee in psychological safety would look like, exemplifying not only what they should know and feel (aspects that are not directly observable) but also what they should do and what others can observe to evaluate their competence.

### Comparison With Prior Work

The perception of the mentors in our study is congruent with the levels of psychological safety reported by pediatric nurses and residents in the American context (mean of 3.4 points on a scale of 1-5) [46]. They identified the following as the main barriers to a psychological safety climate in the team: difficulties in interpersonal relationships between professionals of different disciplines and statuses, unsatisfactory communication style and frequency, inadequate resolution of disagreements, work overload accompanied by lack of collaboration, and interpersonal disrespect. The mentors in our study addressed some of these issues in their proposal for measures to promote psychological safety competency training.

As our study suggests, psychological safety and patient safety are closely linked elements of the clinical environment and practice. The mentors who participated in this study’s second phase related the trainees’ low competence in psychological safety to the lack of formal incorporation of patient safety into the curricula of health care degrees. This result is in line with the findings of Sánchez-García et al [36] in the pan-European context, which show that there is still a long way to go in adapting the curricula as half of the nursing schools and 60% of the medical schools analyzed did not cover any topics related to patient safety. In those cases in which the curricula did cover patient safety aspects, interpersonal communication, quality of care, and other elementary aspects were the most widespread topics. The second victim phenomenon was formally present in only 1 of the 206 curricula reviewed.

The relationship between psychological safety and patient safety has been extensively studied. Research with nurses has shown that psychological safety predicts the intention to report safety incidents and a greater willingness to engage in open communication, which, in turn, may lead to higher job satisfaction, lower turnover intention, and improved patient safety [47,48]. In this line, Dietl et al [25] observed that the positive effect of psychological safety on patient safety is mediated by interpersonal communication in the team. Therefore, not surprisingly, training in communication skills and teamwork was one of the targets for action identified by the mentors who participated in our study.

Other studies have also suggested that this relationship may run in the opposite direction (ie, a positive safety culture in the health care institution may contribute to developing a psychologically safe work climate). Along these lines, O’Donovan and McAuliffe [49] identified the enablers of psychological safety across the individual, team, and organizational levels of health care institutions, including safety culture and continuous improvement. Our mentors provided solutions related to some of these facilitators. On an individual level, participants highlighted the role of mentors and supervisors in fostering professional responsibility among trainees, recognizing them as full team members. This aspect relates at the team level to the factors of leader behavioral integrity and status, hierarchy, and inclusiveness that our mentors recommend fostering by creating shared spaces free of hierarchical differences. For this purpose, Ulmer et al [50] present the “Mistake Of The Week” initiative, which consists of a weekly semistructured conference in which health care staff are encouraged to voluntarily disclose their mistakes and near misses based on 4 pillars of success, namely, exemplification, fixed time slots and clearly defined dynamics, absence of fear of punishment, and trusting atmosphere. This initiative recalls the morbidity and mortality conferences (M&Ms) widely implemented in North America and included in US residency programs. These conferences aim to critically analyze and discuss safety incidents in a safe environment. When these conferences are cross-cutting, they allow for the participation of different institutional agents in the discussion of incidents, including from students and junior staff to senior leaders or administrators. This model increases the likelihood that M&Ms will become a tool for system-wide improvement [51]. Although presented as an opportunity for learning and improving the quality of care, their inappropriate use can also lead to undesirable results [52]. In Europe, although less widespread, M&Ms are present in surgical services, intensive care units, and emergency departments in some countries, such as Germany, France, the Netherlands, and the United Kingdom [53-56]. Although stakeholders value the M&Ms’ implementation positively, there remains a lack of objective outcome measures to determine their impact on patient safety and system-wide improvement and some challenges that jeopardize the effectiveness of these conferences [57]. Although expectations are clear (focus on education and quality improvement, lack of blame, being mandatory for residents and attendings, and orientation toward changes in clinical practices), excessive heterogeneity and lack of structure often limit their impact [53,55]. The European experience suggests better results when M&Ms are interprofessional; incorporate a moderator; are supported by a quality committee; are incorporated as part of the Plan, Do, Check, Act cycle; and include the use of validated instruments for collecting data on complications [53,55-57]. As occurs in the case of psychological safety competency training, clinician engagement, patient safety culture, and organizational governance and leadership are identified as contributors to effective M&Ms [52]. Facilitators regarding peer support and familiarity of the leader and team members are closely related to the proposals of the mentors, who suggested the formal designation of a reference contact for the trainees with whom they can establish a trusting relationship, the creation of positive team dynamics, and peer support in case of an adverse event. Recently, Seys et al [58] proposed an international multidimensional action plan for second victim support structured in five levels: (1) prevention at the individual health care professional and organizational level, (2) self-care of the health care individual and team, (3) support through peers and triage, (4) structured professional support (eg, mental health or specialized support), and (5) clinical support (pharmacological treatment and long-term psychotherapy). The results of the study by Lyman et al [59] with newly graduated nurses reflect well on how individual factors facilitating psychological safety in clinical settings are built on team experience. Thus, the nurses said that their self-confidence was preserved when team members approached mistakes as opportunities for improvement. Finally, O’Donovan and McAuliffe [49] point out that, when a just safety culture exists in institutions, it is possible to create spaces where trainees and new staff find the courage to speak up. The mentors in our study identified the still widespread existence of “*name-blame-shame*” cultures as a critical barrier to acquiring and implementing psychological safety competencies by any health care team member, especially trainees. In this sense, safety culture is understood as a prerequisite for a psychological safety climate, with culture conceptualized as a more stable element and climate as a more situational and local outcome largely dependent on leadership styles and team dynamics that, in turn, are influenced by the organization’s values, norms, and behavioral patterns.

In addition to the attenuation of hierarchies and the establishment of high-quality relationships, McClintock and Fainstad [60] consider other aspects as core features of psychologically safe environments, such as a trainee-driven and flexible learning agenda, the absence of formal assessment, and time for debriefing. In the same direction, the mentors in our study recommended merging supervision and competency assessment with daily clinical practice and creating Balint groups [44] as team debriefings.

Apart from the importance of cultural aspects and the work environment already discussed, the acquisition of psychological safety competencies by trainees requires investing efforts and resources in wide-ranging training actions. According to the categories proposed by O’Donovan and McAuliffe [61] in their systematic review of interventions to improve psychological safety, speaking up, and voice behavior, the proposals of the mentors in our study were based on simulations, video presentations, case studies, workshops, forums, and meetings. The mentors particularly stressed the importance of adopting an organization-wide approach to training on psychological safety and patient safety. To have competent trainees, first, it is necessary to ensure that mentors are trained in patient safety, clinical risk management, communication, leadership, and supervisory skills. Minehart et al [62] implemented an educational intervention to improve the quality of feedback provided by anesthesia teachers. Those who received the training performed better in maintaining a psychologically safe environment and identifying and exploring trainees’ performance gaps.

Given this training gap, it is essential to draw up a general plan with specific guidelines to adapt the incorporation of this content into the training plans and specialization pathways of future health care professionals. As the mentors of our study pointed out, more than a decade ago, the World Health Organization [29] published guidelines for incorporating patient safety into curricula. However, national policies have not yet ensured the widespread implementation of these recommendations in pan-European countries, and as of now, only a few isolated universities have taken the initiative on a discretionary basis [36].

Although psychological safety has been widely recognized as part of successive patient safety and quality improvement processes, it remains a relatively unknown construct among many educators and trainees. Consequently, it is often relegated to the hidden curriculum that becomes tangible through mentors’ exhibited norms, values, and behaviors. This hidden curriculum can have both positive and negative effects on professional development. The positive effects manifest through empathy, resilience, perseverance, and psychological safety [63]. The way to prevent the negative consequences of the hidden curriculum and enhance psychological safety is to formalize the teaching of those values and norms that support safe practice and a clinical learning environment based on openness, trust, and respect.

This study provides an overview of how psychological safety competencies are being taught in pan-European clinical learning environments from the mentors’ perspective. It also highlights the gaps across the board in the competency training of health care trainees. The recommendations proposed by this group seek to reinforce the formal teaching of patient safety and psychological safety in a multifactorial and multilevel manner, including contextual, attitudinal, and educational elements. Psychological safety is fundamental to achieving learning health care organizations and functions as an enabler of the ability of the system and its teams to remain in a continuous improvement cycle that contributes to safer clinical environments for patients [49]. Therefore, efforts should be synergistically directed toward the simultaneous and bidirectional improvement of patient safety and psychological safety. This synergy is already envisaged in the World Health Organization curriculum guide [29] and Global Patient Safety Action Plan 2021 to 2030 [7], in which preserving the psychological safety of health care professionals by preventing harm to their well-being (eg, burnout) is linked to goal 3 of the United Nations Sustainable Development Goals. Furthermore, fostering psychological safety (eg, speaking up and stopping the line) is considered an enabling competency in the Canadian Patient Safety Institute’s proposed framework [64] for making patient safety a reality in health care institutions. Along these lines, the Institute for Healthcare Improvement has been working for decades on improving patient safety with a holistic approach, making a wide range of tools, training, and documentation available to health care institutions and professionals. One of the most noteworthy initiatives is its Certified Professional in Patient Safety credential, which establishes core standards for certifying a proficiency level of professionals in patient safety [65].

The mentors’ proposals in this study may be helpful to materialize in concrete actions the global solution that this problem needs. Some of these interventions have shown improvements in psychological safety, speaking up, and voice behavior; however, longitudinal and multifaceted studies to determine their effectiveness are still required [61]. On the other hand, the items of the ad hoc questionnaire used in the first phase of this study can serve as a prescriptive proposal of the KAS to be trained in residents and students for the development of psychological safety competencies and also as an instrument to assess their degree of acquisition.

Our study adds to previous research that supports that acquiring psychological safety and patient safety competencies requires a global and integrated effort that stems from the coordinated involvement of academia and the health care system and extends beyond merely training actions [48,50,61]. Educating those still in the training process on psychological safety means tackling the problem from the ground up with a commitment to the future. It is important to remember that trainees are not solely responsible for creating a psychological safety climate, at least not initially. However, they can still have a significant impact as a driving force for change. The synergistic combination of training and structural measures at the individual, team, and organizational (culture) levels is the key to a psychologically safe environment in clinical settings [48].

### Limitations

Despite the merits of this study, it is important to acknowledge a few limitations. Study participants were recruited through convenience sampling. They were selected by national coordinators who are members of the European ERNST Consortium, so their sensitivity to patient safety and psychological safety topics may be higher than that of the average health care trainee mentor in the pan-European context. Therefore, the sample may not have been representative of the study population. Most of the study participants (129/173, 74.6%) were women. Although this large gender discrepancy in the respondent distribution might suggest a possible representation bias, according to data from the European Commission, 78% of health care workers in the third quarter of 2020 were female [66]. Thus, our sample reflects the current picture as far as gender distribution is concerned. Similarly, there may have been discrepancies in how mentors understood psychological safety or their level of familiarity with the topic. On the other hand, we did not control for possible differences between countries in curricula or the structure and functioning of the academic and health care systems. When generalizing these results, it is necessary to consider the impact of international accreditations on medical programs that include curriculum elements focused on patient safety. These aspects may have affected the mentors’ experience and familiarity with patient safety, psychological safety, and the second victim phenomenon. For some participants in this study’s first phase, the questionnaire items were too specific, which indicated difficulty in discriminating between the core components of competencies (KAS) and may have affected the quality of the responses. In the second phase, the response rate dropped drastically, and only 55% (6/11) of the original countries participated. To prevent the representativeness of the results from being affected by experimental mortality, only those countries that ensured the participation of at least 5 mentors were encouraged to be involved in the second phase of this study. Furthermore, the higher productivity of ideas in the first question of the final survey may be because participants were more familiar with the communication aspects, whereas the issue of second victims and initiatives to address it are less known. Alternatively, there may have been a demotivation effect that caused lower productivity in the later questions. As for the study population, the choice of mentors, although justified, only offers a partial view of the level of acquisition of psychological safety competencies by health care trainees. The vision of the students and residents, who are the protagonists of the learning process, may differ, and future studies should directly survey this group. Finally, although the study offers an extensive and exhaustive list of actions to improve psychological safety and patient safety in clinical settings, the proposal was not prioritized or ordered sequentially, so centers wishing to improve these aspects may find it challenging to decide where to start. The answer to this question can be affected by multiple secondary issues such as resource availability, leadership involvement, or facility and staff resistance. While cultural change at the institutional level is essential to ensure psychologically safe clinical environments, changing values and beliefs takes time and may be more effectively achieved through concrete actions and behavioral adjustments even if these are initially short range. In any case, future research should address prioritizing actions and establishing indicators and compliance standards involving the different stakeholders.

### Conclusions

To our knowledge, this study is the first to address how psychological safety competencies are being taught to future health care professionals from the point of view of mentors and with a pan-European scope. Our study results showed a medium to low level of acquisition of psychological safety competencies among health care trainees in a pan-European setting as perceived by their mentors. According to this group, the solution to this competency gap should be comprehensive and consider the following aspects: training in communication skills and patient safety, environmental conditioning factors (safety culture and work climate) and individual attitudes, a reference person for trainees, formal incorporation into the curricula of health care degree programs and specialization pathways, specific systems and mechanisms to give trainees a voice, institutional risk management, regulations, guidelines and standards, supervision, and resources to support trainees.

The results of our study emphasize the importance of taking multiple actions to establish psychological safety in clinical environments. Academia should seek to formally teach psychological safety competencies during formal training by incorporating them into the curriculum and using innovative teaching methodologies based on technological tools and solutions. It should also strengthen communication and coordination mechanisms with the clinical institutions where trainees perform their internships and maintain a contact person in academia to assist them during the process and supervise their experience. Health care institutions, for their part, should actively promote a just safety culture free of blame and punishment by providing training in patient safety and psychological safety to all members of their staff with the commitment and direct involvement of the management. Mentors should receive specialized education to train trainees in patient safety and promote psychological safety behaviors such as speaking up. They should also supervise the development of these competencies in the trainees under their charge and be their reference person. As for trainees, they should develop from the beginning of their careers a solid commitment to patient safety and a willingness to speak openly about their patient safety concerns and initiatives and stay in a cycle of learning and improvement. They must learn that this commitment is not to themselves but to patients and the delivery of quality care.

The proposal for measures described in this study aims to facilitate the translation of international guidelines into practice and clinical settings in the pan-European context. Further research on the combined effectiveness of these measures is needed to achieve competent trainees and health care professionals in psychological safety and patient safety. Psychological safety is critical in creating learning health care organizations and safer clinical environments for patients.

## Appendix

Multimedia Appendix 1 Questionnaire used in the first phase of the study (web-based consensus conference).

Multimedia Appendix 2 Open-ended survey used in the second phase of the study.

Multimedia Appendix 3 Descriptive analysis (mean, SD, and coefficient of variability) of the degree of acquisition or implementation and significance of psychological safety competencies and institutional interventions (consensus conference).

Multimedia Appendix 4 Content analysis: themes, categories, and examples of meaning units by core questions.

## Abbreviations

COREQ: Consolidated Criteria for Reporting Qualitative Research
CV: coefficient of variability
ERNST: European Researchers’ Network Working on Second Victims
KAS: knowledge, attitudes, and skills
M&M: morbidity and mortality conference
