# Correction: Psychological Safety Competency Training During the Clinical Internship From the Perspective of Health Care Trainee Mentors in 11 Pan-European Countries: Mixed Methods Observational Study

**DOI:** 10.2196/68503

**Published:** 2024-11-15

**Authors:** Irene Carrillo, Ivana Skoumalová, Ireen Bruus, Victoria Klemm, Sofia Guerra-Paiva, Bojana Knežević, Augustina Jankauskiene, Dragana Jocic, Susanna Tella, Sandra C Buttigieg, Einav Srulovici, Andrea Madarasová Gecková, Kaja Põlluste, Reinhard Strametz, Paulo Sousa, Marina Odalovic, José Joaquín Mira

**Affiliations:** 1 Department of Health Psychology Miguel Hernández University of Elche Elche Spain; 2 Department of Health Psychology and Research Methodology Faculty of Medicine Pavol Jozef Šafárik University Kosice Slovakia; 3 Tartu Health Care College Tartu Estonia; 4 Wiesbaden Institute for Healthcare Economics and Patient Safety (WiHelP) Wiesbaden Business School RheinMain University of Applied Sciences Wiesbaden Germany; 5 Public Health Research Centre National School of Public Health NOVA University Lisbon Lisbon Portugal; 6 Comprehensive Health Research Center National School of Public Health NOVA University Lisbon Lisbon Portugal; 7 University Hospital Centre Zagreb University of Zagreb Zagreb Croatia; 8 Pediatric Center, Institute of Clinical Medicine Faculty of Medicine Vilnius University Vilnius Lithuania; 9 BENU Pharmacy PHOENIX Group Serbia Belgrade Serbia; 10 Faculty of Social and Health Care LAB University of Applied Sciences Lappeenranta Finland; 11 Department of Health Systems Management and Leadership Faculty of Health Sciences University of Malta Malta Malta; 12 Cheryl Spencer Department of Nursing University of Haifa Haifa Israel; 13 Institute of Applied Psychology Faculty of Social and Economic Sciences Comenius University Bratislava Bratislava Slovakia; 14 Institute of Clinical Medicine University of Tartu Tartu Estonia; 15 Faculty of Pharmacy University of Belgrade Belgrade Serbia; 16 Foundation for the Promotion of Health and Biomedical Research of the Valencia Region (FISABIO) Sant Joan d'Alacant Spain

In “Psychological Safety Competency Training During the Clinical Internship From the Perspective of Health Care Trainee Mentors in 11 Pan-European Countries: Mixed Methods Observational Study” (JMIR Medical Education 2024;1(10): *e64125*) the authors made one revision.

The following section in the Acknowledgments section:

This paper was based on work from the European Cooperation in Science and Technology Action 19113, The European Researchers’ Network Working on Second Victims, supported by the European Cooperation in Science and Technology [67]

Has been corrected to:

This article is based upon work from COST Action, TheERNSTGroup, CA19113, supported by COST (European Cooperation in Science and Technology).

Additionally, the associated reference [67] (below) will be removed from the Reference List, as it will no longer be cited in the paper.

67. Home page. COST Association. URL: https://www.cost.eu/ [accessed 2024-04-29]

The correction will appear in the online version of the paper on the JMIR Publications website on November 15, 2024, together with the publication of this correction notice. Because this was made after submission to PubMed, PubMed Central, and other full-text repositories, the corrected article has also been resubmitted to those repositories.

