# Supplementary material for: Psychological Safety Competency Training During the Clinical Internship From the Perspective of Health Care Trainee Mentors in 11 Pan-European Countries: Mixed Methods Observational Study
Source: JMIR Med Educ. 2024 Oct 7;10:e64125. doi: 10.2196/64125 (PMC11494257; doi:10.2196/64125)
Supplement: Multimedia Appendix 1 [file mededu_v10i1e64125_app1.docx]

**Multimedia Appendix 1. Questionnaire used in the first phase of the study (web-based consensus conference).**

**ENGLISH VERSION**


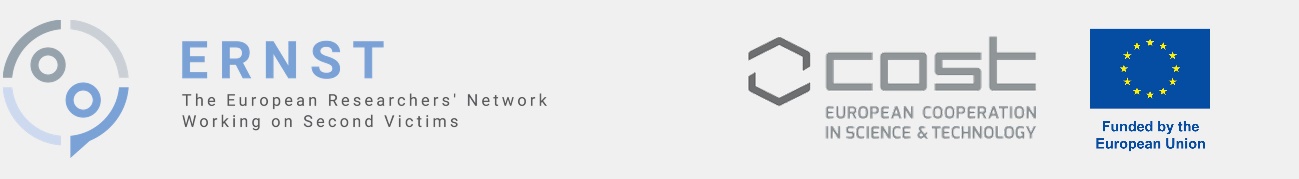


**Competencies in healthcare discipline trainees to promote a climate of psychological safety in clinical settings**

Thank you for agreeing to participate in this study on psychological safety competencies acquired by residents and students of health disciplines during their training period.

We are a team of researchers from the academic and clinical fields, part of the European ERNST Consortium focused on patient safety and second victims’ studies. This study involves researchers from Croatia, Estonia, Finland, Germany, Israel, Lithuania, Malta, Portugal, Serbia, Slovakia, and Spain.

Psychological safety refers to the shared belief that a work team can face complex challenges when there is an environment of mutual respect and trust. Thus, healthcare professionals provide safer care when causes and ways to avoid clinical errors can be discussed and analysed without fear of criticism or sanctions. A psychological safety climate can be a determinant of patient safety. In this way, this provides safer care when it is possible to discuss and analyse the causes and mechanisms to avoid clinical errors without fear of criticism or sanctions.

This Delphi study explores:

- what psychological safety competencies (knowledge, attitudes, and skills) are being acquired by future generations of healthcare professionals (students and residents hereafter referred to as “trainees”) through their clinical internships,
- what actions the healthcare institutions in which the trainees do their clinical internships should implement to promote the acquisition of these competencies.

The responses to the questionnaire will be analysed in a pseudo-anonymized and aggregated manner. There are no right or wrong answers to the questions, but the purpose is to know your perception as a trainee mentor. The questionnaire will take approximately 10 minutes to complete. Please do not forget to send your answers at the end of the questionnaire.

For any questions or comments, please send an e-mail to your national coordinator (who invited you to participate in the study).

We thank you in advance for your cooperation and your responses.

**Informed consent**

Participation in this study is voluntary. The data collected will be pseudo-anonymized and analysed in aggregate form. The platform is hosted on a secure server located in Spain and managed by Miguel Hernández University, part of the Spanish public education system. Please, confirm that you are participating in this study on a voluntary and informed basis. You may revoke your consent to participate in the study at any time by sending an email to [____________](mailto:calite@umh.es)

I consent to participate in the study on a voluntary and informed basis.

**1. COMPETENCES. Knowledge**

Please, assess each competency (knowledge) according to two criteria:

*(1) Degree of acquisition*. To what degree are trainees currently acquiring this competency through their clinical internships at your institution?

1 No acquisition at all – 5 Fully acquired

*(2) Degree of significance.* To what degree do you consider that this competence contributes decisively to the climate of psychological safety in the healthcare institution?

1 Not important at all – 5 Very important

| **Knowledge. In my opinion, internships in my work environment provide trainees with the competency to…** | **Degree of** | **1** | **2** | **3** | **4** | **5** |
| --- | --- | --- | --- | --- | --- | --- |
| - 1. …understand that an open and direct expression of concerns about patient safety can prevent the occurrence of incidents that could cause harm to the patient. | Acquisition |  |  |  |  |  |
|  | Significance |  |  |  |  |  |
| - 1. …know how to communicate assertively a concern about patient safety to another healthcare professional (of the same level or higher) (what words to choose, how to start and finish the conversation, what tone of voice or gestures to use, etc.). | Acquisition |  |  |  |  |  |
|  | Significance |  |  |  |  |  |
| - 1. …distinguish between situations that could cause avoidable harm to the patient from those that do not represent a high risk for the patient safety. | Acquisition |  |  |  |  |  |
|  | Significance |  |  |  |  |  |
| - 1. …choose the best moment to communicate specific concerns about patient safety to another healthcare professional (of the same level or higher). | Acquisition |  |  |  |  |  |
|  | Significance |  |  |  |  |  |
| - 1. …know how to assertively warn another healthcare professional (of the same level or higher) of the risk of ignoring an important patient safety rule (words to choose, how to start and finish the conversation, what tone of voice or gestures to use, etc.). | Acquisition |  |  |  |  |  |
|  | Significance |  |  |  |  |  |
| - 1. …know how to deal constructively with the possible negative reaction of a healthcare professional (of the same level or higher) after having warned them that they were overlooking an important rule for patient safety. | Acquisition |  |  |  |  |  |
|  | Significance |  |  |  |  |  |
| - 1. …know how to express specific proposals that could improve the patient safety in the unit. | Acquisition |  |  |  |  |  |
|  | Significance |  |  |  |  |  |

**2. COMPETENCES. Attitudes**

Please, assess each competency (attitude) according to two criteria:

*(1) Degree of acquisition*. To what degree are trainees currently acquiring this competency through their clinical internships at your institution?

1 No acquisition at all – 5 Fully acquired

*(2) Degree of significance.* To what degree do you consider that this competence contributes decisively to the climate of psychological safety in the healthcare institution?

1 Not important at all – 5 Very important

| **Attitude. In my opinion, internships in my work environment provide trainees with the competency to:** | **Degree of** | **1** | **2** | **3** | **4** | **5** |
| --- | --- | --- | --- | --- | --- | --- |
| - 1. …commit to the identification and prevention of risks for patient safety. | Acquisition |  |  |  |  |  |
|  | Significance |  |  |  |  |  |
| - 1. …perceive risk situations in daily work as an opportunity to highlight the risk and take appropriate measures to prevent harm to patients. | Acquisition |  |  |  |  |  |
|  | Significance |  |  |  |  |  |
| - 1. …respond positively to the expression of warnings or concerns that other healthcare professionals (of the same level or higher) make in relation to patient safety. | Acquisition |  |  |  |  |  |
|  | Significance |  |  |  |  |  |
| - 1. …maintain a positive attitude towards warning other healthcare professionals if with their actions they are ignoring an important patient safety rule. | Acquisition |  |  |  |  |  |
|  | Significance |  |  |  |  |  |
| - 1. …be willing to openly and directly share specific proposals to improve patient safety. | Acquisition |  |  |  |  |  |
|  | Significance |  |  |  |  |  |
| - 1. …be willing to learn from mistakes and patient safety incidents in which other professionals have been involved, instead of judging them. | Acquisition |  |  |  |  |  |
|  | Significance |  |  |  |  |  |

**3. COMPETENCES. Skills**

Please, assess each competency (skill) according to two criteria:

*(1) Degree of acquisition*. To what degree are trainees currently acquiring this competency through their clinical internships at your institution?

1 No acquisition at all – 5 Fully acquired

*(2) Degree of significance.* To what degree do you consider that this competence contributes decisively to the climate of psychological safety in the healthcare institution?

1 Not important at all – 5 Very important

| **Skills. In my opinion, internships in my work environment provide trainees with the competency to:** | **Degree of** | **1** | **2** | **3** | **4** | **5** |
| --- | --- | --- | --- | --- | --- | --- |
| - 1. …communicate openly and directly to other professionals (of same level or higher) specific concerns about patient safety by presenting information, asking questions, or expressing opinions. | Acquisition |  |  |  |  |  |
|  | Significance |  |  |  |  |  |
| - 1. …request the responsible professionals’ advice to report, in the appropriate system, the occurrence of a patient safety incident that has been witnessed and make the report (if necessary). | Acquisition |  |  |  |  |  |
|  | Significance |  |  |  |  |  |
| - 1. …warn assertively to another healthcare professional (of the same level or higher) that, with their actions, they are ignoring an important patient safety rule. | Acquisition |  |  |  |  |  |
|  | Significance |  |  |  |  |  |
| - 1. …respond assertively to the negative reaction of a healthcare professional (of the same level or higher) whom they have warned of ignoring an important patient safety rule. | Acquisition |  |  |  |  |  |
|  | Significance |  |  |  |  |  |
| - 1. …verbally support and reinforce the initiative of other healthcare professionals (of the same level or higher) to share their specific concerns about patient safety with the rest of the team. | Acquisition |  |  |  |  |  |
|  | Significance |  |  |  |  |  |
| - 1. …set and communicate concrete proposals to improve patient safety in the own unit or service. | Acquisition |  |  |  |  |  |
|  | Significance |  |  |  |  |  |
| - 1. …offer peer support to a colleague involved in an adverse event to reduce the second victim syndrome (characterized by feelings of guilt, inadequacy, anxiety, shame, hypervigilance, or grief). | Acquisition |  |  |  |  |  |
|  | Significance |  |  |  |  |  |

**4. INTERVENTIONS**

Please, assess each intervention according to two criteria:

*(1) Degree of implementation*. To what degree is this intervention implemented in your immediate clinical setting?

1 Not yet implemented – 5 Fully implemented

*(2) Degree of significance*. To what degree do you consider that this intervention contributes decisively to the climate of psychological safety in the healthcare institution?

1 Not important at all – 5 Very important

| **Interventions. My healthcare institution…** | **Degree of** | **1** | **2** | **3** | **4** | **5** |
| --- | --- | --- | --- | --- | --- | --- |
| - 1. …implements a training program for new staff (especially trainees) to foster a positive patient safety culture and a psychological safety climate. | Implementation |  |  |  |  |  |
|  | Significance |  |  |  |  |  |
| - 1. …appoints an influential group of people to design an intervention plan to foster a trusting climate among healthcare professionals to ensure patient safety. | Implementation |  |  |  |  |  |
|  | Significance |  |  |  |  |  |
| - 1. …holds regular clinical sessions with trainees to share patient safety concerns and lessons learned. This measure translates into the set of shared spaces to exchange experiences on patient safety incidents, devise barriers to minimize risks, and provide emotional and instrumental support among peers. | Implementation |  |  |  |  |  |
|  | Significance |  |  |  |  |  |
| - 1. …raises awareness among the centre’s professionals, with the collaboration of heads of service, of the need to encourage trainees and colleagues to express their concerns regarding patient safety openly and directly and to warn other professionals of the risks they identify in their daily work. | Implementation |  |  |  |  |  |
|  | Significance |  |  |  |  |  |
| - 1. …raises awareness among the centre’s professionals, with the collaboration of heads of service, of the importance of responding positively to warnings from other professionals regarding compliance with relevant patient safety rules and reinforcing the open expression of specific patient safety concerns by trainees. | Implementation |  |  |  |  |  |
|  | Significance |  |  |  |  |  |
| - 1. …provides trainees with the opportunity to participate as observers during the planning of adverse event disclosure conversations with the affected patient and family. | Implementation |  |  |  |  |  |
|  | Significance |  |  |  |  |  |
| - 1. …allows trainees to have the opportunity to be present during the discussion and analysis following a patient safety incident. | Implementation |  |  |  |  |  |
|  | Significance |  |  |  |  |  |
| - 1. …provides trainees with specific training on reporting patient safety incidents by appropriate means. | Implementation |  |  |  |  |  |
|  | Significance |  |  |  |  |  |
| - 1. …offers institutional support to healthcare professionals involved in an adverse event to contribute to better safety at the workplace. | Implementation |  |  |  |  |  |
|  | Significance |  |  |  |  |  |

**5. IF YOU WISH, use this space to add something you missed in the questionnaire or anything that you consider appropriate to note regarding the topic addressed.**

|  |
| --- |

**6. TO FINISH, please indicate**

Country: Croatia / Estonia / Finland / Germany / Israel / Lithuania / Portugal / Slovakia / Spain / Serbia / Other

Age: _____________

Sex:  Man  Woman  Other

Professional profile:

Medicine

Nursing

Pharmacy

Midwifery

Physiotherapy

Psychology

Sociology

Other

Years being responsible of trainees: _________

Number of trainees that you have personally been supervising or mentoring in the last three years (2019-2021): _________

Is there a specific training program in patient safety at your centre?  Yes  No

Setting where you perform your clinical and mentoring work:

Primary care

Specialised care (hospital)

Social care

**CROATIAN VERSION**


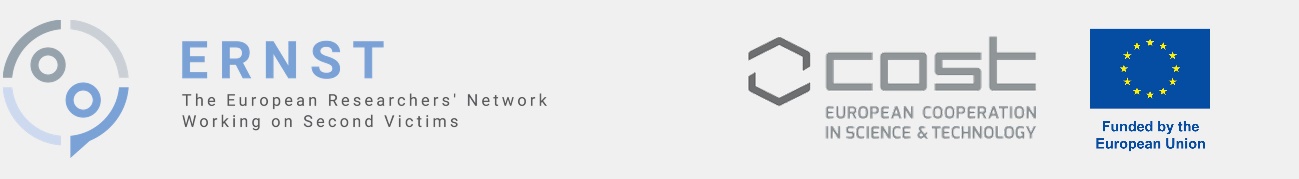


**Kompetencije pripravnika u zdravstvenoj djelatnosti za promicanje klime psihološke sigurnosti u kliničkim okruženjima**

Molim lijepo Vas koji radite kao mentori, da pristanete na poziv za sudjelovanje u međunarodnoj studiji o kompetencijama, koje su stekli pripravnici i studenti zdravstvenih studija tijekom školovanja, iz područja psihološke sigurnosti u kliničkom okruženju.

Ovo istraživanje provodi tim istraživača iz akademskog i kliničkog područja europskog ERNST konzorcija (<https://cost-ernst.eu/>), kojem je u središtu pozornosti sigurnost pacijenata i psihološka sigurnost zdravstvenih djelatnika. U ovoj studiji sudjeluju istraživači iz Hrvatske, Estonije, Finske, Njemačke, Izraela, Litve, Portugala, Slovačke, Španjolske i Srbija.

Psihološka sigurnost odnosi se na zajednički stav da se radni tim može suočiti sa složenim izazovima u okruženju međusobnog poštovanja i povjerenja. Zdravstveni djelatnici pružaju sigurniju skrb kada se o uzrocima i prevenciji kliničkih pogrešaka može raspravljati i analizirati bez straha od kritika ili sankcija. Psihološka sigurnosna klima može biti determinanta sigurnosti pacijenata.

Ova Delphy studija istražuje:

- koje kompetencije psihološke sigurnosti (znanja, stavove i vještine) stječu buduće generacije zdravstvenih djelatnika kroz svoje kliničko stažiranje i praksu (studenti, pripravnici i specijalizanti koji se u daljnjem tekstu nazivaju „pripravnici”)
- koje aktivnosti zdravstvene ustanove, u kojima pripravnici odrađuju klinički staž, provode za promicanje stjecanja ovih kompetencija.

Odgovori na upitnik analizirat će se na pseudo-anonimiziran i agregiran način. Ne postoje točni ili pogrešni odgovori na pitanja, radi se o vašoj percepciji učinkovitosti kliničke edukacije o sigurnosti pacijenata i sigurnosti djelatnika.

Za popunjavanje upitnika potrebno je otprilike 10 minuta. Molimo na kraju upitnika pošaljite odgovore.

Za bilo kakva pitanja ili komentare, pošaljite e-mail svom nacionalnom koordinatoru.

Unaprijed vam zahvaljujemo na suradnji i vašim odgovorima.

**Informirani pristanak**

Sudjelovanje u ovom studiju je dobrovoljno. Prikupljeni podaci bit će psudo-anonimni i analizirani u zbirnom obliku. Platforma je smještena na sigurnom poslužitelju koji se nalazi u Španjolskoj i kojim upravlja Sveučilište Miguel Hernández, dio španjolskog javnog obrazovnog sustava. Molimo potvrdite da sudjelujete u ovom istraživanju na dobrovoljnoj i informiranoj osnovi. Svoj pristanak za sudjelovanje u istraživanju možete opozvati u bilo kojem trenutku slanjem e-pošte na ___________

Molimo Vas da svaku kompetenciju (znanje) ocijenite prema dva kriterija:

**1. ZNANJE**

*(1)* *Stupanj stjecanja kompetencije*. U kojoj mjeri pripravnici trenutno stječu ovu kompetenciju kroz klinički staž u vašoj ustanovi?

1 Uopće nema stjecanja – 5 Potpuno stečeno

*(2) Stupanj važnosti*. U kojoj mjeri smatrate da ova kompetencija presudno doprinosi klimi psihološke sigurnosti u zdravstvenoj ustanovi?

1 Uopće nije važno – 5 Vrlo važno

| **ZNANJE. Po mom mišljenju, pripravnički staž u mom radnom okruženju daje pripravnicima sljedeća znanja:** | **Stupanj** | **1** | **2** | **3** | **4** | **5** |
| --- | --- | --- | --- | --- | --- | --- |
| - 1. … Otvoreno i izravno izražavanje brige za sigurnost pacijenata može spriječiti pojavu incidenata koji bi mogli naštetiti pacijentu. | Stjecanja |  |  |  |  |  |
|  | Važnosti |  |  |  |  |  |
| - 1. …Poznavanje dobrog načina komunikacije o sigurnosti pacijenata s drugim zdravstvenim djelatnikom (iste razine ili više) (npr. znati koje riječi odabrati, kako započeti i završiti razgovor, koji ton glasa ili geste koristiti, itd.). | Stjecanja |  |  |  |  |  |
|  | Važnosti |  |  |  |  |  |
| - 1. …Razlikovanje situacije koja bi pacijentu mogla prouzročiti štetu a može se izbjeći, od situacije koja ne predstavlja veliki rizik za sigurnost pacijenta. | Stjecanja |  |  |  |  |  |
|  | Važnosti |  |  |  |  |  |
| - 1. …Odabiranje najboljeg trenutka da drugom zdravstvenom djelatniku (iste razine ili više) prenesete specifične brige o sigurnosti pacijenata. | Stjecanja |  |  |  |  |  |
|  | Važnosti |  |  |  |  |  |
| - 1. …Znati na pravi način upozoriti drugog zdravstvenog djelatnika (iste razine ili više) na rizik zanemarivanja važnog pravila sigurnosti pacijenta (npr. riječi koje treba izabrati, kako započeti i završiti razgovor, koji ton glasa ili geste koristiti itd.) | Stjecanja |  |  |  |  |  |
|  | Važnosti |  |  |  |  |  |
| - 1. … Konstruktivno se nositi s mogućom negativnom reakcijom zdravstvenog djelatnika (iste ili više razine) nakon što su ga upozorili da zanemaruje važno pravilo za sigurnost pacijenata. | Stjecanja |  |  |  |  |  |
|  | Važnosti |  |  |  |  |  |
| - 1. …Iznošenje konkretnih prijedloga koji bi mogli poboljšati sigurnost pacijenata na odjelu | Stjecanja |  |  |  |  |  |
|  | Važnosti |  |  |  |  |  |

**2. STAVOVI**

*(1) Stupanj stjecanja kompetencije*. U kojoj mjeri pripravnici trenutno stječu ovu kompetenciju kroz klinički staž u vašoj ustanovi?

1 Uopće nema stjecanja – 5 Potpuno stečeno

*(2) Stupanj važnosti.* U kojoj mjeri smatrate da ova kompetencija presudno doprinosi klimi psihološke sigurnosti u zdravstvenoj ustanovi?

1 Uopće nije važno – 5 Vrlo važno

| **STAVOVI. Po mom mišljenju, pripravnički staž u mom radnom okruženju omogućava pripravnicima da izgrade sljedeće stavove:** | **Stupanj** | **1** | **2** | **3** | **4** | **5** |
| --- | --- | --- | --- | --- | --- | --- |
| - 1. … Identifikacija i prevencija rizika za sigurnost pacijenata je obaveza. | Stjecanja |  |  |  |  |  |
|  | Važnosti |  |  |  |  |  |
| - 1. …Prepoznavanje rizične situacije u svakodnevnom radu je prilika za poduzimanje odgovarajućih mjera prevencije nastanka štete pacijentu. | Stjecanja |  |  |  |  |  |
|  | Važnosti |  |  |  |  |  |
| - 1. … Treba reagirati pozitivno na upozoravanje ili zabrinutost drugih zdravstvenih djelatnika (iste ili više razine) vezano uz sigurnost pacijenata. | Stjecanja |  |  |  |  |  |
|  | Važnosti |  |  |  |  |  |
| - 1. … Treba upozoriti druge zdravstvene djelatnike, koji svojim postupcima zanemaruju važno pravilo sigurnosti pacijenata, na način da se održi pozitivan stav. | Stjecanja |  |  |  |  |  |
|  | Važnosti |  |  |  |  |  |
| - 1. …Treba biti spreman otvoreno i izravno davati konkretne prijedloga za poboljšanje sigurnosti pacijenata. | Stjecanja |  |  |  |  |  |
|  | Važnosti |  |  |  |  |  |
| - 1. …Treba biti spreman učiti iz pogrešaka i sigurnosnih incidenata u koje su bili uključeni drugi profesionalci, umjesto da ih se osuđuje. | Stjecanja |  |  |  |  |  |
|  | Važnosti |  |  |  |  |  |

**3. VJEŠTINE**

*(1) Stupanj stjecanja kompetencije*. U kojoj mjeri pripravnici trenutno stječu ovu kompetenciju kroz klinički staž u vašoj ustanovi?

1 Uopće nema stjecanja – 5 Potpuno stečeno

*(2) Stupanj važnosti*. U kojoj mjeri smatrate da ova kompetencija presudno doprinosi klimi psihološke sigurnosti u zdravstvenoj ustanovi?

1 Uopće nije važno – 5 Vrlo važno

| **VJEŠTINE. Po mom mišljenju, pripravnički staž u mom radnom okruženju daje pripravnicima sljedeće vještine:** | **Degree of** | **1** | **2** | **3** | **4** | **5** |
| --- | --- | --- | --- | --- | --- | --- |
| - 1. ... Vještinu otvorenog i izravnog komuniciranja s drugim stručnjacima (iste razine ili više) o specifičnim problemima veznim uz sigurnost pacijenata i to pružanjem informacija, postavljanjem pitanja ili izražavanjem mišljenja. | Stjecanja |  |  |  |  |  |
|  | Važnosti |  |  |  |  |  |
| - 1. …Traženje savjeta odgovornog stručnjaka za prijavu incidenta koji ugrožava sigurnost pacijenata, kojem ste svjedočili, i izrada prijave ili izvještaja u odgovarajućem sustavu. | Stjecanja |  |  |  |  |  |
|  | Važnosti |  |  |  |  |  |
| - 1. …Moći odlučno upozoriti drugog zdravstvenog djelatnika (iste ili više razine) da svojim postupcima zanemaruje važno pravilo sigurnosti pacijenata. | Stjecanja |  |  |  |  |  |
|  | Važnosti |  |  |  |  |  |
| - 1. …Imati komunikacijsku vještinu odgovaranja na negativnu reakciju zdravstvenog djelatnika (iste razine ili više) kojeg ste upozorili da ignorira važno pravilo sigurnosti pacijenata. | Stjecanja |  |  |  |  |  |
|  | Važnosti |  |  |  |  |  |
| - 1. ...Usmeno poduprijeti i osnažiti inicijativu drugih zdravstvenih djelatnika (iste razine ili više) da podijele svoje specifične brige o sigurnosti pacijenata s ostatkom tima. | Stjecanja |  |  |  |  |  |
|  | Važnosti |  |  |  |  |  |
| - 1. …Moći postaviti i priopćiti konkretne prijedloge za poboljšanje sigurnosti pacijenata u vlastitom odjelu ili službi. | Stjecanja |  |  |  |  |  |
|  | Važnosti |  |  |  |  |  |
| - 1. ... Ponuditi vršnjačku podršku kolegi koji je uključen u štetni događaj kako biste smanjili sindrom sekundarne žrtve (karakteriziran osjećajem krivnje, neadekvatnosti, tjeskobe, srama, hiperaktivnosti ili tuge). | Stjecanja |  |  |  |  |  |
|  | Važnosti |  |  |  |  |  |

**4. INTERVENCIJE**

Molimo Vas da svaku intervenciju ocijenite prema dva kriterija:

*(1) Stupanj provedbe (implementacije)*. U kojoj se mjeri ova intervencija provodi u vašem neposrednom kliničkom okruženju?

1 Još nije implementirano – 5 Potpuno provedeno

*(2) Stupanj važnosti*. U kojoj mjeri smatrate da ova intervencija presudno doprinosi klimi psihičke sigurnosti u zdravstvenoj ustanovi?

1 Uopće nije važno – 5 Vrlo važno

| **INTERVENCIJE. Moja zdravstvena ustanova…** | **Stupanj** | **1** | **2** | **3** | **4** | **5** |
| --- | --- | --- | --- | --- | --- | --- |
| - 1. …provodi program edukacije za novo osoblje (osobito pripravnike) kako bi potaknula pozitivnu kulturu sigurnosti pacijenata i klimu psihološke sigurnosti. | Implementacije |  |  |  |  |  |
|  | Važnosti |  |  |  |  |  |
| - 1. ...imenuje radnu skupinu djelatnika za izradu plana intervencije koja potiče klimu povjerenja među zdravstvenim djelatnicima kako bi se osigurala sigurnost pacijenata. | Implementacije |  |  |  |  |  |
|  | Važnosti |  |  |  |  |  |
| - 1. ..održava redovite kliničke sastanke sa pripravnicima kako bi podijelili važnost brige za sigurnost pacijenata i naučene lekcije. Ova mjera se pretvara u prostor za razmjenu iskustava o incidentima vezanim za sigurnost pacijenata, osmišljavanje preventivnih mjera za smanjenje rizika te pružanje emocionalne i institucionalne podrške među kolegama. | Implementacije |  |  |  |  |  |
|  | Važnosti |  |  |  |  |  |
| - 1. …podiže svijesnost stručnjaka ustanove, uz suradnju voditelja službi, o potrebi poticanja pripravnika i kolega da otvoreno i izravno izraze svoju zabrinutost u vezi sa sigurnošću pacijenata kao i potrebi upozoravanja ostalih stručnjaka da u svakodnevnom radu identificiraju rizike. | Implementacije |  |  |  |  |  |
|  | Važnosti |  |  |  |  |  |
| - 1. …podiže svijesnost stručnjaka ustanove, uz suradnju voditelja službi, o važnosti pozitivnog reagiranja na upozorenja ostalih stručnjaka u vezi s pridržavanjem propisa za sigurnost pacijenata i važnosti jačanja otvorenog izražavanja brige za sigurnost pacijenata od strane pripravnika. | Implementacije |  |  |  |  |  |
|  | Važnosti |  |  |  |  |  |
| - 1. …pruža pripravnicima priliku da sudjeluju kao promatrači tijekom planiranja razgovora o otkrivanju neželjenog događaja oboljeliom pacijentu i obitelji. | Implementacije |  |  |  |  |  |
|  | Važnosti |  |  |  |  |  |
| - 1. …omogućuje pripravnicima da mogu biti prisutni tijekom rasprave i analize nakon incidenta vezanog uz sigurnost pacijenta. | Implementacije |  |  |  |  |  |
|  | Važnosti |  |  |  |  |  |
| - 1. …omogućuje pripravnicima posebnu edukaciju o prijavljivanju incidenata vezanom uz sigurnost pacijenata na prikladan način. | Implementacije |  |  |  |  |  |
|  | Važnosti |  |  |  |  |  |
| - 1. … nudi institucionalnu podršku zdravstvenim djelatnicima koji su uključeni u neželjeni događaj kako bi pridonijeli boljoj sigurnosti na radnom mjestu. | Implementacije |  |  |  |  |  |
|  | Važnosti |  |  |  |  |  |

**5. AKO ŽELITE, koristite ovaj prostor da dodate nešto što je propušteno u upitniku ili bilo što što smatrate prikladnim zabilježiti u vezi s obrađenom temom.**

|  |
| --- |

**6. ZA KRAJ, molimo Vas označite:**

Državu: Hrvatska / Estonija / Finska / Njemačka / Izrael / Litva / Portugal / Slovačka / Španjolska / Srbija / Ostalo

Dob: _____________

Spol: ☐ Muškarac ☐ Žena ☐ Ostalo

Vaše profesionalno područje:

☐ Medicina

☐ Sestrinstvo

☐ Farmacija

☐ Primaljstvo

☐ Fizioterapija

☐ Psihologija

☐ Sociologija

☐ Ostalo

Godine rada s pripravnicima: _________

Broj pripravnika koje ste osobno nadzirali ili mentorirali u posljednje tri godine (2019.-2021.): _________

Postoji li u vašoj ustanovi poseban program edukacije o sigurnosti pacijenata? ☐ Da ☐ Ne

Gdje obavljate svoj klinički i mentorski rad:

☐ Primarna skrb

☐ Specijalizirana njega (bolnica)

☐ Socijalna skrb

**ESTONIAN VERSION**


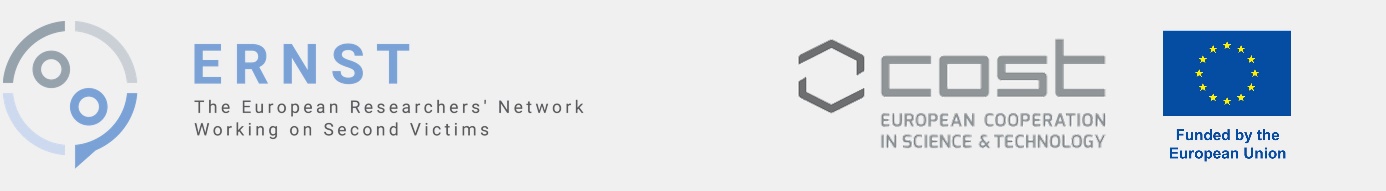


**Psühholoogilise turvalisuse pädevuste omandamine residentuuri ja kliinilise praktika jooksul ning võimalikud sekkumised pädevuste parandamiseks residentuuri ja praktika juhendajate vaatekohast**

Täname, et nõustusite osalema selles uuringus, mis käsitleb residentide ja tervisevaldkonna üliõpilaste residentuuri ja kliinilise praktika õppeprotsessi jooksul omandatud psühholoogilise turvalisuse pädevusi ning sekkumisi pädevuste parandamiseks.

Seda uuringut korraldavad patsientide ohutusele ja teisese kannatanu ehk teisese ohvri (tervishoiutöötaja, kes on otseselt seotud patsiendile tervisekahju või patsiendi surma põhjustanud juhtumiga ning kellest on saanud läbielatud sündmuse tõttu emotsionaalse trauma ohver) uuringutele keskendunud akadeemilise ja kliinilise valdkonna teadlaste rühm Euroopa ERNST (The European Researchers' Network Working on Second Victims) konsortsiumist. Uuring korraldatakse Horvaatias, Eestis, Soomes, Saksamaal, Iisraelis, Leedus, Portugalis, Slovakkias, Hispaanias ja Serbia.

Psühholoogiline turvalisus tähendab kindlustunnet, et organisatsioonis saavad meeskonnaliikmed omavahel avatult rääkida: küsida küsimusi, avaldada arvamust ning rääkida vigadest, kartmata piinlikkust, naeruvääristamist või karistamist. Psühholoogiline turvalisus eksisteerib, kui inimesed tunnevad end vabalt ja nad tajuvad, et kui nad eksivad, ei karistata ega mõisteta neid hukka, ei arvata neist halvasti; ei halvustata ega karistata abi, informatsiooni või tagasiside küsimisel ning arvamuse avaldamisel. Psühholoogilise turvalisuse eksisteerimine on patsiendi ohutuse tagamisel määravaks teguriks, kuna see võimaldab analüüsida vigade võimalikke põhjusi ja seeläbi ennetada kliinilise tegevusega seotud kahjujuhtumeid (juhtum, mille tagajärjeks on kahju tekitamine patsiendile).

Selles Delphi uuringus selgitatakse:

- milliseid psühholoogilise turvalisuse pädevusi (teadmised, oskused ja hoiakud) omandavad residentuuri ja praktikajuhendajate hinnangul arst-residendid ja tervisevaldkonna (õe, ämmaemanda ja bioanalüütiku eriala) üliõpilased kliinilise praktika käigus ning
- milliseid sekkumisi peaksid residentuuri- ja praktikabaasideks olevad tervishoiuasutused rakendama nende pädevuste omandamise parandamiseks.

Teie vastuseid analüüsitakse igas küsitlusvoorus ja küsitlusvoorude järgselt anonüümselt, kasutades selleks topeltpimedat ühesuunalist kodeerimist. Küsimustele ei ole õigeid ega valesid vastuseid, uuringu eesmärgiks on selgitada Teie kui residentuuri või praktikajuhendaja arvamust. Küsimustiku täitmine võtab aega umbes 15 minutit. Ärge unustage küsimustiku lõpus esitada oma vastuseid.

Küsimuste või kommentaaride korral võtke palun ühendust uuringu Eesti koordinaatoritega, kes kutsusid Teid uuringus osalema või saatke e-kiri aadressile ____________

Täname Teid koostöö eest.

**1. PÄDEVUSED. Teadmised**

Palun hinnake iga pädevust (teadmisi) kahe kriteeriumi alusel:

*(1) Omandamine:* Mil määral omandavad praktikandid või residendid praegu seda pädevust teie asutuses kliinilise praktika või residentuuri käigus?

1 Ei omandata üldse – 5 Omandavad täielikult

*(2) Tähtsus:* Mil määral aitab see pädevus teie arvates otsustavalt kaasa psühholoogiliselt turvalisele õhkkonnale tervishoiuasutuses?

1 Ei ole üldse tähtis – 5 Väga tähtis

| **Teadmised. Minu arvates annab praktika minu töökeskkonnas praktikantidele või residentidele pädevuse...:** | **Omandamise ja tähtsuse määr** | **1** | **2** | **3** | **4** | **5** |
| --- | --- | --- | --- | --- | --- | --- |
| - 1. …mõista, et patsiendi ohutusega seotud mure avalik ja otsene väljendamine võib ära hoida juhtumeid, mis võivad patsienti kahjustada. | Omandamine |  |  |  |  |  |
|  | Tähtsus |  |  |  |  |  |
| - 1. …kuidas väljendada enesekindlalt patsiendi ohutusega seotud muret teisele (samal või kõrgemal ametikohal töötavale) tervishoiutöötajale (missuguseid sõnu valida, kuidas vestlust alustada ja lõpetada, millist hääletooni või žeste kasutada jne). | Omandamine |  |  |  |  |  |
|  | Tähtsus |  |  |  |  |  |
| - 1. …eristada patsiendile välditavat kahju tekitavaid olukordi nendest, mis ei kujuta endast suurt riski patsiendi ohutusele. | Omandamine |  |  |  |  |  |
|  | Tähtsus |  |  |  |  |  |
| - 1. …valida parim hetk, et teavitada konkreetsest patsiendi ohutusega seotud murest teist (samal või kõrgemal ametikohal töötavat) tervishoiutöötajat. | Omandamine |  |  |  |  |  |
|  | Tähtsus |  |  |  |  |  |
| - 1. …enesekindlalt hoiatada teist (samal või kõrgemal ametikohal töötavat) tervishoiutöötajat olulise patsiendiohutuse reegli eiramise ohu eest (sõnad, mida valida, kuidas vestlust alustada ja lõpetada, millist hääletooni või žeste kasutada jne). | Omandamine |  |  |  |  |  |
|  | Tähtsus |  |  |  |  |  |
| - 1. …kuidas konstruktiivselt käsitleda samal või kõrgemal ametikohal töötava tervishoiutöötaja võimalikku negatiivset reaktsiooni pärast seda, kui ta on hoiatanud patsiendiohutuse seisukohalt olulise reegli eiramise eest. | Omandamine |  |  |  |  |  |
|  | Tähtsus |  |  |  |  |  |
| - 1. …kuidas esitada konkreetseid ettepanekuid, mis võiksid osakonnas patsiendi ohutust parandada. | Omandamine |  |  |  |  |  |
|  | Tähtsus |  |  |  |  |  |

**2. Pädevused. Hoiakud**

Palun hinnake iga pädevust (hoiakut) kahe kriteeriumi alusel:

*(1) Omandamine:* Mil määral omandavad praktikandid või residendid praegu seda pädevust teie asutuses kliinilise praktika käigus?

1 Ei omandata üldse – 5 Omandavad täielikult

*(2) Tähtsus:* Mil määral aitab see pädevus teie arvates otsustavalt kaasa psühholoogiliselt turvalisele õhkkonnale tervishoiuasutuses?

1 Ei ole üldse tähtis – 5 Väga tähtis

| **Hoiakud. Minu arvates annab praktika minu töökeskkonnas praktikantidele või residentidele pädevuse...:** | **Omandamise ja tähtsuse määr** | **1** | **2** | **3** | **4** | **5** |
| --- | --- | --- | --- | --- | --- | --- |
| - 1. …tuvastada ja ennetada patsiendi ohutusega seotud riske. | Omandamine |  |  |  |  |  |
|  | Tähtsus |  |  |  |  |  |
| - 1. …näha ja tajuda riskiolukordi kui võimalust riskolukordade esiletõstmiseks ja patsiendi kahjustamise ennetamiseks asjakohaste abinõude rakendamise kaudu. | Omandamine |  |  |  |  |  |
|  | Tähtsus |  |  |  |  |  |
| - 1. …reageerida positiivselt samal või kõrgemal positsioonil töötavate kolleegide väljendatud murele või tähelepanekutele seoses patsiendiohutusega. | Omandamine |  |  |  |  |  |
|  | Tähtsus |  |  |  |  |  |
| - 1. …säilitada positiivne hoiak, juhtides teiste tervishoiutöötajate tähelepanu olukordadele, kui nende tegevusega kaasneb patsiendi ohutust tagavate reeglite eiramine. | Omandamine |  |  |  |  |  |
|  | Tähtsus |  |  |  |  |  |
| - 1. …olla valmis avameelselt ja vahetult jagama konkreetseid ettepanekuid patsiendi ohutuse parandamiseks. | Omandamine |  |  |  |  |  |
|  | Tähtsus |  |  |  |  |  |
| - 1. …olla valmis õppima kolleegide vigadest ja patsiendiohutust mõjutavatest juhtumitest, selle asemel, et nende tegevust arvustada või hukka mõista. | Omandamine |  |  |  |  |  |
|  | Tähtsus |  |  |  |  |  |

**3. Pädevused. Oskused**

Palun hinnake iga pädevust (oskust) kahe kriteeriumi alusel:

*(1) Omandamine:* Mil määral omandavad praktikandid või residendid praegu seda pädevust teie asutuses kliinilise praktika käigus?

1 Ei omandata üldse – 5 omandavad täielikult

*(2) Tähtsus:* Mil määral aitab see pädevus teie arvates otsustavalt kaasa psühholoogiliselt turvalisele õhkkonnale tervishoiuasutuses?

1 Ei ole üldse tähtis – 5 Väga tähtis

| **Oskused. Minu arvates annab praktika minu töökeskkonnas praktikantidele ja residentidele pädevuse...:** | **Omandamise ja tähtsuse määr** | **1** | **2** | **3** | **4** | **5** |
| --- | --- | --- | --- | --- | --- | --- |
| - 1. …rääkida avatult ja vahetult teiste samal või kõrgemal ametikohal töötavate kolleegidega konkreetsetest patsientide ohutusega seotud muredest, edastada sellekohast teavet, esitada küsimusi või avaldada arvamust | Omandamine |  |  |  |  |  |
|  | Tähtsus |  |  |  |  |  |
| - 1. …paluda vastutavalt töötajalt abi patsiendiohutusega seotud juhtumist teavitamisel, mille tunnistajaks praktikant või resident on olnud, ning vajadusel juhtumiteavitussüsteemi aruande koostamisel. | Omandamine |  |  |  |  |  |
|  | Tähtsus |  |  |  |  |  |
| - 1. …juhtida (samal või kõrgemal ametikohal töötava) kolleegi tähelepanu asjaolule, et ta eirab oma tegevusega olulist patsiendiohutusega seotud reeglit. | Omandamine |  |  |  |  |  |
|  | Tähtsus |  |  |  |  |  |
| - 1. ...reageerida otsustavalt ja enesekindlalt samal või kõrgemal ametikohal töötava kolleegi negatiivsele reaktsioonile, mis järgneb tähelepanu juhtimisele seoses patsiendiohutuse reegli eiramisega. | Omandamine |  |  |  |  |  |
|  | Tähtsus |  |  |  |  |  |
| - 1. …toetada verbaalselt ja võimendada samal või kõrgemal positsioonil töötavate kolleegide algatust jagada oma konkreetseid patsiendiohutusega seotud muresid teiste meeskonnaliikmetega. | Omandamine |  |  |  |  |  |
|  | Tähtsus |  |  |  |  |  |
| - 1. …koostada ja edastada konkreetseid ettepanekuid patsiendi ohutuse parandamiseks oma üksuses või teenistuses | Omandamine |  |  |  |  |  |
|  | Tähtsus |  |  |  |  |  |
| - 1. …pakkuda abi ja toetust kolleegile, kes on seotud patsiendile kahju põhjustanud juhtumiga, et vähendada teisese kannatanu sündroomiga kaasnevaid ilminguid (süütunne, alaväärsustunne, ärevus, häbi, ülivalvsus või lein). | Omandamine |  |  |  |  |  |
|  | Tähtsus |  |  |  |  |  |

**4. SEKKUMISED**

Palun hinnake iga sekkumist kahe kriteeriumi alusel:

*(1) Rakendatuse aste*. Mil määral seda sekkumist rakendatakse Teie töökeskkonnas?

1 Ei ole veel rakendatud – 5 Täielikult rakendatud

*(2) Tähtsuse aste*. Mil määral aitab see sekkumine Teie arvates kaasa psühholoogilise turvalisuse tagamisele tervishoiuasutuses?

1 Ei ole üldse tähtis – 5 Väga tähtis

| **Sekkumised. Tervishoiuasutuses, kus ma töötan…** | **Rakendamise ja tähtsuse määr** | **1** | **2** | **3** | **4** | **5** |
| --- | --- | --- | --- | --- | --- | --- |
| - 1. …on uutele töötajatele (eeskätt praktikantidele ja residentidele) koolitusprogramm, et edendada positiivset patsiendiohutuskultuuri ja psühholoogilise turvalisuse õhkkonda. | Rakendamine |  |  |  |  |  |
|  | Tähtsus |  |  |  |  |  |
| - 1. ...on olemas töörühm, kelle ülesandeks on koostada sekkumiskava, mille abil edendada töötajate hulgas usalduslikku õhkkonda ning seeläbi tagada patsiendiohutus. | Rakendamine |  |  |  |  |  |
|  | Tähtsus |  |  |  |  |  |
| - 1. …korraldatakse praktikantide või residentidega regulaarseid kohtumisi, et rääkida patsiendiohutusega seotud probleemidest ning jagada vigadest õppimise kogemust. See tähendab kogemuste vahetamist patsiendiohutust mõjutavate juhtumite kohta, abinõude kavandamist riskide minimeerimiseks ning emotsionaalse toe ja abi pakkumist kolleegide poolt. | Rakendamine |  |  |  |  |  |
|  | Tähtsus |  |  |  |  |  |
| - 1. …suurendatakse koostöös üksuste juhtidega töötajate teadlikkust vajadusest julgustada nii kolleege kui ka praktikante või residente rääkima avameelselt patsiendiohutusega seotud muredest ja vajadusest juhtida kolleegide tähelepanu patsiendiohutusega seotud probleemidele, mida nad oma igapäevatöös märkavad. | Rakendamine |  |  |  |  |  |
|  | Tähtsus |  |  |  |  |  |
| - 1. …suurendatakse koostöös üksuste juhtidega töötajate teadlikkust sellest, kui oluline on positiivselt reageerida sellele, kui kolleeg juhib tähelepanu patsiendiohutusega seotud reeglite jälgimisele ning julgustada ka praktikante või residente avaldama oma arvamust patsiendiohutuse küsimustes. | Rakendamine |  |  |  |  |  |
|  | Tähtsus |  |  |  |  |  |
| - 1. …võimaldatakse praktikantidel või residentidel osaleda vaatlejana protsessis, mille käigus valmistatakse ette avameelset vestlust kahjujuhtumi tõttu kannatanud patsiendi ja tema pereliikmetega. | Rakendamine |  |  |  |  |  |
|  | Tähtsus |  |  |  |  |  |
| - 1. …võimaldatakse praktikantidel või residentidel viibida patsiendiohutusjuhtumile järgnevas arutelus ja juhtumi analüüsis. | Rakendamine |  |  |  |  |  |
|  | Tähtsus |  |  |  |  |  |
| - 1. …pakutakse praktikantidele või residentidele koolitust, kuidas kasutada tervishoiuasutuse patsiendiohutusjuhtumite teavitussüsteemi. | Rakendamine |  |  |  |  |  |
|  | Tähtsus |  |  |  |  |  |
| - 1. …pakutakse ohutuma töökeskkonna saavutamise eesmärgil tuge tervishoiutöötajatele, kes on seotud patsiendile kahju põhjustanud juhtumiga. | Rakendamine |  |  |  |  |  |
|  | Tähtsus |  |  |  |  |  |

1. **KUI SOOVITE, lisage palun omapoolseid ettepanekuid või kommentaare, mida peate seoses käsitletud teemaga asjakohaseks.**

|  |
| --- |

**6. Vastaja taustaandmed**

Riik: Horvaatia / Eesti / Soome / Saksamaa / Iisrael / Leedu / Portugal / Slovakkia / Hispaania / Serbia / Muu

Vanus: _____________

Sugu:  Mees  Naine  Muu

Eriala:

Meditsiin (arst)

Õendus

Apteek

Ämmaemandus

Füsioteraapia

Psühholoogia

Sotsioloogia

Muu

Praktika/ residentide juhendamise aeg (aastates): _________

Praktikantide/ residentide arv, keda olete juhendanud viimase kolme aasta jooksul (2019-2022): _________

Kas Teie organisatsioonis/ üksuses on olemas patsiendiohutuse koolitusprogramm?  Jah  Ei

Teie töökoht (kliiniline töö ja juhendamine):

Esmatasand/ perearstikeskus

Haigla/ eriarstiabi osutav asutus

**FINNISH VERSION**


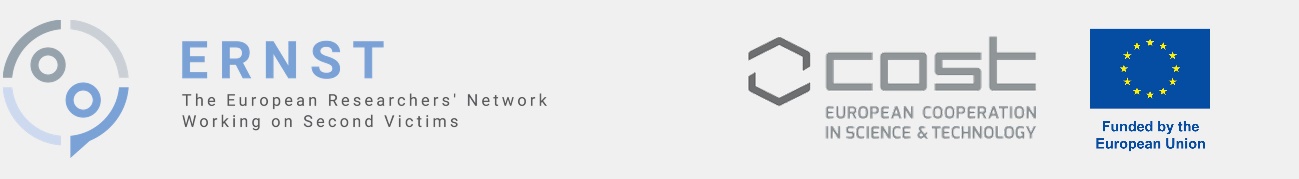


**Sosiaali- ja terveysalan opiskelijoiden ja vastavalmistuneiden oppiminen työharjoittelussa psykologisen turvallisuuden ilmapiirin edistämiseksi**

Kiitos, että suostuit osallistumaan tähän tutkimukseen, joka koskee sosiaali- ja terveysalan (sote) opiskelijoiden ja vastavalmistuneiden työharjoittelussa/työssään oppimiaan psykologiseen turvallisuuteen liittyviä taitoja.

Tutkijaryhmämme akateemisilta ja kliinisiltä aloilta on osa eurooppalaista ERNST-konsortiota, joka on keskittynyt potilasturvallisuuden ja second victim-ilmiön tutkimiseen. Tutkimusryhmään kuuluu tutkijoita Kroatista, Virosta, Suomesta, Saksasta, Israelista, Liettuasta, Portugalista, Slovakista, Espanjasta ja Serbia.

Psykologinen turvallisuus viittaa siihen, että työryhmä pystyy kohtaamaan monimutkaisia haasteita, kun työryhmässä vallitsee keskinäisen arvostuksen ja luottamuksen ilmapiiri. Tällaisessa ilmapiirissa sote-ammattilaiset voivat keskustella hoitoon liittyvistä virheistä, analysoida niihin johtaneita syitä sekä pohtia keinoja niiden välttämiseksi avoimesti ilman, että heidän tarvitsee pelätä arvostelua ja seuraamuksia. Psykologisesti turvallinen ilmapiiri voi olla määräävä tekijä potilasturvallisuudessa ja edistää turvallisempaa hoitoa.

Tässä Delphi-tutkimuksessa tutkitaan:

- mitä psykologista turvallisuusosaamista (tietoa, taitoja ja asenteita) tulevaisuuden sote-ammattilaiset (opiskelijat ja vastavalmistuneet, jäljempänä "tulevat sote-ammattilaiset") oppivat työharjoittelunsa kautta,
- mitä toimia sote-organisaatioiden olisi tehtävä edistääkseen tulevien sote-ammattilaisten psykologisen turvallisuusosaamisen oppimista.

Vastaukset anonymisoidaan ennen tulosten analysointia. Kysymyksiin ei ole oikeita tai vääriä vastauksia, vaan tarkoitus on kerätä tietoa siitä, miten ohjaajana arvioit tulevien sote-ammattilaisten psykologisen turvallisuuden oppimisen. Kyselylomakkeen täyttäminen kestää noin 10 minuuttia. Muistathan lähettää vastauksesi kyselylomakkeen loputtua.

Jos sinulla on kysyttävää tai kommentteja, lähetä sähköpostia kansalliselle koordinaattorillesi.

Kiitämme jo etukäteen, että vastasit tutkimuskyselyymme.

**Tietoon perustuva suostumus**

Tutkimukseen osallistuminen on vapaaehtoista. Kerätyt tiedot anonymisoidaan ja analysoidaan koosteena. Alustaa isännöidään Espanjassa sijaitsevalla suojatulla palvelimella ja sitä hallinnoi Miguel Hernándezin yliopisto, joka on osa Espanjan julkista koulutusjärjestelmää. Vahvista, että osallistut tähän tutkimukseen vapaaehtoisesti ja tietoisesti. Voit peruuttaa suostumuksesi osallistua tutkimukseen milloin tahansa lähettämällä sähköpostia ___________

Suostun osallistumaan tutkimukseen vapaaehtoisesti ja tietoon perustuen.

**1. OSAAMINEN. Tiedot**

Arvioi osaamisen (tiedot) väitteet kahden kriteerin mukaisesti:

*(1) Oppimisen aste*. Missä määrin tulevat sote-ammattilaiset oppivat tällä hetkellä nämä tiedot työharjoittelunsa kautta organisaatiossasi?

1 Ei oppimista lainkaan – 5 Oppii täysin

*(2) Merkityksellisyyden aste.* Miten tärkeäksi arvioit sen, että psykologisen turvallisuuden ilmapiiriä voidaan edistää omassa organisaatiossasi, kun tulevat sote-ammattilaiset oppivat nämä tiedot?

1 Ei ollenkaan tärkeää – 5 Erittäin tärkeää

| **Tiedot. Mielestäni harjoittelujaksot työpaikassani tarjoavat tuleville sote-ammattilaisille mahdollisuuden…** | **Asteikko** | **1** | **2** | **3** | **4** | **5** |
| --- | --- | --- | --- | --- | --- | --- |
| - 1. …ymmärtää, että potilasturvallisuutta koskevien huolenaiheiden avoin ja suora ilmaiseminen voi estää sellaisten vaaratilanteiden esiintymisen, jotka voivat aiheuttaa haittaa potilaalle. | Oppiminen |  |  |  |  |  |
|  | Merkityksellisyys |  |  |  |  |  |
| - 1. …tietää kuinka kertoa vakuuttavasti potilasturvallisuutta koskevasta huolesta sote-ammattilaiselle (sama taso tai korkeampi) (mitä sanoja valita, miten aloittaa ja lopettaa keskustelu, mitä äänensävyä tai eleitä käyttää jne.). | Oppiminen |  |  |  |  |  |
|  | Merkityksellisyys |  |  |  |  |  |
| - 1. …erottaa toisistaan tilanteet, jotka voivat aiheuttaa vältettävissä olevaa haittaa potilaalle, niistä tilanteista, jotka eivät aiheuta suurta riskiä potilasturvallisuudelle. | Oppiminen |  |  |  |  |  |
|  | Merkityksellisyys |  |  |  |  |  |
| - 1. …valita paras hetki ilmoittaa potilasturvallisuuteen liittyvistä erityisistä huolenaiheista sote- ammattilaiselle (samalle tai korkeammalle tasolle). | Oppiminen |  |  |  |  |  |
|  | Merkityksellisyys |  |  |  |  |  |
| - 1. …osata vakuuttavasti varoittaa toista sote-ammattilaista (samalla tasolla tai korkeammalla tasolla) riskistä jättää tärkeä potilasturvallisuusohje huomiotta (mitä sanoja valita, miten aloittaa ja lopettaa keskustelu, mitä äänensävyä tai eleitä käyttää jne.). | Oppiminen |  |  |  |  |  |
|  | Merkityksellisyys |  |  |  |  |  |
| - 1. …osata käsitellä rakentavasti (samantasoisen tai korkeamman) toisen sote-ammattilaisen mahdollista negatiivista reaktiota sen jälkeen, kun on varoittanut heitä siitä, että he jättävät huomiotta tärkeän potilasturvallisuusohjeen. | Oppiminen |  |  |  |  |  |
|  | Merkityksellisyys |  |  |  |  |  |
| - 1. …osata esittää erityisiä ehdotuksia, jotka voisivat parantaa potilasturvallisuutta yksikössä. | Oppiminen |  |  |  |  |  |
|  | Merkityksellisyys |  |  |  |  |  |

**2. OSAAMINEN. Asenteet**

Arvioi osaamista (asenteet) seuraavien kahden kriteerin mukaan:

*(1) Oppimisen aste*. Missä määrin tulevat sote-ammattilaiset oppivat tällä hetkellä nämä asenteet työharjoittelunsa kautta omassa organisaatiossasi?

1 Ei oppimista lainkaan – 5 Oppii täysin

*(2) Merkityksellisyyden aste.* Miten tärkeäksi arvioit sen, että psykologisen turvallisuuden ilmapiiriä voidaan edistää omassa organisaatiossasi, kun tulevat sote-ammattilaiset oppivat nämä asenteet?

1 Ei lainkaan tärkeää – 5 Erittäin tärkeää

| **Asenteet. Mielestäni harjoittelujaksot työpaikassani tarjoavat tuleville sote-ammattilaisille mahdollisuuden…** | **Asteikko** | **1** | **2** | **3** | **4** | **5** |
| --- | --- | --- | --- | --- | --- | --- |
| - 1. …sitoutua tunnistamaan ja ehkäisemään potilasturvallisuuteen liittyviä riskejä. | Oppiminen |  |  |  |  |  |
|  | Merkityksellisyys |  |  |  |  |  |
| - 1. …nähdä riskitilanteet päivittäisessä työssä mahdollisuutena tuoda esiin riskiä ja ryhtyä asianmukaisiin toimenpiteisiin potilaille aiheutuvien haittojen ehkäisemiseksi. | Oppiminen |  |  |  |  |  |
|  | Merkityksellisyys |  |  |  |  |  |
| - 1. …reagoida myönteisesti toisten sote-ammattilaisten (samantasoisten tai sitä korkeampien) potilasturvallisuuteen liittyvien varoitusten tai huolenaiheiden ilmaisemiseen. | Oppiminen |  |  |  |  |  |
|  | Merkityksellisyys |  |  |  |  |  |
| - 1. … omaksua myönteisen asenteen, kun he varoittavat muita sote-ammattilaisia, jos nämä toiminnallaan jättävät huomiotta tärkeän potilasturvallisuusohjeen. | Oppiminen |  |  |  |  |  |
|  | Merkityksellisyys |  |  |  |  |  |
| - 1. …olla valmis jakamaan avoimesti ja suoraan erityisiä ehdotuksia potilasturvallisuuden parantamiseksi. | Oppiminen |  |  |  |  |  |
|  | Merkityksellisyys |  |  |  |  |  |
| - 1. …olla valmis oppimaan virheistä ja turvallisuuden vaaratapahtumista, joissa muut sote-ammattilaiset ovat olleet mukana, sen sijaan, että arvostelisi niitä. | Oppiminen |  |  |  |  |  |
|  | Merkityksellisyys |  |  |  |  |  |

**3. OSAAMINEN. Taidot**

Arvioi osaaminen (taidot) seuraavien kahden kriteerin mukaan:

*(1) Oppimisen aste* Missä määrin tulevat sote-ammattilaiset oppivat tällä hetkellä tämän osaamisen työharjoittelunsa kautta omassa organisaatiossasi?

1 Ei oppimista lainkaan – 5 Oppii täysin

*(2) Merkityksellisyyden aste.* Miten tärkeäksi arvioit sen, että psykologisen turvallisuuden ilmapiiriä voidaan edistää omassa organisaatiossasi, kun tulevat sote-ammattilaiset oppivat nämä taidot?

1 Ei lainkaan tärkeää – 5 Erittäin tärkeää

| **Taidot. Mielestäni harjoittelujaksot työpaikassani tarjoavat tulevalle sote-ammattilaiselle mahdollisuuden…** | **Asteikko** | **1** | **2** | **3** | **4** | **5** |
| --- | --- | --- | --- | --- | --- | --- |
| - 1. …kommunikoida avoimesti ja suoraan toiselle sote-ammattilaisille (samantasoisille tai sitä korkeammille) erityisiä potilasturvallisuuteen liittyviä huolenaiheita esittämällä tietoja, esittämällä kysymyksiä tai ilmaisemalla mielipiteitä. | Oppiminen |  |  |  |  |  |
|  | Merkityksellisyys |  |  |  |  |  |
| - 1. …pyytää vastuussa olevan sote-ammatillisen neuvoa havaitun ja tapahtuneen potilasturvallisuuden vaaratapahtuman raportoimiseksi potilasturvallisuuden vaaratapahtumajärjestelmään (tarvittaessa). | Oppiminen |  |  |  |  |  |
|  | Merkityksellisyys |  |  |  |  |  |
| - 1. …varoittaa vakuuttavasti toista sote-ammattilaista (samalla tasolla tai korkeammalla tasolla) siitä, että toiminnallaan he jättävät huomiotta tärkeän potilasturvallisuusohjeen. | Oppiminen |  |  |  |  |  |
|  | Merkityksellisyys |  |  |  |  |  |
| - 1. …vastata vakuuttavasti sen sote-ammattilaisen (samantasoisen tai korkeamman) negatiiviseen reaktioon, jota hän on varoittanut tärkeän potilasturvallisuusohjeen huomiotta jättämisestä. | Oppiminen |  |  |  |  |  |
|  | Merkityksellisyys |  |  |  |  |  |
| - 1. …sanallisesti tukea ja vahvistaa toisen sote-ammattilaisen (samantasoisten tai sitä korkeampien) esille tuomaa huolta potilasturvallisuudesta muun tiimin kanssa. | Oppiminen |  |  |  |  |  |
|  | Merkityksellisyys |  |  |  |  |  |
| - 1. … esittää konkreettisia ehdotuksia potilasturvallisuuden parantamiseksi omassa yksikössä tai omalla palvelualueella. | Oppiminen |  |  |  |  |  |
|  | Merkityksellisyys |  |  |  |  |  |
| - 1. …tarjota vertaistukea haittatapahtumaan osallisena olleelle kollegalle *second victim* -oireyhtymän vähentämiseksi (jolle on ominaista syyllisyyden, riittämättömyyden, ahdistuksen, häpeän, ylivireystila tai surun tunteet). | Oppiminen |  |  |  |  |  |
|  | Merkityksellisyys |  |  |  |  |  |

**4. TOIMENPITEET**

Arvioi kutakin toimenpidettä kahden kriteerin mukaisesti.:

*(1) Totetuttamisen aste*. Missä määrin tämä toimenpide on toteutettu omassa pääasiallisessa toimintaympäristössäsi?

1 Ei vielä toteutettu – 5 Täysin toteutettu

*(2) Merkityksellisyyden aste*. Miten tärkeäksi arvioit tämän toimenpiteen toteuttamisen psykologisen turvallisuuden ilmapiiriin edistämisen kannalta omassa organisaatiossasi?

1 Ei tärkeää lainkaan – 5 Erittäin tärkeää

| **Toimenpiteet. Oma organisaationi…** | **Asteikko** | **1** | **2** | **3** | **4** | **5** |
| --- | --- | --- | --- | --- | --- | --- |
| - 1. …toteuttaa koulutusohjelman uudelle henkilöstölle (erityisesti tuleville sote-ammattilaisille) edistääkseen positiivista potilasturvallisuuskulttuuria ja psykologisesti turvallista ilmapiiriä. | Toteuttaminen |  |  |  |  |  |
|  | Merkityksellisyys |  |  |  |  |  |
| - 1. …nimittää vaikutusvaltaisen ryhmän suunnittelemaan toimintasuunnitelman, jolla edistetään sote-ammattilaisten luottamusta herättävää ilmapiiriä potilasturvallisuuden varmistamiseksi. | Toteuttaminen |  |  |  |  |  |
|  | Merkityksellisyys |  |  |  |  |  |
| - 1. …järjestää säännöllisiä tilaisuuksia tulevien sote-ammattilaisten kanssa potilasturvallisuuteen liittyvien huolenaiheiden ja saatujen kokemusten jakamiseksi. Tämä toimenpide tarkoittaa yhteisiä tilaisuuksia, joissa vaihdetaan kokemuksia potilasturvallisuustapauksista, suunnitellaan suojauksia riskien minimoimiseksi ja tarjotaan emotionaalista ja käytännön tukea vertaisten keskuudessa. | Toteuttaminen |  |  |  |  |  |
|  | Merkityksellisyys |  |  |  |  |  |
| - 1. …lisää työyksikön sote-ammattilaisten tietoisuutta, yhteistyössä palvelupäälliköiden kanssa siitä, että tulevia sote-ammattilaisia ja työtovereita tulee kannustaa ilmaisemaan huolensa potilasturvallisuudesta avoimesti ja suoraan sekä varoittamaan muita sote-ammattilaisia riskeistä, joita he tunnistavat päivittäisessä työssään. | Toteuttaminen |  |  |  |  |  |
|  | Merkityksellisyys |  |  |  |  |  |
| - 1. …lisää työyksikön sote-ammattilaisten tietoisuutta, yhteistyössä palvelupäälliköiden kanssa siitä, että on tärkeää vastata myönteisesti muiden sote-ammattilaisten varoituksiin potilasturvallisuusohjeiden noudattamisesta ja vahvistaa tulevien sote-ammattilaisten mahdollisuuksia ilmaista avoimesti potilasturvallisuuteen liittyviä huoliaan. | Toteuttaminen |  |  |  |  |  |
|  | Merkityksellisyys |  |  |  |  |  |
| - 1. …tarjoaa tuleville sote-ammattilaisille mahdollisuuden osallistua tarkkailijoina suunniteltaessa haittatapahtumista käytäviä avoimia keskusteluja potilaan ja perheen kanssa. | Toteuttaminen |  |  |  |  |  |
|  | Merkityksellisyys |  |  |  |  |  |
| - 1. …antaa tuleville sote-ammattilaisille mahdollisuuden olla läsnä potilasturvallisuuden haittatapahtuman jälkeisessä keskustelussa ja analysoinnissa. | Toteuttaminen |  |  |  |  |  |
|  | Merkityksellisyys |  |  |  |  |  |
| - 1. …antaa tuleville sote-ammattilaisille erityistä koulutusta asianmukaisin keinoin potilasturvallisuuden vaaratapahtumista ilmoittamiseen. | Toteuttaminen |  |  |  |  |  |
|  | Merkityksellisyys |  |  |  |  |  |
| - 1. …tarjoaa organisaation tukea haittatapahtumaan osallisille sote-ammattilaisille parantaakseen työturvallisuutta. | Toteuttaminen |  |  |  |  |  |
|  | Merkityksellisyys |  |  |  |  |  |

**5. JOS HALUAT, voit tuoda esiin jotakin olennaista, mitä kyselystä puuttui ja mikä olisi tärkeää huomioida.**

|  |
| --- |

**6. LOPUKSI**

Maa: Kroatia / Viro / Suomi / Saksa / Israel / Liettua / Portugali / Slovakia / Espanja / Serbia / Muu

Ikä: _____________

Sukupuoli:  Mies  Nainen  Muu

Ammattiala:

Lääketiede

Hoitotyö

Farmasia

Synnytys ja naistentaudit

Fysioterapia

Psykologia

Sosiologia

Muu

Ohjauskokemuksesi (opiskelijat ja vasta valmistuneet) vuosina: _________

Niiden opiskelijoiden ja vastavalmistuneiden (tulevaisuuden sote-ammattilaisten) määrä, joita olet henkilökohtaisesti valvonut tai ohjannut/mentoroinut viimeisen kolmen vuoden aikana (2019-2021): _________

Onko yksikössäsi erityinen potilasturvallisuuden koulutusohjelma?  Kyllä  Ei

Kerro, missä työskentelet (kliininen työ ja ohjaaminen/mentorointi):

Perusterveydenhuolto

Erikoissairaanhoito (sairaala)

Sosiaalihuolto

**GERMAN VERSION**


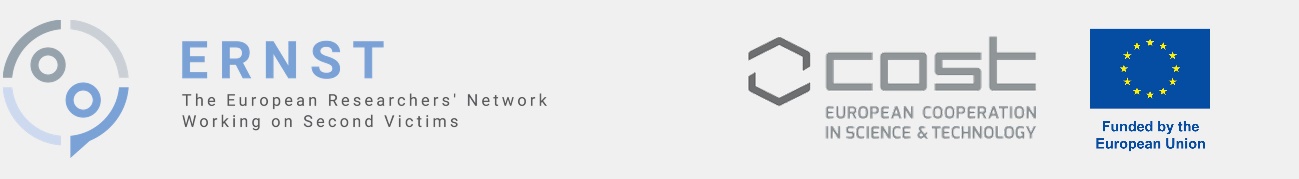


**Kompetenzen bei Ausbilder:innen im Gesundheitswesen zur Förderung psychologischer Sicherheitskultur im klinischen Setting**

Vielen Dank, dass Sie sich bereit erklärt haben, an dieser Studie teilzunehmen, die sich mit Kompetenzen im Bereich der psychologischen Sicherheitskultur beschäftigt, die Auszubildende im Gesundheitswesen im Verlauf ihrer Ausbildung erlernen.

Wir sind ein Forschungsteam aus dem akademischen und klinischen Bereich und Teil des europäischen ERNST Konsortiums, das sich auf Patientensicherheit und Second Victim Studien konzentriert. An dieser Studie sind Forscher:innen aus Kroatien, Estland, Finnland, Deutschland, Israel, Litauen, Portugal, Serbien, der Slowakei und Spanien beteiligt.

Psychologische Sicherheit bezieht sich auf die gemeinsame Überzeugung, dass ein Arbeitsteam gemeinsam komplexe Herausforderungen in einem Umfeld bewältigen kann, das von gegenseitigem Vertrauen und Respekt gekennzeichnet ist. So können Mitarbeitende im Gesundheitswesen eine sicherere Versorgung gewährleisten, wenn Ursachen und Möglichkeiten zur Vermeidung klinischer Fehler ohne Angst vor Konsequenzen oder Sanktionen diskutiert und analysiert werden können. Eine psychologische Sicherheitskultur kann ein entscheidender Faktor für die Patientensicherheit sein. Auf diese Weise wird eine sicherere Versorgung gewährleistet, sobald es möglich ist, Ursachen und Mechanismen zur Vermeidung klinischer Fehler zu diskutieren und analysieren ohne Angst vor Kritik oder Sanktionen.

Diese Delphi-Studie untersucht:

- Welche psychologischen Sicherheitskompetenzen (Wissen, Einstellungen und Fähigkeiten) künftige Angehörige der Gesundheitsberufe (Student:innen, Assistenzärzt:innen und Auszubildende, im Folgenden als “Trainees” bezeichnet) während ihrer klinischen Ausbildung erwerben
- Welche Maßnahmen in Ausbildungsstätten des Gesundheitswesens implementiert werden sollten, um den Erwerb dieser Kompetenzen zu fördern

Die Antworten auf den Fragebogen werden in pseudo-anonymisierter und aggregierter Form ausgewertet. Es gibt keine richtigen oder falschen Antworten auf die Fragen, der Zweck des Fragebogens ist es, Ihre Wahrnehmung als Ausbilder:in zu erfahren. Das Ausfüllen des Fragebogens wird etwa zehn Minuten Ihrer Zeit in Anspruch nehmen. Bitte vergessen Sie nicht, Ihre Antworten nach Abschluss des Fragebogens abzuschicken.

Sollten Sie Fragen oder Anmerkungen haben, schicken Sie bitte eine E-Mail an Ihren nationalen Koordinator (der Sie zur Teilnahme an der Studie eingeladen hat).

Vielen Dank im Voraus für Ihre Mitarbeit und Antworten.

**Informierte Einwilligung**

Die Teilnahme an dieser Studie ist freiwillig. Die gesammelten Daten werden pseudo-anonymisiert und in aggregierter Form analysiert. Die Plattform wird von einem sicheren Server in Spanien gehostet und von der Miguel Hernández Universität verwaltet, die Teil des öffentlichen spanischen Bildungssystems ist. Bitte bestätigen Sie, dass Sie auf freiwilliger Basis und in Kenntnis der Sachlage an dieser Studie teilnehmen. Sie können Ihre Zustimmung jederzeit widerrufen, indem Sie eine E-Mail an [___________](mailto:calite@umh.es) senden.

Ich erkläre mich bereit, an der Studie auf freiwilliger Basis und in Kenntnis der Sachlage teilzunehmen.

**1. KOMPETENZEN. Wissen**

Bitte bewerten Sie jede Kompetenz (Wissen) anhand zweier Kriterien:

*(1) Ausmaß des Erwerbs.* In welchem Ausmaß erwerben die Trainees diese Kompetenz derzeit im Rahmen ihrer klinischen Ausbildung an Ihrer Einrichtung?

1 Überhaupt kein Erwerb – 5 Vollständig erworben

*(2) Ausmaß der Bedeutung.* In welchem Ausmaß halten Sie diese Kompetenz für entscheidend als Beitrag zur psychologischen Sicherheitskultur in Ihrer Gesundheitseinrichtung?

1 Überhaupt nicht wichtig – 5 Sehr wichtig

| **Wissen. Meiner Meinung nach vermitteln Praktika in meiner Einrichtung Trainees die Kompetenzen, …** | **Ausmaß des/der** | **1** | **2** | **3** | **4** | **5** |
| --- | --- | --- | --- | --- | --- | --- |
| - 1. …zu verstehen, dass eine direkte und offene Kommunikation von Bedenken hinsichtlich der Patientensicherheit Ereignisse verhindern kann, die den Patient:innen schaden könnten. | Erwerb |  |  |  |  |  |
|  | Bedeutung |  |  |  |  |  |
| - 1. …zu wissen, wie sie selbstbewusst Bedenken hinsichtlich der Patientensicherheit gegenüber Kolleg:innen (auf gleichem Ausbildungsstand oder höher) kommunizieren können (Wortwahl, Tonfall, Gestik, etc.) | Erwerb |  |  |  |  |  |
|  | Bedeutung |  |  |  |  |  |
| - 1. … zu unterscheiden zwischen Situationen, die vermeidbaren Schaden am Patienten oder der Patientin anrichten könnten und denen, die kein hohes Risiko für die Patientensicherheit darstellen. | Erwerb |  |  |  |  |  |
|  | Bedeutung |  |  |  |  |  |
| - 1. …den besten Moment auszuwählen, um spezifische Bedenken hinsichtlich der Patientensicherheit mit eine:r Kolleg:in (auf gleichem Ausbildungsstand oder höher) zu besprechen. | Erwerb |  |  |  |  |  |
|  | Bedeutung |  |  |  |  |  |
| - 1. … Kolleg:innen (auf gleichem Ausbildungsstand oder höher) selbstbewusst vor dem Risiko, wichtige Regeln zur Patientensicherheit zu ignorieren, zu warnen (Wortwahl, Tonfall, Gestik, etc.). | Erwerb |  |  |  |  |  |
|  | Bedeutung |  |  |  |  |  |
| - 1. …zu wissen, wie man konstruktiv mit der möglichen negativen Reaktion von Kolleg:innen (auf gleichem Ausbildungsstand oder höher) umgeht, nachdem die Trainees das Übersehen einer wichtigen Regel der Patientensicherheit angemahnt haben. | Erwerb |  |  |  |  |  |
|  | Bedeutung |  |  |  |  |  |
| - 1. … zu wissen, wie spezifische Vorschläge gemacht werden können, um die Patientensicherheit im Bereich potenziell zu erhöhen. | Erwerb |  |  |  |  |  |
|  | Bedeutung |  |  |  |  |  |

**2. KOMPETENZEN. Verhalten**

Bitte bewerten Sie jede Kompetenz (Verhalten) anhand zweier Kriterien:

*(1) Ausmaß des Erwerbs.* In welchem Ausmaß erwerben die Trainees diese Kompetenz derzeit im Rahmen ihrer klinischen Ausbildung an Ihrer Einrichtung?

1 Überhaupt kein Erwerb – 5 Vollständig erworben

*(2) Ausmaß der Bedeutung.* In welchem Ausmaß halten Sie diese Kompetenz für entscheidend als Beitrag zur psychologischen Sicherheitskultur in Ihrer Gesundheitseinrichtung?

1 Überhaupt nicht wichtig – 5 Sehr wichtig

| **Verhalten. Meiner Meinung nach vermitteln Praktika in meiner Einrichtung Trainees die Kompetenzen, …** | **Ausmaß des/der** | **1** | **2** | **3** | **4** | **5** |
| --- | --- | --- | --- | --- | --- | --- |
| - 1. … sich der Identifikation und Verhinderung von Risiken bezüglich der Patientensicherheit zu widmen. | Erwerb |  |  |  |  |  |
|  | Bedeutung |  |  |  |  |  |
| - 1. … risikobehaftete Situationen im Arbeitsalltag als Chance wahrzunehmen, das Risiko zu betonen und passende Maßnahmen zu ergreifen, um Schäden zu verhindern. | Erwerb |  |  |  |  |  |
|  | Bedeutung |  |  |  |  |  |
| - 1. … positiv auf Warnungen oder Bedenken zu reagieren, die Kolleg:innen (auf gleichem Ausbildungsstand oder höher) in Bezug auf Patientensicherheit aussprechen. | Erwerb |  |  |  |  |  |
|  | Bedeutung |  |  |  |  |  |
| - 1. … es als positiv wahrzunehmen, andere Kolleg:innen zu warnen, wenn sie durch ihr Handeln eine wichtige Regel der Patientensicherheit ignorieren. | Erwerb |  |  |  |  |  |
|  | Bedeutung |  |  |  |  |  |
| - 1. … bereitwillig, offen und direkt spezifische Vorschläge anzubringen, um Patientensicherheit zu erhöhen. | Erwerb |  |  |  |  |  |
|  | Bedeutung |  |  |  |  |  |
| - 1. … bereit zu sein, von Fehlern und risikobehafteten Situationen zu lernen, in denen Kolleg:innen involviert waren und diese Kolleg:innen nicht zu verurteilen. | Erwerb |  |  |  |  |  |
|  | Bedeutung |  |  |  |  |  |

**3. KOMPETENZEN. Fähigkeiten**

Bitte bewerten Sie jede Kompetenz (Fähigkeiten) anhand zweier Kriterien:

*(1) Ausmaß des Erwerbs.* In welchem Ausmaß erwerben die Trainees diese Kompetenz derzeit im Rahmen ihrer klinischen Ausbildung an Ihrer Einrichtung?

1 Überhaupt kein Erwerb – 5 Vollständig erworben

*(2) Ausmaß der Bedeutung.* In welchem Ausmaß halten Sie diese Kompetenz für entscheidend als Beitrag zur psychologischen Sicherheitskultur in Ihrer Gesundheitseinrichtung?

1 Überhaupt nicht wichtig – 5 Sehr wichtig

| **Fähigkeiten. Meiner Meinung nach vermitteln Praktika in meiner Einrichtung Trainees die Kompetenzen, …** | **Ausmaß des/der** | **1** | **2** | **3** | **4** | **5** |
| --- | --- | --- | --- | --- | --- | --- |
| - 1. …offen und direkt mit Kolleg:innen (auf gleichem Ausbildungsstand oder höher) über spezifische Bedenken hinsichtlich der Patientensicherheit zu sprechen, indem sie Informationen vorlegen, Fragen stellen oder ihre Meinungen kundgeben. | Erwerb |  |  |  |  |  |
|  | Bedeutung |  |  |  |  |  |
| - 1. … die zuständigen Kolleg:innen um Hilfe zu bitten, ein beobachtetes Ereignis, das die Patientensicherheit betrifft, im passenden System einzutragen und (wenn nötig) es zu melden. | Erwerb |  |  |  |  |  |
|  | Bedeutung |  |  |  |  |  |
| - 1. … selbständig Kolleg:innen (auf gleichem Ausbildungsstand oder höher) zu warnen, wenn sie mit ihren Handlungen wichtige Regeln der Patientensicherheit ignorieren. | Erwerb |  |  |  |  |  |
|  | Bedeutung |  |  |  |  |  |
| - 1. … selbständig auf negative Reaktionen von Kolleg:innen (auf gleichem Ausbildungsstand oder höher), die die Trainees vor dem Ignorieren einer wichtigen Regel der Patientensicherheit gewarnt haben, zu reagieren. | Erwerb |  |  |  |  |  |
|  | Bedeutung |  |  |  |  |  |
| - 1. … verbal Initiativen von Kolleg:innen (auf gleichem Ausbildungsstand oder höher) zu unterstützen und bekräftigen, ihre spezifischen Bedenken hinsichtlich der Patientensicherheit mit dem Team zu teilen. | Erwerb |  |  |  |  |  |
|  | Bedeutung |  |  |  |  |  |
| - 1. … konkrete Vorschläge im eigenen Arbeitsbereich zu machen, um die Patientensicherheit zu erhöhen. | Erwerb |  |  |  |  |  |
|  | Bedeutung |  |  |  |  |  |
| - 1. … Kolleg:innen, die in einem unerwünschten Zwischenfall involviert waren, Unterstützung anzubieten, um das Second-Victim-Syndrom in seinen Auswirkungen zu reduzieren (charakterisiert durch Schuldgefühle, Nervosität, Schamgefühl, Trauer, Gefühl, Fehl am Platz zu sein, Hypervigilanz). | Erwerb |  |  |  |  |  |
|  | Bedeutung |  |  |  |  |  |

**4. INTERVENTIONEN**

Bitte bewerten Sie jede Intervention anhand zweier Kriterien:

*(1) Ausmaß der Implementierung.* In welchem Ausmaß erwerben die Trainees diese Kompetenz derzeit im Rahmen ihrer klinischen Ausbildung an Ihrer Einrichtung?

1 Überhaupt keine Implementierung – 5 Vollständig implementiert

*(2) Ausmaß der Bedeutung.* In welchem Ausmaß halten Sie diese Kompetenz für entscheidend als Beitrag zur psychologischen Sicherheitskultur in Ihrer Gesundheitseinrichtung?

1 Überhaupt nicht wichtig – 5 Sehr wichtig

| **INTERVENTIONEN. Meine Gesundheitseinrichtung…** | **Ausmaß der** | **1** | **2** | **3** | **4** | **5** |
| --- | --- | --- | --- | --- | --- | --- |
| - 1. … implementiert ein Trainingsprogramm für neue Angestellte (insbesondere Trainees), um eine positive Patientensicherheits- und psychologische Sicherheitskultur zu fördern. | Implementierung |  |  |  |  |  |
|  | Bedeutung |  |  |  |  |  |
| - 1. …weist eine einflussreiche Gruppe an Mitarbeiter:innen an, einen Interventionsplan zu entwerfen, um ein vertrauensvolles Klima untereinander zu fördern und Patientensicherheit zu gewährleisten. | Implementierung |  |  |  |  |  |
|  | Bedeutung |  |  |  |  |  |
| - 1. … hält regelmäßig klinische Sitzungen mit Trainees ab, um Bedenken und Erlerntes hinsichtlich der Patientensicherheit auszutauschen. Diese Maßnahme führt zu gemeinsamen Räumen, in denen Erfahrungen über Zwischenfälle im Bereich der Patientensicherheit ausgetauscht, Barrieren zur Risikominimierung entwickelt und emotionale und instrumentelle Unterstützung unter Kolleg:innen angeboten werden. | Implementierung |  |  |  |  |  |
|  | Bedeutung |  |  |  |  |  |
| - 1. … sensibilisiert die Fachkräfte der Einrichtung in Zusammenarbeit mit den Abteilungsleitungen für die Notwendigkeit, Trainees und Kolleg:innen zu ermutigen, ihre Bedenken hinsichtlich der Patientensicherheit offen und direkt zu äußern und andere Fachkräfte vor den Risiken zu warnen, die sie bei ihrer täglichen Arbeit erkennen. | Implementierung |  |  |  |  |  |
|  | Bedeutung |  |  |  |  |  |
| - 1. … sensibilisiert die Fachkräfte der Einrichtung in Zusammenarbeit mit den Abteilungsleitungen dafür, wie wichtig es ist, positiv auf Warnungen von Kolleg:innen hinsichtlich der Einhaltung der einschlägigen Vorschriften zur Patientensicherheit zu reagieren und die offene Kommunikation spezifischer Bedenken hinsichtlich der Patientensicherheit durch Trainees zu stärken. | Implementierung |  |  |  |  |  |
|  | Bedeutung |  |  |  |  |  |
| - 1. … bietet den Trainees die Möglichkeit, an der Planung von Gesprächen über unerwünschte Zwischenfälle mit den betroffenen Patient:innen und deren Angehörigen teilzunehmen. | Implementierung |  |  |  |  |  |
|  | Bedeutung |  |  |  |  |  |
| - 1. … bietet Trainees die Möglichkeit, bei Diskussionen und Analysen von Zwischenfällen im Bereich der Patientensicherheit anwesend zu sein. | Implementierung |  |  |  |  |  |
|  | Bedeutung |  |  |  |  |  |
| - 1. … bietet Trainees spezielle Schulungen zur Meldung von Zwischenfällen im Bereich der Patientensicherheit mit geeigneten Mitteln an. | Implementierung |  |  |  |  |  |
|  | Bedeutung |  |  |  |  |  |
| - 1. … bietet Mitarbeiter:innen institutionelle Unterstützung an, wenn sie in einem unerwünschten Zwischenfall involviert waren, um zu einer besseren Arbeitssicherheit beizutragen. | Implementierung |  |  |  |  |  |
|  | Bedeutung |  |  |  |  |  |

**5. WENN SIE MÖCHTEN, können Sie hier Ergänzungen machen, die im Fragebogen fehlen oder was Sie für wichtig halten, hier extra aufzuführen oder zu vertiefen.**

|  |
| --- |

**6. ABSCHLUSS, bitte geben Sie an.**

Land: Kroatien / Estland / Finnland / Deutschland / Israel / Litauen / Portugal / Slowakei / Spanien / Serbien / Sonstiges

Alter: _____________

Geschlecht:  Mann  Frau  Divers

Arbeitsfeld:

Medizin

Pflege

Pharmazie

Geburtshilfe

Physiotherapie

Psychologie

Soziologie

Sonstiges

Jahre, die Sie bereits Ausbilder:in, Mentor:in, etc. sind: _________

Anzahl der Trainees, die Sie persönliche beaufsichtigt oder betreut haben innerhalb der letzten drei Jahre (2019-2021):_________

Gibt es ein spezielles Ausbildungsprogramm zur Patientensicherheit in Ihrer Einrichtung?  Ja  Nein

Umfeld, in dem Sie klinisch und als Mentor:in tätig sind:

Grundversorgung

Spezielle Versorgung (Krankenhaus)

Sozialarbeit

**HEBREW VERSION**


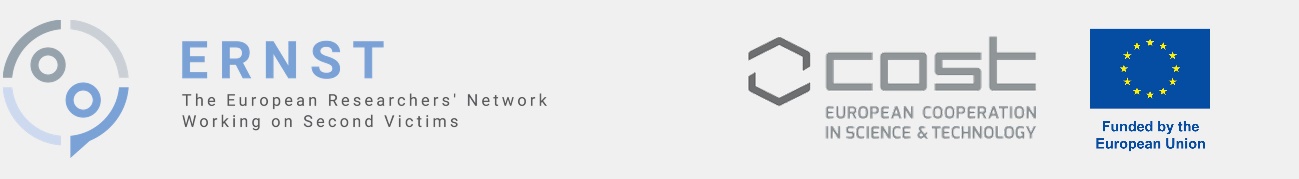
 מיומנויות בקרב מתמחים וסטודנטים בתחום הבריאות לקידום אקלים של בטיחות פסיכולוגית במסגרות קליניות

תודה על הסכמתך להשתתף במחקר זה על כישורי בטיחות פסיכולוגית שנרכשו על ידי סטאז'רים וסטודנטים של דיסציפלינות בריאות במהלך תקופת ההכשרה שלהם.

אנחנו צוות של חוקרים מהתחומים האקדמיים והקליניים, חלק מהקונסורציום האירופי ERNST המתמקד בבטיחות המטופלים ובמחקרים של קורבנות משניים. במחקר זה מעורבים חוקרים מקרואטיה, אסטוניה, פינלנד, גרמניה, ישראל, ליטא, פורטוגל, סלובקיה וספרד.

בטיחות פסיכולוגית מתייחסת לאמונה המשותפת שצוות עבודה יכול להתמודד עם אתגרים מורכבים כאשר יש סביבה של כבוד הדדי ואמון. לפיכך, אנשי מקצוע בתחום הבריאות מספקים טיפול בטוח יותר כאשר ניתן לדון ולנתח סיבות ודרכים להימנעות מטעויות קליניות ללא חשש מביקורת או סנקציות. אקלים בטיחותי פסיכולוגי יכול להיות גורם מכריע בבטיחות המטופל. בדרך זו, זה מספק טיפול בטוח יותר כאשר ניתן לדון ולנתח את הסיבות וכיצד להימנע מטעויות קליניות ללא חשש מביקורת או סנקציות.

מחקר דלפי זה בוחן:

- אילו יכולות בטיחות פסיכולוגיות (ידע, עמדות ומיומנויות) נרכשות על ידי הדורות הבאים של אנשי מקצוע בתחום הבריאות (סטודנטים וסטאז'רים יכונו משלב זה ואילך "מתלמדים") באמצעות ההתמחות הקלינית שלהם.
- אילו פעולות על מוסדות הבריאות שבהם מתלמדים עושים את ההתמחות הקלינית שלהם לבצע כדי לקדם את רכישת מיומנויות אלו.

התשובות לשאלון ינותחו בצורה פסאודו אנונימית ומצטברת. אין תשובות נכונות או לא נכונות לשאלות, אבל המטרה היא לדעת את התפיסה שלך כמדריך עבור מתלמד. מילוי השאלון ייקח כ-10 דקות. נא לא לשכוח לשלוח את תשובותיכם בסוף השאלון.

לכל שאלה או הערה, אנא שלח דואר אלקטרוני לרכז הלאומי שלך (שהזמין אותך להשתתף במחקר) או לכתובת __________

אנו מודים לך מראש על שיתוף הפעולה ותגובותיך.

**הסכמה מדעת**

ההשתתפות במחקר זה הינה בהתנדבות. הנתונים שיאספו יהיו פסאודו אנונימיים וינותחו בצורה מצטברת. הפלטפורמה ממוקמת בשרת מאובטח שנמצא בספרד ומנוהל על ידי אוניברסיטת Miguel Hernández, חלק ממערכת החינוך הציבורית הספרדית. אנא אשר כי אתה משתתף במחקר זה על בסיס התנדבותי ומושכל. אתה יכול לבטל את הסכמתך להשתתף במחקר בכל עת על ידי שליחת דוא"ל לכתובת [_________](file:///C:\Users\micohen\AppData\Local\Microsoft\Windows\INetCache\Content.Outlook\OHU30GMU\calite@umh.es)

אני מסכים להשתתף במחקר על בסיס וולונטרי ומושכל.

1. יכולות, ידע

נא להעריך כל יכולת (ידע) לפי שני קריטריונים:

(1) מידת הרכישה. באיזו מידה מתלמדים רוכשים כיום יכולת זו באמצעות ההתמחות הקלינית שלהם במוסד שלך?

1 אין רכישה כלל - 5 נרכש במלואו

(2) מידת החשיבות. באיזו מידה לדעתך יכולת זו חשובה לאקלים הבטיחות הפסיכולוגית במוסד הבריאות?

1 לא חשוב בכלל - 5 חשוב מאוד

| ידע. לדעתי, התמחות בסביבת העבודה שלי מקנה למתלמדים את היכולת... | מידת ה- | 1 | 2 | 3 | 4 | 5 |
| --- | --- | --- | --- | --- | --- | --- |
| 1.1 להבין שביטוי פתוח וישיר של דאגות לגבי בטיחות המטופל יכול למנוע התרחשות של אירועים שעלולים לגרום נזק למטופל. | רכישה |  |  |  |  |  |
|  | חשיבות |  |  |  |  |  |
| 1.2 לדעת איך לתקשר באסרטיביות דאגה לגבי בטיחות המטופל לגורם רפואי אחר (באותה רמה ומעלה) (אילו מילים לבחור, איך להתחיל ולסיים את השיחה, באיזה טון דיבור או מחוות להשתמש וכו'). | רכישה |  |  |  |  |  |
|  | חשיבות |  |  |  |  |  |
| 1.3 להבחין בין מצבים שעלולים לגרום לנזק בלתי נמנע למטופל מאלה שאינם מהווים סיכון גבוה לבטיחות המטופל | רכישה |  |  |  |  |  |
|  | חשיבות |  |  |  |  |  |
| 1.4 לבחור את הרגע הטוב ביותר על מנת לתקשר דאגות ספציפיות לגבי בטיחות המטופלים לגורם רפואי אחר (באותה רמה ומעלה). | רכישה |  |  |  |  |  |
|  | חשיבות |  |  |  |  |  |
| 1.5 לדעת איך להזהיר באסרטיביות איש מקצוע רפואי אחר (באותה רמה ומעלה) לגבי הסיכון מהתעלמות מכלל חשוב לבטיחות המטופל (מילים לבחירה, איך להתחיל ולסיים את השיחה, באיזה טון דיבור או מחוות להשתמש וכו.). | רכישה |  |  |  |  |  |
|  | חשיבות |  |  |  |  |  |
| 1.6 לדעת איך להתמודד בצורה בונה עם התגבוה השלילית האפשרית של איש מקצוע בתחום הבריאות (באותה רמה ומעלה) לאחר שהזהיר אותם שהם מתעלמים מכלל ציות חשוב לבטיחות המטופל. | רכישה |  |  |  |  |  |
|  | חשיבות |  |  |  |  |  |
| 1.7 לדעת להביע הצעות ספציפיות שיכולות לשפר את בטיחות המטופל ביחידה. | רכישה |  |  |  |  |  |
|  | חשיבות |  |  |  |  |  |

2. יכולות, עמדות

נא להעריך כל יכולת (עמדות) לפי שני קריטריונים:

(1) מידת הרכישה. באיזו מידה מתלמדים רוכשים כיום יכולת זו באמצעות ההתמחות הקלינית שלהם במוסד שלך?

1 אין רכישה כלל - 5 נרכש במלואו

(2) מידת החשיבות. באיזו מידה לדעתך יכולת זו חשובה לאקלים הבטיחות הפסיכולוגית במוסד הבריאות?

1 לא חשוב בכלל - 5 חשוב מאוד

| עמדה. לדעתי, התמחות בסביבת העבודה שלי מקנה למתלמדים את היכולת... | מידת ה- | 1 | 2 | 3 | 4 | 5 |
| --- | --- | --- | --- | --- | --- | --- |
| 2.1 להתחייב לזיהוי ולמניעת סיכונים לבטיחות החולה. | רכישה |  |  |  |  |  |
|  | חשיבות |  |  |  |  |  |
| 2.2 לתפוס מצבי סיכון בעבודה יומיומית כהזדמנות להדגש את הסיכון ולנקוט באמצעים מתאימים על מנת למנוע נזק מהמטופלים. | רכישה |  |  |  |  |  |
|  | חשיבות |  |  |  |  |  |
| 2.3 להגיב בחיוב להבעת אזהרות או דאגות שאנשי מקצוע אחרים בתחום הבריאות (באותה רמה ומעלה) מעלים ביחס לבטיחות המטופל. | רכישה |  |  |  |  |  |
|  | חשיבות |  |  |  |  |  |
| 2.4 לשמור על גישה חיובית להזהיר אנשי מקצוע אחרים בתחום הבריאות אם במעשיהם הם מתעלמים מכללי חשובים לבטיחות החולים. | רכישה |  |  |  |  |  |
|  | חשיבות |  |  |  |  |  |
| 2.5 להיות מוכן לשתף באופן גלוי וישיר הצעות ספציפיות לשיפור בטיחות המטופל. | רכישה |  |  |  |  |  |
|  | חשיבות |  |  |  |  |  |
| 2.6 להיות מוכן ללמוד מטעויות ומאירועי בטיחות שבהם היו מעורבים אנשי מקצוע אחרים, במקום לשפוט אותם. | רכישה |  |  |  |  |  |
|  | חשיבות |  |  |  |  |  |

**3. יכולות, כישורים**

נא להעריך כל יכולת (כישור) לפי שני קריטריונים:

(1) מידת הרכישה. באיזו מידה מתלמדים רוכשים כיום יכולת זו באמצעות ההתמחות הקלינית שלהם במוסד שלך?

1 אין רכישה כלל - 5 נרכש במלואו

(2) מידת החשיבות. באיזו מידה לדעתך יכולת זו חשובה לאקלים הבטיחות הפסיכולוגית במוסד הבריאות?

1 לא חשוב בכלל - 5 חשוב מאוד

| כישורים. לדעתי, התמחות בסביבת העבודה שלי מקנה למתלמדים את היכולת... | מידת ה- | 1 | 2 | 3 | 4 | 5 |
| --- | --- | --- | --- | --- | --- | --- |
| 3.1 לתקשר באופן גלוי וישיר עם אנשי מקצוע אחרים (באותה רמה ומעלה) דאגות ספציפיות לגבי בטיחות המטופל על ידי הצגת מידע, שאילת שאלות או הבעת דעות. | רכישה |  |  |  |  |  |
|  | חשיבות |  |  |  |  |  |
| 3.2 לבקש מהיועץ המקצועי האחראי לדווח, במערכת המתאימה, על התרחשות אירוע בטיחות החולה שהיה עדים לו ולבצע את הדיווח (במידת הצורך). | רכישה |  |  |  |  |  |
|  | חשיבות |  |  |  |  |  |
| 2.3 להגיב בחיוב להבעת אזהרות או דאגות שאנשי מקצוע אחרים בתחום הבריאות (באותה רמה ומעלה) מעלים ביחס לבטיחות המטופל. | רכישה |  |  |  |  |  |
|  | חשיבות |  |  |  |  |  |
| 3.4 להגיב באסרטיביות לתגובה שלילית של איש מקצוע בתחום הבריאות (באותה רמה ומעלה) שהזהרת מפני התעלמות מכללי בטיחות מטופלים חשובים. | רכישה |  |  |  |  |  |
|  | חשיבות |  |  |  |  |  |
| 3.5 לתמוך מילולית ולחזק את היוזמה של אנשי מקצוע אחרים בתחום הבריאות (באותה רמה ומעלה) לחלוק את דאגותיהם הספציפיות לגבי בטיחות המטופל עם שאר הצוות. | רכישה |  |  |  |  |  |
|  | חשיבות |  |  |  |  |  |
| 3.6 להגדיר ולהעביר הצעות קונקרטיות לשיפור בטיחות המטופלים ביחידה או במוסד. | רכישה |  |  |  |  |  |
|  | חשיבות |  |  |  |  |  |
| 3.7 להציע תמיכת עמיתים לעמית המעורב באירוע שלילי כדי להפחית את תסמונת הקורבן השני (המאופיינת ברגשות אשמה, חוסר התאמה, חרדה, בושה, ערנות יתר או אבל). | רכישה |  |  |  |  |  |
|  | חשיבות |  |  |  |  |  |

4. התערבויות

נא להעריך כל התערבות לפי שני קריטריונים:

(1) מידת היישום. באיזו מידה מתלמדים רוכשים כיום יכולת זו באמצעות ההתמחות הקלינית שלהם במוסד שלך?

1 עדיין לא מיושם - 5 יושם במלואו

(2) מידת החשיבות. באיזו מידה לדעתך יכולת זו חשובה לאקלים הבטיחות הפסיכולוגית במוסד הבריאות?

1 לא חשוב בכלל - 5 חשוב מאוד

| התערבויות. המוסד שלי: | מידת ה- | 1 | 2 | 3 | 4 | 5 |
| --- | --- | --- | --- | --- | --- | --- |
| 4.1 ליישם תוכנית הכשרה לצוות חדש (במיוחד למתלמדים) כדי לטפח תרבות בטיחות חיובית למטופל ואקלים בטיחותי פסיכולוגי. | יישום |  |  |  |  |  |
|  | חשיבות |  |  |  |  |  |
| 4.2 למנות קבוצה משפיעה של אנשים לתכנן תוכנית התערבות כדי לטפח אקלים בוטח בקרב אנשי מקצוע בתחום הבריאות כדי להבטיח את בטיחות המטופל. | יישום |  |  |  |  |  |
|  | חשיבות |  |  |  |  |  |
| 4.3 לקיים מפגשים קליניים קבועים עם מתלמדים כדי לשתף בדאגות בטיחות המטופלים ובלקחים שנלמדו. אמצעי זה מתורגם למערך של מרחבים משותפים להחלפת חוויות על אירועי בטיחות מטופלים, להמציא מחסומים כדי למזער סיכונים ולספק תמיכה רגשית ואינסטרומנטלית בקרב עמיתים. | יישום |  |  |  |  |  |
|  | חשיבות |  |  |  |  |  |
| 4.4 להעלות את המודעות בקרב אנשי המקצוע של המוסד, בשיתוף המנהלים בו, לצורך עידוד מתלמדים ועמיתים להביע את חששותיהם בנוגע לבטיחות החולה באופן גלוי וישיר ולהזהיר אנשי מקצוע אחרים מפני הסיכונים שהם מזהים בעבודתם היומיומית. | יישום |  |  |  |  |  |
|  | חשיבות |  |  |  |  |  |
| 4.5 להעלות את המודעות בקרב אנשי המקצוע של המוסד, בשיתוף המנהלים בו, לחשיבות היענות חיובית לאזהרות של אנשי מקצוע אחרים בנוגע לעמידה בכללי בטיחות החולה הרלוונטיים ולחזק את הביטוי הגלוי לחששות ספציפיים לבטיחות החולה מצד המתלמדים. | יישום |  |  |  |  |  |
|  | חשיבות |  |  |  |  |  |
| 4.6 לספק למתלמדים את ההזדמנות להשתתף כמשקיפים במהלך תכנון שיחות גילוי אירועים חריגים עם החולה המושפע ומשפחתו. | יישום |  |  |  |  |  |
|  | חשיבות |  |  |  |  |  |
| 4.7 לאפשר למתלמדים הזדמנות להיות נוכחים במהלך הדיון והניתוח בעקבות אירוע בטיחות למטופל. | יישום |  |  |  |  |  |
|  | חשיבות |  |  |  |  |  |
| 4.8 לספק למתלמדים הכשרה ספציפית בנושא דיווח על אירועי בטיחות מטופלים באמצעים מתאימים | יישום |  |  |  |  |  |
|  | חשיבות |  |  |  |  |  |
| 4.9 להציע תמיכה מוסדית לאנשי מקצוע בתחום הבריאות המעורבים באירוע חריג כדי לתרום לבטיחות טובה יותר במקום העבודה. | יישום |  |  |  |  |  |
|  | חשיבות |  |  |  |  |  |

5. אם תרצו, השתמשו במקום הזה כדי להוסיף משהו שפספסתם בשאלון או כל דבר שנראה לכם לנכון לציין לגבי הנושא הנדון.

|  |
| --- |

6. לסיום, נא לציין

גיל: _____________

מין:  גבר  אישה  אחר

מקצוע:

רפואה

סיעוד

רוקחות

מיילדות

פיזיותרפיה

פסיכולוגיה

סוציולוגיה

אחר

שנות הדרכה: _________

כמות מתלמדים שליווית או הדרכת באופן אישי בשלוש השנים האחרונות (2019-2021): _________

האם יש במוסד שלך תוכנית הכשרה ספציפית בבטיחות המטופלים?  כן  לא

הגדרת המקום שבו אתה מבצע את עבודתך הקלינית וההדרכה:

טיפול ראשוני

טיפול מיוחד (בית חולים)

טיפול סוציאלי

**PORTUGUESE VERSION**


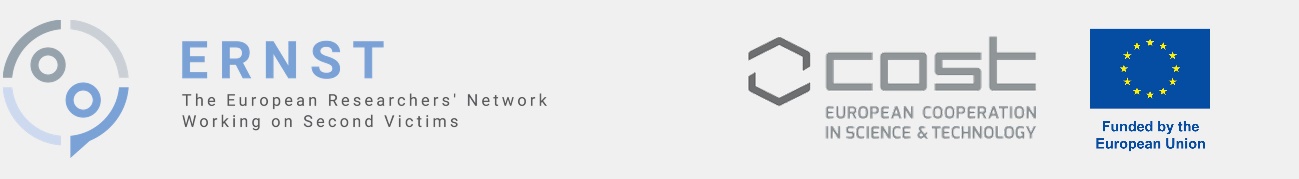


**Competências presentes em estagiários que promovem um clima de segurança psicológica em contexto clínico**

Agradecemos por participar no presente estudo centrado em competências no âmbito da segurança psicológica (“psychological safety”), adquiridas por estagiários na área da saúde durante os seus períodos de educação clínica/estágios clínicos.

Somos uma equipa de investigadores académicos e clínicos, parte da Rede Europeia ERNST (*The European Researchers’ Network Working on Second Victims*) focada em estudar aspetos relacionados com segurança do doente e o fenómeno da segunda vítima. Este estudo envolve investigadores de diferentes países: Croácia, Estónia, Finlândia, Alemanha, Israel, Lituânia, Portugal, Eslováquia, Espanha e Sérvia.

Segurança psicológica refere-se a um princípio partilhado de que o trabalho em equipa envolve um ambiente de respeito mútuo e de confiança. Deste modo, os profissionais de saúde disponibilizam cuidados mais seguros quando as causas dos incidentes e formas de os prevenir podem ser discutidas e analisadas sem estar associado o medo da crítica e de sanções disciplinares.

O estudo de Delphi explora:

- que competências no âmbito da segurança psicológica (conhecimento, atitudes e capacidades) estão a ser adquiridas pelas futuras gerações de profissionais de saúde (estudantes, internos, estagiários, neste estudo definidos como “estagiários”) através da sua educação clínica/estágios;
- que ações as instituições de saúde, em que os estagiários fazem os seus estágios clínicos, devem implementar para promover a aquisição destas competências;

As respostas a este questionário serão analisadas, pseudo-anonimizadas e agregadas. Neste questionário, não existem respostas certas ou erradas. O objetivo é compreender a sua perceção enquanto orientador de estágio. O questionário demora aproximadamente 10 minutos a completar. Por favor, não se esqueça de finalizar o questionário clicando no final do questionário.

Para qualquer questão ou comentário, por favor envie um email para o seu coordenador nacional (que o convidou a participar neste estudo.

Agradecemos desde já a sua cooperação e disponibilidade para integrar este estudo.

**Consentimiento informado**

A participação neste estudo é voluntária. Os dados recolhidos irão ser anonimizados (através da utilização de pseudónimos) e analisados de forma agregada. A plataforma de recolha de dados está sediada num servidor seguro, localizado em Espanha e esta é gerenciada pela Universidade Miguel Hernández, parte do sistema educacional público espanhol. Por favor, confirme que está a participar no estudo de forma voluntária e informada. Poderá revogar o seu consentimento para participar neste estudo em qualquer momento, enviando um email para __________

Eu aceito participar no estudo de forma voluntária e informada.

**1. COMPETÊNCIAS. Conhecimento**

Por favor, avalie cada competência (conhecimento) de acordo com dois critérios:

*(1) Grau de aquisição*. A que nível os estagiários adquirem essa competência através dos estágios clínicos na sua instituição?

1- Sem aquisição - 5 totalmente adquirido

*(2) Grau de significância*. A que nível considera que essa competência contribui decisivamente para um clima de segurança psicológica na sua instituição de saúde?

1 Não é importante- 5 Muito importante

| **Conhecimento. Na minha opinião, os estágios no meu contexto institucional permitem aos estagiários ter a competência de ...** | **Nível de** | **1** | **2** | **3** | **4** | **5** |
| --- | --- | --- | --- | --- | --- | --- |
| - 1. …compreenderem que uma expressão de preocupação, de forma aberta e direta, relacionada com aspetos de segurança do doente pode prevenir a ocorrência de incidentes que possam causar danos ao doente. | Aquisição |  |  |  |  |  |
|  | Significância |  |  |  |  |  |
| - 1. … saberem como comunicar assertivamente uma preocupação acerca da segurança do doente a outro profissional de saúde (do mesmo nível profissional ou superior) (que palavras escolher, como começar e terminar uma conversa, que tom de voz utilizar e gestos utilizar, etc). | Aquisição |  |  |  |  |  |
|  | Significância |  |  |  |  |  |
| - 1. … distinguirem entre situações que podem causar dano evitável ao doente daquelas que não representam um risco elevado para a segurança do doente. | Aquisição |  |  |  |  |  |
|  | Significância |  |  |  |  |  |
| - 1. … escolherem o melhor momento para comunicarem preocupações específicas acerca da segurança do doente a outro profissional de saúde ( do mesmo nível profissional ou superior). | Aquisição |  |  |  |  |  |
|  | Significância |  |  |  |  |  |
| - 1. … saberem como avisar assertivamente outro profissional de saúde (do mesmo nível profissional ou superior) acerca do risco de ignorar uma orientação/principio importante da segurança do doente (escolher as palavras que utilizar, como começar e terminar uma conversa, que tom de voz utilizar e gestos utilizar, etc). | Aquisição |  |  |  |  |  |
|  | Significância |  |  |  |  |  |
| - 1. … saberem como lidar de forma construtiva com uma possível reação negativa por parte de um profissional de saúde (do mesmo nível profissional ou superior) depois deste ter sido avisado que houve uma negligência importante de uma questão relacionada com a segurança do doente. | Aquisição |  |  |  |  |  |
|  | Significância |  |  |  |  |  |
| - 1. … saberem como fazer propostas especificas que melhorem a segurança do doente da unidade de saúde. | Aquisição |  |  |  |  |  |
|  | Significância |  |  |  |  |  |

**2. COMPETÊNCIAS. Atitudes**

Por favor, avalie cada competência (atitude) de acordo com dois critérios:

*(1) Grau de aquisição*. A que nível os estagiários adquirem essa competência através dos estágios clínicos na sua instituição?

1- Sem aquisição - 5 totalmente adquirido

*(2) Grau de significância*. A que nível considera que essa competência contribui decisivamente para um clima de segurança psicológica na sua instituição de saúde?

1 Não é importante- 5 Muito importante

| **Atitude. Na minha opinião, os estágios no meu contexto institucional permitem aos estagiários...** | **Nível de** | **1** | **2** | **3** | **4** | **5** |
| --- | --- | --- | --- | --- | --- | --- |
| - 1. … comprometerem-se com a identificação e prevenção de riscos para a segurança do doente. | Aquisição |  |  |  |  |  |
|  | Significância |  |  |  |  |  |
| - 1. … percecionarem situações de risco no trabalho diário como uma oportunidade para realçar o risco existente e tomar medidas apropriadas para prevenir futuros danos aos doentes. | Aquisição |  |  |  |  |  |
|  | Significância |  |  |  |  |  |
| - 1. … responderem de forma positiva a preocupações ou chamadas de atenção por parte de outros profissionais de saúde (do mesmo nível ou de nível superior) relacionadas com aspetos de segurança do doente. | Aquisição |  |  |  |  |  |
|  | Significância |  |  |  |  |  |
| - 1. … manterem uma atitude positiva alertando outros profissionais de saúde se estes ignoram aspetos importantes de segurança do doente. | Aquisição |  |  |  |  |  |
|  | Significância |  |  |  |  |  |
| - 1. ...estarem dispostos a partilhar, de forma aberta e direta, propostas específicas para melhorar a segurança do doente. | Aquisição |  |  |  |  |  |
|  | Significância |  |  |  |  |  |
| - 1. … estarem dispostos a aprender a partir dos erros e de incidentes que comprometeram a segurança dos doentes em que outros profissionais estiveram envolvidos, em vez de julgá-los. | Aquisição |  |  |  |  |  |
|  | Significância |  |  |  |  |  |

**3. COMPETÊNCIAS. Capacidades**

Por favor, avalie cada competência (capacidade) de acordo com dois critérios:

*(1) Grau de aquisição.* A que nível os estagiários adquirem essa competência através dos estágios clínicos na sua instituição?

1- Sem aquisição - 5 totalmente adquirido

*(2) Grau de significância*. A que nível considera que essa competência contribui decisivamente para um clima de segurança psicológica na sua instituição de saúde?

1 Não é importante- 5 Muito importante

| **Capacidades. Na minha opinião, os estágios no meu contexto institucional permitem aos estagiários...** | **Nível de** | **1** | **2** | **3** | **4** | **5** |
| --- | --- | --- | --- | --- | --- | --- |
| - 1. …comunicarem de forma aberta e direta com outros profissionais (do mesmo nível profissional ou superior) preocupações específicas relacionadas com a segurança do doente, utilizando diversas abordagens, tais como apresentação de informação, fazerem questões ou expressarem opiniões. | Aquisição |  |  |  |  |  |
|  | Significância |  |  |  |  |  |
| - 1. … pedirem a profissionais qualificados apoio nos registos, num sistema apropriado, dum incidente de segurança do doente que foi testemunhado e fazerem a notificação (se necessário) | Aquisição |  |  |  |  |  |
|  | Significância |  |  |  |  |  |
| - 1. … alertarem assertivamente outro profissional de saúde (do mesmo nível profissional ou superior) que através das suas ações está a ignorar aspetos importantes relacionados com a segurança do doente. | Aquisição |  |  |  |  |  |
|  | Significância |  |  |  |  |  |
| - 1. … responderem de forma assertiva a reações negativas por parte de um profissional de saúde (do mesmo nível profissional ou superior) após ser alertado por ter ignorado aspetos importantes relacionados com segurança do doente. | Aquisição |  |  |  |  |  |
|  | Significância |  |  |  |  |  |
| - 1. … manifestarem apoio e reforçarem iniciativas de partilha de preocupações específicas relacionadas com a segurança do doente por parte de outros profissionais de saúde (do mesmo nível profissional ou superior). | Aquisição |  |  |  |  |  |
|  | Significância |  |  |  |  |  |
| - 1. …definirem e comunicarem propostas concretas para melhoria da segurança do doente na sua unidade de saúde ou serviço. | Aquisição |  |  |  |  |  |
|  | Significância |  |  |  |  |  |
| - 1. … darem apoio a um colega envolvido num evento adverso de forma a reduzir as consequências sentidas pelas segundas vítimas, ou seja, profissionais de saúde que manifestam sentimentos como culpa, insuficiência, ansiedade, vergonha, hipervigilância ou tristeza. | Aquisição |  |  |  |  |  |
|  | Significância |  |  |  |  |  |

**4. INTERVENÇÕES**

Por favor, avalie cada intervenção de acordo com dois critérios:

*(1) Grau de implementação*. A que nível esta intervenção foi implementada no seu contexto clínico?

1 Não foi ainda implementado – 5 Totalmente implementado

*(2) Grau de significância*. Em que nível considera que esta intervenção contribui decisivamente para um clima de segurança psicológica na sua instituição de saúde?

1 Nada importante – 5 Muito importante

| **Intervenções. A minha instituição de saúde ….** | **Nível de** | **1** | **2** | **3** | **4** | **5** |
| --- | --- | --- | --- | --- | --- | --- |
| - 1. …implementa um programa de formação para novos profissionais (especialmente estagiários) para promover uma cultura de segurança do doente e um clima de segurança psicológica. | Implementação |  |  |  |  |  |
|  | Significância |  |  |  |  |  |
| - 1. … nomeia um grupo de pessoas para elaborar/desenhar um plano de intervenção de modo a promover um clima de confiança entre os profissionais de saúde e garantir a segurança do doente. | Implementação |  |  |  |  |  |
|  | Significância |  |  |  |  |  |
| - 1. … realiza sessões clínicas regulares com estagiários para partilhar as preocupações relacionadas com a segurança do doente e lições aprendidas. Essa medida traduz-se num conjunto de espaços compartilhados para troca de experiências sobre incidentes de segurança do doente, construção de barreiras para minimizar riscos e dar apoio emocional e instrumental entre pares. | Implementação |  |  |  |  |  |
|  | Significância |  |  |  |  |  |
| - 1. … sensibiliza os profissionais da instituição, com a colaboração dos chefes de serviço, para a necessidade de encorajar os estagiários e colegas a expressarem as suas preocupações relacionadas com a segurança do doente, de forma aberta e direta, bem como alertarem os outros profissionais acerca dos riscos que são identificados durante a prática clínica diária. | Implementação |  |  |  |  |  |
|  | Significância |  |  |  |  |  |
| - 1. … sensibiliza os profissionais da instituição, com a colaboração dos chefes de serviço, para a importância de responderem positivamente às afirmações/avisos por parte de outros profissionais de saúde, relacionados com cumprimento de aspetos/orientações no âmbito da segurança do doente e reforça a expressão aberta de preocupações específicas da área da segurança do doente por parte dos estagiários | Implementação |  |  |  |  |  |
|  | Significância |  |  |  |  |  |
| - 1. … oferece aos estagiários a oportunidade de participarem como observadores durante o planeamento de conversas “disclosure”, ou seja, conversas que falam de forma aberta e direta acerca do incidente que sucedeu e como este afetou o doente e a sua família. | Implementação |  |  |  |  |  |
|  | Significância |  |  |  |  |  |
| - 1. …permite que os estagiários estejam presentes durante a discussão e análise de incidentes que comprometem a segurança dos doentes. | Implementação |  |  |  |  |  |
|  | Significância |  |  |  |  |  |
| - 1. … disponibiliza para estagiários um treino específico, através de meios adequados, centrado na notificação de incidentes que comprometem a segurança do doente | Implementação |  |  |  |  |  |
|  | Significância |  |  |  |  |  |
| - 1. … oferece apoio institucional aos profissionais de saúde envolvidos em eventos adversos de modo a contribuir para uma melhor segurança no trabalho e para doente . | Implementação |  |  |  |  |  |
|  | Significância |  |  |  |  |  |

1. **CASO CONSIDERE NECESSÁRIO, utilize este espaço para adicionar algo que considera que está em falta neste questionário ou algo que considera apropriado referir relativamente ao tópico em estudo.**

|  |
| --- |

**6. PARA FINALIZAR, indicar**

País: Croácia / Estónia / Filândia / Alemanha / Israel / Lituânia / Portugal / Eslováquia / Espanha / Sérvia / Outro

Idade :____________

Sexo:  Homem  Mulher  Outro

Perfil profissional

Medicina

Enfermagem

Ciência Farmacêuticas

Neonatologia

Fisioterapia

Psicologia

Sociologia

Outro

Número de anos que realiza supervisão em contexto de estágio : _________

Número de estagiários que supervisionou ou realizou mentoria nos últimos 3 anos (2019-2021) : _____________________

Existe algum programa de formação específico em segurança do doente na sua instituição ?  Sim  Não

Selecione em que contexto realiza supervisão ou mentoria:  Sim  Não

Cuidados de saúde primários

Especialidade hospitalar

Assistência social

**SERBIAN VERSION**


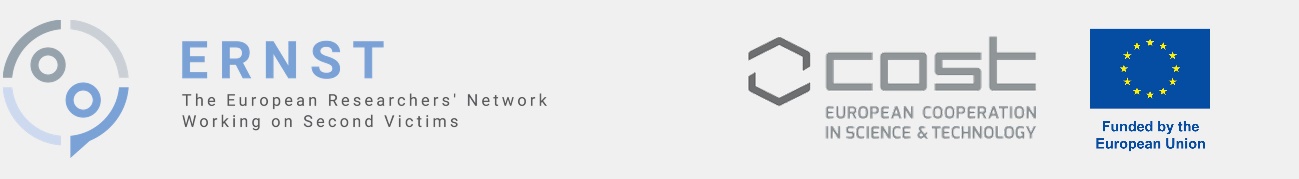


**Kompetencije studenata medicinskih nauka i stažera za promovisanje psihološke sigurnosti u zdravstvenim ustanovama**

Hvala Vam što ste pristali da učestvujete u ovom istraživanju koje se odnosi na sticanje kompetencije za psihološku sigurnost studenata medicinskih nauka i stažera tokom studentske prakse i staža.

Ovo istraživanje sprovode istraživači sa fakulteta i iz zdravstvenih ustanova, članovi evropskog projekta ERNST usmerenog na istraživanje bezbednosti pacijenata i "sekundarnih žrtava". Termin “sekundarne žrtve” odnosi se na zdravstvene radnike u situacijama kada je ugrožena bezbednost pacijenata, a kada i zdravstveni radnici trpe određene psihološke, socijalne, zdravstvene i druge posledice. U istraživanje su uključene kolege iz Hrvatske, Estonije, Finske, Nemačke, Izraela, Litvanije, Portugala, Slovačke, Španije i Srbije.

Pod psihološkom sigurnošću se podrazumeva uverenje da se tim koji zajedno radi može suočiti sa kompleksnim izazovima, a kada postoji atmosfera međusobnog poštovanja i poverenja. Dodatno, kada se u timu razgovara o uzrocima i načinima za izbegavanje kliničkih grešaka, tj. kada se greške analiziraju bez straha od kritika ili sankcija, zdravstveni radnici pružaju bezbedniju zdravstvenu zaštitu. Dakle, psihološka sigurnost zdravstvenih radnika može biti jedna od odrednica bezbednosti pacijenata.

Ovo Delfi istraživanje ispituje:

- koje kompetencije psihološke sigurnosti (tj. koja znanja, stavove i veštine) stiču buduće generacije zdravstvenih radnika (studenti i stažeri) kroz svoju studentsku prasku i staž,
- koje aktivnosti treba da implementira zdravstvena ustanova u kojoj student/stažer obavlja praksu kako bi promovisalo sticanje ovih kompetencija

Odgovori na upitnik biće analizirani na pseudoanoniman i zbirni način. Ne postoje tačni ili pogrešni odgovori na pitanja. Cilj je da saznamo Vašu percepciju kao mentora. Za popunjavanje upitnika je potrebno oko 10 minuta. Molimo Vas da ne zaboravite da pošaljete svoje odgovore na kraju upitnika.

U slučaju bilo kakvih dilema ili komentara, molimo Vas da pošaljite i-mejl svom nacionalnom koordinatoru (koji Vas je pozvao da učestvujete u istraživanju).

Unapred Vam se zahvaljujemo na saradnji i Vašim odgovorima.

**Informisani pristanak**

Učestvovanje u ovom istraživanju je dobrovoljno. Prikupljeni podaci će biti pseudo-anonimizirani i analizirani u zbirnom obilku. Platforma za prikupljanje podataka se nalazi na obezbeđenom serveru koji je smešten u Španiji i kojim upravlja Miguel Hernández Univerzitet, koji je deo španskog državnog obrazovnog sistema. Molimo Vas za potvrdu da učestvujete u ovom istraživanju dobrovoljno i da ste informisani o ciljevima i sadržaju istraživanju u iznad navedenom tekstu. U bilo kom trenutku možete da povučete svoj pristanak za učestvovanje u ovom istraživanju slanjem i-mejla na __________

Ja pristajem da učestvujem u ovom istraživanju na osnovu dobrovoljne i informisane saglasnosti.

**1. KOMPETENCIJE. Znanje**

Molimo Vas, procenite svaku kompetenciju (znanje) na osnovu dva kriterijuma:

*(1) Stepen sticanja (usvajanja) kompetencije*. Do kog stepena studenti/stažeri stiču ovu kompetenciju tokom prakse u Vašoj ustanovi?

1 Uopšte ne stiču – 5 U potpunosti stiču

*(2) Stepen značajnosti kompetencije.* Do kog stepena smatrate da ova kompetencija presudno doprinosi atmosferi psihološke sigurnosti u zdravstvenoj ustanovi?

1 Potpuno beznačajna – 5 Veoma značajna

| **Znanje. Prema mom mišljenju, praksa u mom radnom okruženju osposobljava studente/stažere da…** | **Stepen** | **1** | **2** | **3** | **4** | **5** |
| --- | --- | --- | --- | --- | --- | --- |
| - 1. … razumeju da otvoreno i direktno izražavanje zabrinutosti za bezbednost pacijenata može da spreči pojavu incidenata koji mogu da naškode pacijentu. | Sticanja |  |  |  |  |  |
|  | Značajnosti |  |  |  |  |  |
| - 1. … znaju kako da asertivno prenesu zabrinutost za bezbednost pacijenata drugom zdravstvenom radniku (istog ili višeg ranga) (npr. koje reči da izaberu, kako da započnu i završe razgovor, koji ton glasa ili gestikulaciju da koriste, itd.) | Sticanja |  |  |  |  |  |
|  | Značajnosti |  |  |  |  |  |
| - 1. … razlikuju situacije koje mogu da štete pacijentu, a koje su mogle da se izbegnu, od onih koje ne predstavljaju veliki rizik za bezbednost pacijenta. | Sticanja |  |  |  |  |  |
|  | Značajnosti |  |  |  |  |  |
| - 1. …izaberu najbolji trenutak za saopštavanje konkretne zabrinutosti za bezbednost pacijenta drugom zdravstvenom radniku (istog ili višeg nivoa). | Sticanja |  |  |  |  |  |
|  | Značajnosti |  |  |  |  |  |
| - 1. … znaju kako da asertivno upozore drugog zdravstvenog radnika (istog ili višeg ranga) na rizik od zanemarivanja važnog pravila za bezbednost pacijenata (reči koje treba da odaberu, kako da započnu i završe razgovor, koji ton glasa ili gestove da koriste itd. ). | Sticanja |  |  |  |  |  |
|  | Značajnosti |  |  |  |  |  |
| - 1. …znaju kako da se konstruktivno nose sa mogućom negativnom reakcijom zdravstvenog radnika (istog ili višeg ranga) nakon upozorenja da zanemaruje važno pravilo za bezbednost pacijenata. | Sticanja |  |  |  |  |  |
|  | Značajnosti |  |  |  |  |  |
| - 1. … znaju na koji način da izraze konkretne predloge koji bi mogli da poboljšaju bezbednost pacijenata u ustanovi. | Sticanja |  |  |  |  |  |
|  | Značajnosti |  |  |  |  |  |

**2. KOMPETENCIJE. Stavovi**

Molimo Vas, procenite svaku kompetenciju (stav) na osnovu dva kriterijuma:

*(1) Stepen sticanja (usvajanja) kompetencije*. Do kog stepena studenti/stažeri stiču ovu kompetenciju tokom prakse u Vašoj ustanovi?

1 Uopšte ne stiču – 5 U potpunosti stiču

*(2) Stepen značajnosti kompetencije.* Do kog stepena smatrate da ova kompetencija presudno doprinosi atmosferi psihološke sigurnosti u zdravstvenoj ustanovi?

1 Potpuno beznačajna – 5 Veoma značajna

| **Stav. Prema mom mišljenju, praksa u mom radnom okruženju osposobljava studente/stažere da…** | **Stepen** | **1** | **2** | **3** | **4** | **5** |
| --- | --- | --- | --- | --- | --- | --- |
| - 1. …se posvete prepoznavanju i prevenciji rizika za bezbednost pacijenata. | Sticanja |  |  |  |  |  |
|  | Značajnosti |  |  |  |  |  |
| - 1. … doživljavaju rizične situacije u svakodnevnom radu kao priliku za prepoznavanje rizika i preduzimanje odgovarajućih mere za sprečavanje štetnih posledica za pacijente. | Sticanja |  |  |  |  |  |
|  | Značajnosti |  |  |  |  |  |
| - 1. … pozitivno reaguju na upozorenja ili zabrinutost koje drugi zdravstveni radnici (istog ili višeg ranga) iznose u vezi sa bezbednošću pacijenata. | Sticanja |  |  |  |  |  |
|  | Značajnosti |  |  |  |  |  |
| - 1. … neguju pozitivan stav po pitanju upozorenja drugih zdravstvenih radnika u slučaju zanemarivanja važnog pravila značajnog za bezbednost pacijenta. | Sticanja |  |  |  |  |  |
|  | Značajnosti |  |  |  |  |  |
| - 1. … budu spremni da otvoreno i direktno dele konkretne predloge za poboljšanje bezbednosti pacijenata. | Sticanja |  |  |  |  |  |
|  | Značajnosti |  |  |  |  |  |
| - 1. … budu voljni da uče iz grešaka i incidenata u kojima su učestvovali drugi, umesto da ih osuđuju. | Sticanja |  |  |  |  |  |
|  | Značajnosti |  |  |  |  |  |

**3. KOMPETENCIJE. Veštine**

Molimo Vas, procenite svaku kompetenciju (veštinu) na osnovu dva kriterijuma:

*(1) Stepen sticanja (usvajanja) kompetencije*. Do kog stepena studenti/stažeri stiču ovu kompetenciju tokom prakse u Vašoj ustanovi?

1 Uopšte ne stiču – 5 U potpunosti stiču

*(2) Stepen značajnosti kompetencije.* Do kog stepena smatrate da ova kompetencija presudno doprinosi atmosferi psihološke sigurnosti u zdravstvenoj ustanovi?

1 Potpuno beznačajna – 5 Veoma značajna

| **Veštine. Prema mom mišljenju, praksa u mom radnom okruženju osposobljava studente/stažere da…** | **Stepen** | **1** | **2** | **3** | **4** | **5** |
| --- | --- | --- | --- | --- | --- | --- |
| - 1. … otvoreno i direktno komuniciraju sa drugim zdravstvenim radnicima (istog ili višeg ranga) o konkretnim problemima u vezi sa bezbednošću pacijenata pružanjem informacija, postavljanjem pitanja ili izražavanjem mišljenja. | Sticanja |  |  |  |  |  |
|  | Značajnosti |  |  |  |  |  |
| - 1. … zatraže savet od odgovornih zdravstvenih radnika da prijave incident koji se odnosi na bezbednost pacijenta kome je bio svedok i da sačini izveštaj/prijavu (ukoliko je potrebno). | Sticanja |  |  |  |  |  |
|  | Značajnosti |  |  |  |  |  |
| - 1. … odlučno upozori drugog zdravstvenog radnika (istog ili višeg ranga) da svojim postupcima zanemaruje važno pravilo vezano za bezbednost pacijenata. | Sticanja |  |  |  |  |  |
|  | Značajnosti |  |  |  |  |  |
| - 1. … odgovore asertivno na negativnu reakciju zdravstvenog radnika (istog ili višeg ranga) koga je upozorio da zanemaruje važno pravilo vezano za bezbednost pacijenta. | Sticanja |  |  |  |  |  |
|  | Značajnosti |  |  |  |  |  |
| - 1. … verbalno podrže i osnaže inicijativu drugih zdravstvenih radnika (istog ili višeg ranga) da sa ostatkom tima dele svoje konkretne brige o bezbednosti pacijenata. | Sticanja |  |  |  |  |  |
|  | Značajnosti |  |  |  |  |  |
| - 1. … osmisle i saopšte konkretne predloge za poboljšanje bezbednosti pacijenata u sopstvenoj ustanovi. | Sticanja |  |  |  |  |  |
|  | Značajnosti |  |  |  |  |  |
| - 1. …ponude podršku kolegi koji je uključen u neželjeni događaj kako bi se ublažio sindrom "sekundarne žrtve" (okarakterisan osećanjem krivice, nedovoljne kompetentnosti, anksioznosti, stida, preteranog opreza ili tuge). | Sticanja |  |  |  |  |  |
|  | Značajnosti |  |  |  |  |  |

**4. INTERVENCIIJE**

Molimo Vas, procenite svaku intervenciju na osnovu dva kriterijuma:

*(1) Stepen sticanja (usvajanja) kompetencije*. Do kog stepena studenti/stažeri stiču ovu kompetenciju tokom prakse u Vašoj ustanovi?

1 Uopšte ne stiču – 5 U potpunosti stiču

*(2) Stepen značajnosti kompetencije.* Do kog stepena smatrate da ova kompetencija presudno doprinosi atmosferi psihološke sigurnosti u zdravstvenoj ustanovi?

1 Potpuno beznačajna – 5 Veoma značajna

| **Intervencije. Moja zdravstvena ustanova…** | **Stepen** | **1** | **2** | **3** | **4** | **5** |
| --- | --- | --- | --- | --- | --- | --- |
| - 1. …sprovodi program obuke za novo osoblje (naročito za studente/stažere) kako bi se podstao razvoj pozitivne atmosfere za bezbednost pacijenata i psihološku sigurnost zaposlenih. | Implementacije |  |  |  |  |  |
|  | Značajnosti |  |  |  |  |  |
| - 1. …imenuje grupu ljudi od uticaja kako bi osmislili plan intervencija za formiranje i negovanje atmosfere poverenja među zdravstvenim radnicima čime bi se dodatno osigurala bezbednost pacijenta. | Implementacije |  |  |  |  |  |
|  | Značajnosti |  |  |  |  |  |
| - 1. …održava redovne kliničke sastanke sa studentima/stažerima kako bi se diskutovali slučajevi značajni za bezbednost pacijenta i naučene lekcije. Zahvaljujući ovoj intervenciji, brojna iskustva u vezi se bezbednošču pacijenata se razmenjuju na jednom mestu uz istovremeno osmišljavanje rešenja za smanjenje rizika i pružanje međusobne emocionalne podrške studentima/stažerima. | Implementacije |  |  |  |  |  |
|  | Značajnosti |  |  |  |  |  |
| - 1. …podiže svest među zdravstvenim radnicima o potrebi da se ohrabre studenti/stažeri i kolege da otvoreno i direktno izraze zabrinutost u vezi sa bezbednošću pacijenata i upozore druge na rizike koje identifikuju u svom svakodnevnom radu. | Implementacije |  |  |  |  |  |
|  | Značajnosti |  |  |  |  |  |
| - 1. …podiže svest među zdravstvenim radnicima o važnosti pozitivnog reagovanja na upozorenja u vezi sa poštovanjem pravila vezanih za bezbednost pacijenata i jačanja otvorenog izražavanja konkretnih zabrinutosti za bezbednost pacijenata od strane studenata/stažera. | Implementacije |  |  |  |  |  |
|  | Značajnosti |  |  |  |  |  |
| - 1. … pruža studentima/stažerima priliku da učestvuju kao posmatrači tokom razgovora o neželjenom događaju sa porodicom pacijenta kome se događaj dogodio. | Implementacije |  |  |  |  |  |
|  | Značajnosti |  |  |  |  |  |
| - 1. …omogućava studentima/stažerima da budu prisutni tokom diskusije i analize incidenta koji se dogodio, a vezan je za bezbednost pacijenta. | Implementacije |  |  |  |  |  |
|  | Značajnosti |  |  |  |  |  |
| - 1. …pruža studentima/stažerima specifičnu obuku u vezi sa prijavljivanjem incidenata koji se odnose na bezbednost pacijenata. | Implementacije |  |  |  |  |  |
|  | Značajnosti |  |  |  |  |  |
| - 1. …nudi institucionalnu podršku zdravstvenim radnicima koji su uključeni u neželjeni događaj kako bi doprineli boljoj bezbednosti na radnom mestu. | Implementacije |  |  |  |  |  |
|  | Značajnosti |  |  |  |  |  |

**5. UKOLIKO ŽELITE, možete koristiti ovaj prostor da dodate nešto što smatrate da nedostaje u upitniku ili nešto što smatrate adekvatnom napomenom, a u vezi je sa temom o kojoj se govori.**

|  |
| --- |

**6. NA KRAJU, molimo Vas da označite**

Država: Hrvatska / Estonija / Finska / Nemačka / Izrael / Litvanija / Portugal / Slovačka / Španija / Serbia / Drugo

Godine: _____________

Pol:  Muški  Ženski  Drugo

Profesija:

Medicina

Sestrinstvo

Farmacija

Akušerstvo

Fizioterapija

Psihologija

Sociologija

Drugo

Koliko godina ste do sad bili odgovorni za student/stažere: _________

Broj studenata/stažera koje ste lično nadgledali ili mentorisali u poslednje tri godine (2019-2021): _________

Da li u Vašoj ustanovi postoji poseban program obuke o bezbednosti pacijenata?  Da  Ne

Okruženje gde radite je:

Na primarnom nivou zdravstvene zaštite

Specijalna bolnica

Ustanova socijalne zaštite

**SLOVAK VERSION**


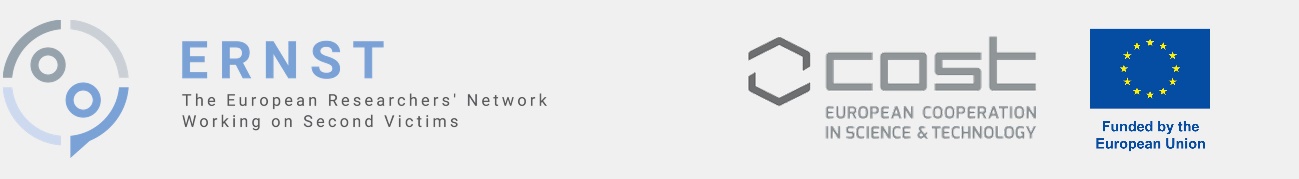


**Kompetencie zdravotníkov a zdravotníčok pripravujúcich sa na výkon povolania potrebné pre podporu psychologicky bezpečnej atmosféry v klinickej praxi**

Ďakujeme, že ste súhlasili zúčastniť sa na tejto štúdii o kompetenciách v oblasti psychologickej bezpečnosti, ktoré získavajú stážisti (pregraduálni študenti a študenti špecializačného štúdia) zdravotníckych odborov počas obdobia svojej odbornej prípravy.

Sme tím výskumníkov z akademickej a klinickej oblasti, ktorý je súčasťou Európskeho konzorcia ERNST zameraného na štúdie o bezpečnosti pacientov a sekundárne obete. Táto štúdia zahŕňa výskumníkov z Chorvátska, Estónska, Fínska, Nemecka, Izraela, Litvy, Portugalska, Slovenska, Španielska a Srbsko.

Psychologická bezpečnosť sa týka zdieľaného presvedčenia, že pracovný tím dokáže čeliť komplexným výzvam ak existuje atmosféra vzájomného rešpektu a dôvery. Zdravotnícki pracovníci poskytujú bezpečnejšiu starostlivosť vtedy, keď príčiny a spôsoby, ako sa vyhnúť klinickým chybám, môžu byť diskutované a analyzované bez strachu z kritiky alebo sankcií. Klíma psychologickej bezpečnosti môže byť rozhodujúcim faktorom bezpečnosti pacienta. Týmto spôsobom je poskytovaná bezpečnejšia starostlivosť, keď je možné diskutovať a analyzovať príčiny a mechanizmy, ako zabrániť klinickým chybám bez strachu z kritiky alebo sankcií.

Táto štúdia Delphi skúma:

- aké kompetencie v oblasti psychologickej bezpečnosti (vedomosti, postoje a zručnosti) získavajú budúce generácie zdravotníckych pracovníkov (študenti a rezidenti, ďalej len "stážisti") prostredníctvom svojich klinických stáží,
- aké opatrenia by mali zaviesť zdravotnícke zariadenia, v ktorých stážisti absolvujú svoje klinické stáže, na podporu získania týchto kompetencií.

Odpovede respondentov budeme spracúvať spôsobom, ktorý zaručí anonymitu poskytovateľa informácie. Našou snahou je spoznať Váš pohľad ako mentora/ky stážistov pripravujúcich sa na výkon zdravotníckeho povolania a neexistujú žiadne správne alebo nesprávne odpovede na položené otázky. Vyplnenie dotazníka bude trvať približne 10 minút. Nezabudnite svoje odpovede na konci dotazníka odoslať.

V prípade akýchkoľvek otázok alebo pripomienok pošlite e-mail svojej národnej koordinátorke (ktorá vás pozvala zúčastniť sa štúdie).

Vopred vám ďakujeme za spoluprácu a vaše odpovede.

**Informovaný súhlas**

Účasť na tejto štúdii je dobrovoľná. Odpovede respondentov budeme spracúvať spôsobom, ktorý zaručí anonymitu poskytovateľa informácie. Platforma je uložená na bezpečnom serveri umiestnenom v Španielsku a spravovaná Univerzitou Miguela Hernándeza, ktorá je súčasťou španielskeho verejného vzdelávacieho systému. Ak súhlasíte s účasťou na tejto štúdii, prosím potvrďte, že sa jej zúčastňujete dobrovoľne a informovane. Svoj súhlas participovať na štúdii môžete kedykoľvek odvolať zaslaním e-mailu na ___________

Dobrovoľne a informovane súhlasím s účasťou na štúdii.

**1. KOMPETENCIE. Poznatky**

Prosím, posúďte každú kompetenciu (poznatok) podľa dvoch kritérií:

*(1) Stupeň nadobudnutia*. Do akej miery stážisti v súčasnosti získavajú túto kompetenciu prostredníctvom svojich klinických stáží vo vašej inštitúcii?

1 Nenadobúdajú vôbec – 5 Plne nadobúdajú

*(2) Stupeň dôležitosti.* Do akej miery sa domnievate, že táto kompetencia rozhodujúcim spôsobom prispieva ku klíme psychologickej bezpečnosti v zdravotníckom zariadení?

1 Vôbec nie je dôležitá – 5 Veľmi dôležitá

| **Poznatky. Podľa môjho názoru stáže v mojom pracovnom prostredí poskytujú stážistom kompetencie...** | **Stupeň** | **1** | **2** | **3** | **4** | **5** |
| --- | --- | --- | --- | --- | --- | --- |
| - 1. …pochopiť, že otvorené a priame vyjadrenie obáv o bezpečnosť pacienta môže zabrániť výskytu incidentov, ktoré by mohli pacientovi ubížiť. | Nadobudnutia |  |  |  |  |  |
|  | Dôležitosti |  |  |  |  |  |
| - 1. …vedieť, ako asertívne komunikovať obavy o bezpečnosť pacientov inému zdravotníckemu pracovníkovi (na rovnakej alebo vyššej úrovni) (aké slová si vybrať, ako začať a dokončiť konverzáciu, aký tón hlasu alebo gestá použiť, atď.). | Nadobudnutia |  |  |  |  |  |
|  | Dôležitosti |  |  |  |  |  |
| - 1. …rozlišovať medzi situáciami, ktoré by mohli spôsobiť ujmu pacientovi od tých, ktoré nepredstavujú vysoké riziko pre bezpečnosť pacienta. | Nadobudnutia |  |  |  |  |  |
|  | Dôležitosti |  |  |  |  |  |
| - 1. …vybrať si najlepší moment na to, aby komunikovali konkrétne obavy o bezpečnosť pacienta inému zdravotníckemu pracovníkovi (na rovnakej úrovni alebo vyššej). | Nadobudnutia |  |  |  |  |  |
|  | Dôležitosti |  |  |  |  |  |
| - 1. …vedieť asertívne upozorniť iného zdravotníckeho pracovníka (na rovnakej úrovni alebo vyššej) na riziko ignorovania dôležitého pravidla bezpečnosti pacienta (výber slov, ako začať a dokončiť konverzáciu, aký tón hlasu alebo gestá použiť, atď.) | Nadobudnutia |  |  |  |  |  |
|  | Dôležitosti |  |  |  |  |  |
| - 1. … vedieť sa konštruktívne vysporiadať s možnou negatívnou reakciou zdravotníckeho pracovníka (na rovnakej alebo vyššej úrovni) po upozornení, že prehliada dôležité pravidlo pre bezpečnosť pacienta. | Nadobudnutia |  |  |  |  |  |
|  | Dôležitosti |  |  |  |  |  |
| - 1. …vedieť, ako vyjadriť konkrétne návrhy, ktoré by mohli zlepšiť bezpečnosť pacientov na oddelení. | Nadobudnutia |  |  |  |  |  |
|  | Dôležitosti |  |  |  |  |  |

**2. KOMPETENCIE. Postoje**

Prosím, posúďte každú kompetenciu (postoj) podľa dvoch kritérií:

*(1) Stupeň nadobudnutia*. Do akej miery stážisti v súčasnosti získavajú túto kompetenciu prostredníctvom svojich klinických stáží vo vašej inštitúcii?

1 Nenadobúdajú vôbec – 5 Plne nadobúdajú

*(2) Stupeň dôležitosti.* Do akej miery sa domnievate, že táto kompetencia rozhodujúcim spôsobom prispieva ku klíme psychologickej bezpečnosti v zdravotníckom zariadení?

1 Vôbec nie je dôležitá – 5 Veľmi dôležitá

| **Postoj. Podľa môjho názoru stáže v mojom pracovnom prostredí poskytujú stážistom kompetencie...** | **Stupeň** | **1** | **2** | **3** | **4** | **5** |
| --- | --- | --- | --- | --- | --- | --- |
| - 1. …zaviazať sa k identifikovaniu a prevencii rizík pre bezpečnosť pacienta. | Nadobudnutia |  |  |  |  |  |
|  | Dôležitosti |  |  |  |  |  |
| - 1. …vnímať rizikové situácie v každodennej práci ako príležitosť zdôrazniť riziko a prijať vhodné opatrenia na zabránenie poškodenia pacientov. | Nadobudnutia |  |  |  |  |  |
|  | Dôležitosti |  |  |  |  |  |
| - 1. …pozitívne reagovať na vyjadrenie upozornení alebo obáv, ktoré uskutočnia iní zdravotnícki pracovníci (na rovnakej úrovni alebo vyššej) vo vzťahu k bezpečnosti pacientov. | Nadobudnutia |  |  |  |  |  |
|  | Dôležitosti |  |  |  |  |  |
| - 1. … zachovať si pozitívny prístup k upozorňovaniu ostatných zdravotníckych pracovníkov, ak svojim konaním ignorujú dôležité pravidlo pre bezpečnosť pacienta. | Nadobudnutia |  |  |  |  |  |
|  | Dôležitosti |  |  |  |  |  |
| - 1. … byť ochotní otvorene a priamo zdieľať konkrétne návrhy na zlepšenie bezpečnosti pacienta. | Nadobudnutia |  |  |  |  |  |
|  | Dôležitosti |  |  |  |  |  |
| - 1. …byť ochotní poučiť sa z chýb a bezpečnostných incidentov, do ktorých boli zapojení iní odborníci, namiesto toho, aby ich odsudzovali. | Nadobudnutia |  |  |  |  |  |
|  | Dôležitosti |  |  |  |  |  |

**3. KOMPETENCIE. Zručnosti**

Prosím, posúďte každú kompetenciu (zručnosť) podľa dvoch kritérií:

*(1) Stupeň nadobudnutia*. Do akej miery stážisti v súčasnosti získavajú túto kompetenciu prostredníctvom svojich klinických stáží vo vašej inštitúcii?

1 Nenadobúdajú vôbec – 5 Plne nadobúdajú

*(2) Stupeň dôležitosti.* Do akej miery sa domnievate, že táto kompetencia rozhodujúcim spôsobom prispieva ku klíme psychologickej bezpečnosti v zdravotníckom zariadení?

1 Vôbec nie je dôležitá – 5 Veľmi dôležitá

| **Zručnosti. Podľa môjho názoru stáže v mojom pracovnom prostredí poskytujú stážistom kompetencie...** | **Stupeň** | **1** | **2** | **3** | **4** | **5** |
| --- | --- | --- | --- | --- | --- | --- |
| - 1. …otvorene a priamo komunikovať s inými odborníkmi (na rovnakej alebo vyššej úrovni) konkrétne obavy o bezpečnosť pacientov tým, že prezentujú informácie, kladú otázky alebo vyjadrujú názory. | Nadobudnutia |  |  |  |  |  |
|  | Dôležitosti |  |  |  |  |  |
| - 1. … požiadať o radu zodpovedného pracovníka ako nahlásiť v príslušnom systéme výskyt udalosti ohrozujúcej bezpečnosť pacienta, ktorá sa udiala a podať o tom správu (ak je to potrebné). | Nadobudnutia |  |  |  |  |  |
|  | Dôležitosti |  |  |  |  |  |
| - 1. …asertívne varovať iného zdravotníckeho pracovníka (na rovnakej alebo vyššej úrovni), že svojim konaním ignoruje dôležité pravidlo bezpečnosti pacienta. | Nadobudnutia |  |  |  |  |  |
|  | Dôležitosti |  |  |  |  |  |
| - 1. …asertívne reagovať na negatívnu reakciu zdravotníckeho pracovníka (rovnakej úrovne alebo vyššej), ktorého varovali pred ignorovaním dôležitého pravidla bezpečnosti pacienta. | Nadobudnutia |  |  |  |  |  |
|  | Dôležitosti |  |  |  |  |  |
| - 1. …verbálne podporiť a posilniť iniciatívu iných zdravotníckych pracovníkov (rovnakej alebo vyššej úrovne), aby zdieľali svoje špecifické obavy o bezpečnosť pacientov so zvyškom tímu. | Nadobudnutia |  |  |  |  |  |
|  | Dôležitosti |  |  |  |  |  |
| - 1. …stanoviť a komunikovať konkrétne návrhy na zlepšenie bezpečnosti pacienta na vlastnom oddelení alebo službe. | Nadobudnutia |  |  |  |  |  |
|  | Dôležitosti |  |  |  |  |  |
| - 1. …ponúknuť podporu kolegovi/kolegyni účastnej nežiaducej udalosti pri poskytovaní zdravotníckej starostlivosti, aby sa znížilo riziko rozvinutia syndrómu sekundárnej obete (charakterizované pocitmi viny, nedostatočnosti, úzkosťou, hanbou, nadmernou ostražitosťou alebo smútkom). | Nadobudnutia |  |  |  |  |  |
|  | Dôležitosti |  |  |  |  |  |

**4. INTERVENCIE**

Prosím, posúďte každú intervenciu podľa dvoch kritérií:

*(1) Stupeň implementácie.* Do akej miery sa táto intervencia vykonáva vo vašom bezprostrednom klinickom prostredí?

1 Zatiaľ neimplementovaná – 5 Plne implementovaná

*(2) Stupeň dôležitosti.* Do akej miery sa domnievate, že táto kompetencia rozhodujúcim spôsobom prispieva ku klíme psychologickej bezpečnosti v zdravotníckom zariadení?

1 Vôbec nie je dôležitá – 5 Veľmi dôležitá

| **Intervencie. Moja inštitúcia v oblasti zdravotnej starostlivosti…** | **Stupeň** | **1** | **2** | **3** | **4** | **5** |
| --- | --- | --- | --- | --- | --- | --- |
| - 1. … implementuje vzdelávací program pre nových zamestnancov (najmä stážistov) na podporu bezpečnosti pacientov a podporu atmosféry psychologickej bezpečnosti pre zamestnancov. | Implementácie |  |  |  |  |  |
|  | Dôležitosti |  |  |  |  |  |
| - 1. … nominuje vplyvnú skupinu ľudí, aby navrhla intervenčný plán na podporu atmosféry dôvery medzi zdravotníckymi pracovníkmi s cieľom zaistiť bezpečnosť pacientov. | Implementácie |  |  |  |  |  |
|  | Dôležitosti |  |  |  |  |  |
| - 1. …organizuje pravidelné klinické stretnutia so stážistami, aby zdieľali obavy týkajúce sa bezpečnosti pacientov a získané poznatky. Toto opatrenie sa premieta do vytvorenia zdieľaného priestoru na výmenu skúseností s udalosťami ohrozujúcimi bezpečnosť pacientov, navrhovanie bariér na minimalizáciu rizík a poskytovanie emocionálnej a inštrumentálnej podpory medzi kolegami. | Implementácie |  |  |  |  |  |
|  | Dôležitosti |  |  |  |  |  |
| - 1. … zvyšuje povedomie zdravotníckych pracovníkov danej inštitúcie, v spolupráci s manažérmi zodpovednými za poskytovanie starostlivosti o potrebe povzbudiť stážistov a kolegov, aby otvorene a priamo vyjadrili svoje obavy, týkajúce sa bezpečnosti pacientov a varovali ostatných odborníkov pred rizikami, ktoré identifikujú vo svojej každodennej práci. | Implementácie |  |  |  |  |  |
|  | Dôležitosti |  |  |  |  |  |
| - 1. …zvyšuje povedomie zdravotníckych pracovníkov danej inštitúcie, v spolupráci s manažermi zodpovednými za poskytovanie starostlivosti o dôležitosti pozitívnej reakcie na varovania iných odborníkov, pokiaľ ide o dodržiavanie príslušných pravidiel bezpečnosti pacientov a posilnenie otvoreného vyjadrovania konkrétnych obáv o bezpečnosť pacientov zo strany stážistov. | Implementácie |  |  |  |  |  |
|  | Dôležitosti |  |  |  |  |  |
| - 1. … poskytuje stážistom možnosť zúčastniť sa v roli pozorovateľov pri plánovaní rozhovorov s cieľom informovať pacientov a ich blízkych o nežiadúcej udalosti. | Implementácie |  |  |  |  |  |
|  | Dôležitosti |  |  |  |  |  |
| - 1. …umožňuje stážistom byť prítomní počas diskusie a analýzy, ktorá nasleduje po udalosti ohrozujúcej bezpečnosť pacienta. | Implementácie |  |  |  |  |  |
|  | Dôležitosti |  |  |  |  |  |
| - 1. …poskytuje stážistom osobitné školenie o nahlasovaní chýb pri poskytovaní zdravotníckej starostlivosti vhodnými prostriedkami. | Implementácie |  |  |  |  |  |
|  | Dôležitosti |  |  |  |  |  |
| - 1. …ponúka inštitucionálnu podporu zdravotníckym pracovníkom zapojeným do nežiaducej udalosti s cieľom prispieť k lepšej bezpečnosti na pracovisku. | Implementácie |  |  |  |  |  |
|  | Dôležitosti |  |  |  |  |  |

1. **AK SI ŽELÁTE, použite tento priestor na pridanie niečoho, čo ste vynechali v dotazníku, alebo čokoľvek, čo považujete za vhodné poznamenať v súvislosti s riešenou témou.**

|  |
| --- |

1. **NA ZÁVER, prosím uveďte**

Krajina: Chorvátsko / Estónsko / Fínsko / Nemecko / Izrael / Litva / Portugalsko / Slovensko / Španielsko / Srbsko / Iná

Vek: _____________

Pohlavie:  Muž  Žena  Iné

Profesionálny profil:

Medicína

Ošetrovateľstvo

Farmácia

Pôrodníctvo

Fyzioterapia

Psychológia

Sociológia

Iné

Roky zodpovednosti za stážistov: _________

Počet stážistov, ktoré ste osobne supervidovali alebo mentorovali za posledné tri roky (2019-2021): _________

Existuje špecifický tréningový program k bezpečnosti pacienta vo vašom centre?

Prostredie kde vykonávate svoju klinickú a mentorskú prácu:

Primárna starostlivosť

Špecializovaná starostlivosť (nemocnica)

Sociálna starostlivosť

**SPANISH VERSION**


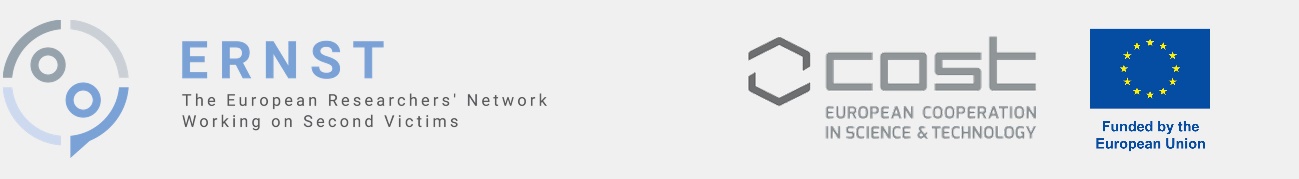


**Competencias en estudiantes y residentes de disciplinas sanitaria para promover un clima de seguridad psicológica en los contextos clínicos**

Gracias por aceptar participar en este estudio sobre competencias en seguridad psicológica adquiridas por residentes y estudiantes de disciplinas sanitarias durante su periodo en formación.

Somos un equipo de investigadores del ámbito clínico y académico que pertenecemos al Consorcio Europeo ERNST cuyo propósito es la realización de estudios sobre seguridad del paciente y segundas víctimas. En este estudio en particular participan investigadores de Alemania, Croacia, Eslovaquia, España, Estonia, Finlandia, Israel, Lituania, Malta, Portugal y Serbia.

La seguridad psicológica hace referencia a la creencia compartida de que un equipo de trabajo es capaz de afrontar retos complejos cuando existe un ambiente de respeto mutuo y confianza. De este modo, se proporciona una asistencia más segura cuando se puede hablar y analizar las causas y modo de evitar los errores clínicos sin temor a críticas o sanciones. Un clima de seguridad psicológica puede ser un determinante de la seguridad del paciente. De este modo, se consigue una atención más segura cuando es posible discutir y analizar las causas y los mecanismos para evitar los errores clínicos sin temor a las críticas ni al castigo.

Este estudio Delphi explora:

- qué competencias en seguridad psicológica (conocimientos, actitudes y habilidades) están siendo adquiridas por las futuras generaciones de profesionales sanitarios (estudiantes y residentes) durante su periodo de residencia o prácticas en centros sanitarios,
- qué acciones deberían implementar las instituciones sanitarias en las que estudiantes y residentes realizan sus prácticas para promover la adquisición de estas competencias.

Las respuestas al cuestionario serán analizadas de forma pseudoanonimizada y agregada. No hay respuestas correctas ni incorrectas, sino que se pretende conocer su percepción como tutor/a de residentes o estudiantes en prácticas. La cumplimentación del cuestionario requiere aproximadamente 10 minutos. Por favor, no olvide enviar sus respuestas al finalizar el cuestionario.

Para cualquier cuestión o comentario, por favor envíe un email a su coordinador nacional (quien le invitó a participar en el estudio).

Gracias de antemano por su colaboración y respuestas.

**Consentimiento informado**

La participación en este estudio es voluntaria. Los datos recogidos serán anonimizados y analizados de forma agregada. La plataforma web está alojada en un servidor seguro localizado en España y gestionado por la Universidad Miguel Hernández de Elche, que forma parte del sistema educativo público español. Por favor, confirme que su participación en este estudio es voluntaria e informada. Puede revocar su consentimiento a participar en el estudio en cualquier momento enviando un email a __________

Consiento participar en el estudio de forma voluntaria e informada.

**2. COMPETENCIAS. Conocimientos**

Por favor, valore cada competencia (conocimiento) atendiendo a los siguientes criterios:

*(1) Grado de adquisición*. ¿En qué medida están adquiriendo esta competencia los estudiantes y residentes que están realizando sus prácticas o periodo de especialización en su institución sanitaria?

1 No adquirida, en absoluto – 5 Completamente adquirida

*(2) Grado de importancia*. ¿En qué medida considera que esta competencia contribuye de manera decisiva a la creación de un clima de seguridad psicológica en los entornos clínicos?

1 Nada importante – 5 Muy importante

| **Conocimiento. En mi opinión, la formación que estudiantes y residentes reciben en mi centro de trabajo promueve la adquisición de la competencia para:** | **Grado de** | **1** | **2** | **3** | **4** | **5** |
| --- | --- | --- | --- | --- | --- | --- |
| 1.1. ... comprender que la expresión abierta y directa de preocupaciones sobre la seguridad de los pacientes puede prevenir la ocurrencia de incidentes que podrían ocasionar algún daño al paciente. | Adquisición |  |  |  |  |  |
|  | Importancia |  |  |  |  |  |
| 1.2. …saber cómo comunicar de forma asertiva una preocupación relativa a la seguridad de los pacientes a otro profesional sanitario (de igual nivel o superior) (qué palabras escoger, cómo iniciar y concluir la conversación, qué tono de voz o gestualidad emplear, etc.). | Adquisición |  |  |  |  |  |
|  | Importancia |  |  |  |  |  |
| 1.3. …distinguir las situaciones que podrían ocasionar un daño evitable al paciente de aquellas que no representan un riesgo elevado para la seguridad de pacientes. | Adquisición |  |  |  |  |  |
|  | Importancia |  |  |  |  |  |
| 1.4. …escoger el momento oportuno para comunicar preocupaciones específicas sobre la seguridad de los pacientes a otro profesional sanitario (de igual nivel o superior). | Adquisición |  |  |  |  |  |
|  | Importancia |  |  |  |  |  |
| 1.5. …saber cómo advertir de forma asertiva a otro profesional sanitario (de igual nivel o superior) del riesgo de ignorar una norma de importante cumplimiento para la seguridad de los pacientes (qué palabras escoger, cómo iniciar y concluir la conversación, qué tono de voz o gestualidad emplear, etc.). | Adquisición |  |  |  |  |  |
|  | Importancia |  |  |  |  |  |
| 1.6. … conocer cómo afrontar de manera constructiva la posible reacción negativa de un profesional sanitario (de igual nivel o superior) tras haberle advertido de que estaba pasando por alto una norma de cumplimiento importante para la seguridad de pacientes. | Adquisición |  |  |  |  |  |
|  | Importancia |  |  |  |  |  |
| 1.7. …saber cómo expresar propuestas específicas que podrían mejorar la seguridad de los pacientes en la unidad o servicio de adscripción. | Adquisición |  |  |  |  |  |
|  | Importancia |  |  |  |  |  |

**2. COMPETENCIAS. Actitudes**

Por favor, valore cada competencia (actitud) atendiendo a los siguientes criterios:

*(1) Grado de adquisición*. ¿En qué medida están adquiriendo esta competencia los estudiantes y residentes que están realizando sus prácticas o periodo de especialización en su institución sanitaria?

1 No adquirida, en absoluto – 5 Completamente adquirida

*(2) Grado de importancia*. ¿En qué medida considera que esta competencia contribuye de manera decisiva a la creación de un clima de seguridad psicológica en los entornos clínicos?

1 Nada importante – 5 Muy importante

| **Actitud. En mi opinión, la formación que estudiantes y residentes reciben en mi centro de trabajo promueve la adquisición de la competencia para:** | **Grado de** | **1** | **2** | **3** | **4** | **5** |
| --- | --- | --- | --- | --- | --- | --- |
| 2.1. …comprometerse con la identificación y prevención de riesgos para la seguridad de los pacientes. | Adquisición |  |  |  |  |  |
|  | Importancia |  |  |  |  |  |
| 2.2. …percibir las situaciones de riesgo que se presentan en su quehacer diario como oportunidades para evidenciar el riesgo y adoptar medidas oportunas que eviten que los pacientes sufran algún daño. | Adquisición |  |  |  |  |  |
|  | Importancia |  |  |  |  |  |
| 2.3. …responder de forma positiva ante las advertencias o preocupaciones de otros profesionales sanitarios (de igual nivel o superior) en relación con la seguridad de los pacientes. | Adquisición |  |  |  |  |  |
|  | Importancia |  |  |  |  |  |
| 2.4. …mantener una actitud favorable hacia la práctica de advertir a otros profesionales sanitarios del riesgo asociado a su comportamiento en los casos en los que estén pasando por alto una norma de importante cumplimiento para la seguridad de los pacientes. | Adquisición |  |  |  |  |  |
|  | Importancia |  |  |  |  |  |
| 2.5. …estar dispuesto/a a compartir de manera abierta y directa propuestas específicas para mejorar la seguridad de los pacientes. | Adquisición |  |  |  |  |  |
|  | Importancia |  |  |  |  |  |
| 2.6 …estar dispuesto a aprender de los errores e incidentes de seguridad en los que otros profesionales se hayan visto implicados, en lugar de juzgarles | Adquisición |  |  |  |  |  |
|  | Importancia |  |  |  |  |  |

**3. COMPETENCIAS. Habilidades**

Por favor, valore cada competencia (habilidad) atendiendo a los siguientes criterios:

*(1) Grado de adquisición*. ¿En qué medida están adquiriendo esta competencia los estudiantes y residentes que están realizando sus prácticas o periodo de especialización en su institución sanitaria?

1 No adquirida, en absoluto – 5 Completamente adquirida

*(2) Grado de importancia*. ¿En qué medida considera que esta competencia contribuye de manera decisiva a la creación de un clima de seguridad psicológica en los entornos clínicos?

1 Nada importante – 5 Muy importante

| **Habilidades. En mi opinión, la formación que estudiantes y residentes reciben en mi centro de trabajo promueve la adquisición de la competencia para:** | **Grado de** | **1** | **2** | **3** | **4** | **5** |
| --- | --- | --- | --- | --- | --- | --- |
| 3.1 …comunicar de manera abierta y directa a otros profesionales (de igual nivel o superior) preocupaciones específicas sobre la seguridad de los pacientes mediante la presentación de información, la formulación de preguntas o la expresión de opiniones. | Adquisición |  |  |  |  |  |
|  | Importancia |  |  |  |  |  |
| 3.2 … solicitar al profesional responsable (en caso necesario), asesoramiento para notificar, en el sistema oportuno, la ocurrencia de un incidente de seguridad del paciente del que se ha sido testigo y efectuar la notificación. | Adquisición |  |  |  |  |  |
|  | Importancia |  |  |  |  |  |
| 3.3 …advertir de forma asertiva a otro profesional sanitario (de igual nivel o superior) de que con su actuación está pasando por alto una norma de importante cumplimiento para la seguridad de los pacientes. | Adquisición |  |  |  |  |  |
|  | Importancia |  |  |  |  |  |
| 3.4. …responder de manera asertiva a la reacción negativa de un profesional sanitario (de igual nivel o superior) tras haberle advertido de que estaba pasando por alto una norma de cumplimiento importante para la seguridad de los pacientes. | Adquisición |  |  |  |  |  |
|  | Importancia |  |  |  |  |  |
| 3.5 …apoyar y reforzar verbalmente la iniciativa de otros profesionales sanitarios (de igual nivel o superior) de compartir con el resto del equipo sus preocupaciones específicas sobre la seguridad de los pacientes. | Adquisición |  |  |  |  |  |
|  | Importancia |  |  |  |  |  |
| 3.6 …idear y comunicar propuestas concretas para mejorar la seguridad de los pacientes en el propio servicio o unidad. | Adquisición |  |  |  |  |  |
|  | Importancia |  |  |  |  |  |
| 3.7 …ofrecer apoyo a un colega implicado en un evento adverso para reducir el síndrome de la segunda víctima (caracterizado por sentimientos de culpa, inadecuación, ansiedad, vergüenza, hipervigilancia o tristeza). | Adquisición |  |  |  |  |  |
|  | Importancia |  |  |  |  |  |

**4. INTERVENCIONES**

Por favor, valore cada intervención atendiendo a los siguientes criterios:

*(1) Grado de implantación*. ¿En qué medida la intervención descrita está implantada en su entorno clínico más inmediato?

1 No implantada – 5 Completamente implantada

*(2) Grado de importancia*. ¿En qué medida considera que esta intervención contribuye de manera decisiva a la creación de un clima de seguridad psicológica en los entornos clínicos?

1 Nada importante – 5 Muy importante

| **Intervenciones. Mi institución sanitaria…** | **Grado de** | **1** | **2** | **3** | **4** | **5** |
| --- | --- | --- | --- | --- | --- | --- |
| 4.1 …implantar un programa de formación para el personal de nuevo ingreso (especialmente estudiantes y residentes) dirigido a fomentar una cultura positiva de seguridad del paciente y de un clima de seguridad psicológica. | Implantación |  |  |  |  |  |
|  | Importancia |  |  |  |  |  |
| 4.2 … designar a un grupo de personas que sean influyentes en la organización para diseñar un plan de intervención que fomente un clima de confianza entre los profesionales de la salud para asegurar la seguridad del paciente. | Implantación |  |  |  |  |  |
|  | Importancia |  |  |  |  |  |
| 4.3 …celebrar sesiones clínicas periódicas con el personal en formación (estudiantes y residentes) para compartir preocupaciones y lecciones aprendidas sobre seguridad del paciente. Esta medida se traduce en la creación de un espacio común para compartir experiencias sobre incidentes de seguridad del paciente, idear barreras para minimizar riesgos y proporcionar apoyo emocional e instrumental entre compañeros. | Implantación |  |  |  |  |  |
|  | Importancia |  |  |  |  |  |
| 4.4 …sensibilizar a los profesionales del centro, con la colaboración de jefes de servicio, sobre la necesidad de alentar a estudiantes, residentes y compañeros a expresar de forma abierta y directa sus preocupaciones relativas a la seguridad de los pacientes y advertir a otros profesionales de los riesgos que identifiquen en su quehacer diario. | Implantación |  |  |  |  |  |
|  | Importancia |  |  |  |  |  |
| 4.5 …sensibilizar a los profesionales del centro, con la colaboración de jefes de servicio, sobre la importancia de responder de manera positiva a las advertencias de otros profesionales en relación con el cumplimiento de normas relevantes para la seguridad de los pacientes y de reforzar la expresión abierta de preocupaciones específicas por parte de estudiantes y residentes. | Implantación |  |  |  |  |  |
|  | Importancia |  |  |  |  |  |
| 4.6 …ofrecer a estudiantes y residentes la oportunidad de participar como observadores en la planificación de conversaciones de revelación de un evento adverso al paciente afectado/a y su familia. | Implantación |  |  |  |  |  |
|  | Importancia |  |  |  |  |  |
| 4.7 …permitir a estudiantes y residentes la oportunidad de estar presentes durante la discusión y el análisis posterior a la ocurrencia de un incidente de seguridad del paciente. | Implantación |  |  |  |  |  |
|  | Importancia |  |  |  |  |  |
| 4.8 … ofrecer a estudiantes y residentes formación específica sobre cómo efectuar la notificación de los incidentes de seguridad del paciente por los medios oportunos. | Implantación |  |  |  |  |  |
|  | Importancia |  |  |  |  |  |
| 4.9 …ofrecer apoyo institucional a los profesionales sanitarios implicados en un evento adverso para contribuir a la creación de un entorno de trabajo más seguro. | Implantación |  |  |  |  |  |
|  | Importancia |  |  |  |  |  |

**5. SI LO DESEA, utilice este espacio para añadir cualquier aspecto que haya sido omitido en el cuestionario o que considere oportuno señalar en relación con el tema tratado.**

|  |
| --- |

**6. PARA FINALIZAR, por favor indique**

País: Croacia / Estonia / Finlandia / Alemania / Israel / Lituania / Portugal / Eslovaquia / España / Serbia / Otro

Edad: _____________

Sexo:  Hombre  Mujer  Otro

Perfil profesional

Medicina

Enfermería

Farmacia

Obstetricia

Fisioterapia

Psicología

Sociología

Otro

Años siendo responsable de estudiantes o residentes: _________

Número de estudiantes/residentes que ha supervisado o tutorizado personalmente en los últimos tres años (2019-2021): _________

¿Existe un programa de formación específico en seguridad del paciente en su centro?  Yes  No

Entorno en el que realiza su trabajo clínico y de tutorización:

Atención primaria

Atención especializada (hospital)

Atención sociosanitaria
