# Supplementary material for: Psychological Safety Competency Training During the Clinical Internship From the Perspective of Health Care Trainee Mentors in 11 Pan-European Countries: Mixed Methods Observational Study
Source: JMIR Med Educ. 2024 Oct 7;10:e64125. doi: 10.2196/64125 (PMC11494257; doi:10.2196/64125)
Supplement: Multimedia Appendix 2 [file mededu_v10i1e64125_app2.docx]

**Multimedia Appendix 2. Open-ended survey used in the second phase of the study.**

**ENGLISH VERSION**

**Competencies in healthcare discipline trainees to promote a climate of psychological safety in clinical settings**

***Second round form***

The first consultation of this study revealed the existence of four areas related to psychological safety that could be improved. These results suggest the need to invest efforts from academic and healthcare institutions, and at a more global level, to foster, among healthcare trainees, competencies that will enable them to respond constructively to the following situations typical of clinical settings:

- Communicating patient safety concerns or initiatives.
- Observing another healthcare professional ignoring an important patient safety rule and assertively warning them about the risks of their behaviour.
- Responding assertively and with self-esteem to the negative reaction of a healthcare professional whom we have warned about the patient safety risks of their behaviour.
- Offering support to a colleague suffering emotionally after being involved in an adverse event.

With this form, we would like to know what **measures, recommendations, or actions you think would be useful to foster these psychological safety competencies** in healthcare trainees and thus contribute to patient safety. Also, in your experience in your work environment, **what things work best for you and can be an example for colleagues in other countries to promote the acquisition of these competencies among their trainees**. Please, when making your contributions, think about what could be done at any level (institutional, political, legal, academic, etc.) to contribute to the acquisition of psychological safety competencies by future healthcare professionals.

**Communication of patient safety concerns or initiatives**

What measures or actions do you propose for healthcare trainees to be able to:

- Recognize one's patient safety concerns about how things are done in the clinical setting.
- Choose words to communicate patient safety concerns honestly.
- Share one's ideas or initiatives for patient safety improvement with any colleagues in the clinical setting without doubt of their validity or fear of being judged.
- Communicate patient safety concerns or initiatives to any colleague in the clinical setting regardless of hierarchical level, gender, age, etc.
- Lose the fear of talking about patient safety.

Use this space to write your proposals:

|  |
| --- |

**Observing another healthcare professional ignoring an important patient safety rule and assertively warning them about the risks of their behaviour**

What measures or actions do you propose for healthcare trainees to be able to:

- Choose the right words to assertively (respecting the rights of the other) and honestly (directly and without hesitation) warn another colleague of the risks of their behaviour in ignoring an important patient safety rule.
- Welcoming (positive attitude) the fact that any professional in the centre can warn another about the risks of not complying with an important patient safety rule.
- Lose fear or dare to warn another colleague of the risks of their behaviour for the patient, regardless of their hierarchical level, sex, age, etc.
- Respond assertively (respecting one's rights and preserving self-esteem) to the negative reaction of a colleague who has been warned of the risks of their behaviour for the patient?

Use this space to write down your proposals:

|  |
| --- |

**Offer support to a colleague suffering emotionally after being involved in an adverse event**

What measures or actions do you propose for healthcare trainees to be able to:

- Not to feel uncomfortable when approached by a peer who expresses discomfort for having been involved in an adverse event.
- Look favourably (positive attitude) on the request and provision of peer support in case of an adverse event.
- Support a colleague who suffers because of their involvement in an adverse event through active listening, without any interventionist intention or intention to investigate what happened.

Use this space to write your proposals:

|  |
| --- |

If you have any additional comments, you can use this last space:

|  |
| --- |

Thank you very much once again for your collaboration.

ERNST Consortium

**CROATIAN VERSION**

**Kompetencije pripravnika u zdravstvenoj djelatnosti za promicanje klime psihološke sigurnosti u kliničkom okruženju**

***Drugi krug istraživanja***

Nakon prvog kruga konzultacija u ovoj studiji, zaključeno je da postoje četiri područja povezana s psihološkom sigurnošću koja bi se mogla poboljšati.

Rezultati sugeriraju potrebu za ulaganjem dodatnog napora u akademskim i zdravstvenim okruženjima, kao i na globalnijoj razini, kako bi se poboljšale kompetencije zdravstvenih pripravnika/specijalizanata u kliničkim okruženjima za sljedeće tipične situacije:

- Komuniciranje o brizi ili inicijativi za sigurnost pacijenata.
- Uočavanje drugog zdravstvenog radnika koji ignorira važno pravilo o sigurnosti pacijenata i asertivno upozoravanje na rizike takvog ponašanja.
- Reagirati asertivno, uz samopoštovanje, na negativnu reakciju zdravstvenog radnika kojeg se upozorilo na ponašanje koje povećava rizik za sigurnost pacijenata.
- Pružanje podrške kolegi koji emocionalno pati nakon što je bio uključen u neželjeni događaj.

S ovim obrascem željeli bismo doznati vase mišljenje **koje mjere, preporuke ili radnje bi bile učinkovite za poticanje navedenih kompetencija psihološke sigurnosti** zdravstvenih pripravnika/specijalizanata te na taj način bi doprinijele sigurnosti pacijenata.

Također, prema vašem radnom iskustvu, **koje preporuke su najučinkovitije i mogu biti primjer kolegama u drugim zemljama za stjecanje ovih kompetencija među svojim polaznicima**.

Molimo vas da prilikom davanja svog doprinosa razmislite o tome što bi se moglo učiniti na bilo kojoj razini (institucionalnoj, političkoj, pravnoj, akademskoj itd.) kako bi se pridonijelo stjecanju kompetencija psihološke sigurnosti budućih zdravstvenih djelatnika.

**Komuniciranje o brizi ili inicijativi za sigurnost pacijenata**

Koje mjere ili radnje predlažete kako bi zdravstveni pripravnici mogli:

- Prepoznati nečiju brigu za sigurnost pacijenata prilikom rada kliničkom okruženju.
- Odabrati riječi kojima će iskreno izraziti zabrinutost za sigurnost pacijenata.
- Podijeliti otvoreno svoje ideje ili inicijative za poboljšanje sigurnosti pacijenata sa svim kolegama u kliničkom okruženju i bez straha od osude.
- Komunicirati o brizi ili inicijativi za sigurnost pacijenata sa svim kolegama u kliničkom okruženju bez obzira na hijerarhijsku razinu, spol, dob itd.
- Izgubiti strah od razgovora o sigurnosti pacijenata.

Koristite ovaj prostor za pisanje svojih prijedloga:

|  |
| --- |

**Uočavanje drugog zdravstvenog radnika koji ignorira važno pravilo o sigurnosti pacijenata i asertivno upozoravanje na rizike takvog ponašanja**

Koje mjere ili radnje predlažete kako bi zdravstveni pripravnici mogli sljedeće:

- Izabrati prave riječi kako bi se asertivno (poštujući prava druge osobe) I iskreno (direktno i bez osuđivanja) upozorilo drugog kolegu na rizično ponašanje u ignoriranju važnog pravila za sigurnost pacijenata
- Pozdraviti činjenicu (pozitivno se odnositi) da se svaki stručnjak može obratiti drugom stručnjaku vezano uz rizik nepoštovanja važnog pravila za sigurnost pacijenata.
- Osloboditi se straha od upozoravanja drugog djelatnika na rizično ponašanje vezano uz sigurnosot pacijenta i to bez obzira na hijerarhiju, spol, dob itd.
- Odgovoriti asertivno (poštujući tuđa prava uz zadržavanje samopoštovanja) na negativnu reakciju kolege koji je upozoren na ponašanje koje povećava rizik za pacijenta?

Koristite ovaj prostor za pisanje svojih prijedloga:

|  |
| --- |

**Ponuditi podršku kolegi koji emocionalno pati nakon što je bio uključen u neželjeni događaj**

Koje mjere ili radnje predlažete kako bi zdravstveni pripravnici mogli imali sposobnost:

- Ne osjećati se neugodno kada mu priđe kolega istovrsnik i izrazi nelagodu zbog sudjelovanja u neželjenom događaju.
- Gledati blagonaklono (pozitivan stav) na zahtjev i/ili pružanje kolegijalne podrške u slučaju neželjenog događaja.
- Podržati kolegu koji pati zbog svoje uključenosti u neželjeni događaj kroz aktivno slušanje, bez ikakve namjere da se intervenira ili namjere da se istraži što se dogodilo.

Koristite ovaj prostor za pisanje svojih prijedloga:

|  |
| --- |

Ako imate dodatnih komentara, molimo napišite:

|  |
| --- |

Najljepše zahvaljujemo na Vašoj suradnji

ERNST Consortium

**ESTONIAN VERSION**

**Psühholoogilise turvalisuse pädevuste omandamine residentuuri ja kliinilise praktika jooksul ning võimalikud sekkumised pädevuste parandamiseks residentuuri ja praktika juhendajate vaatekohast**

***Teine ümmargune vorm***

Uuringu esimese küsitlusvooru tulemused näitasid, et tervishoiuhariduses, tervishoiuorganisatsioonides ja ka globaalsemal tasemel tuleks ette võtta sekkumisi, et parandada residentide ja tervisevaldkonna üliõpilaste psühholoogilise turvalisuse pädevust. Tulemustest selgus, et residentide ja tervisevaldkonna üliõpilaste psühholoogilise turvalisuse pädevust tuleks suurendada neljas psühholoogilise turvalisuse aspektis:

- suhtlemine patsiendiohutusega seotud murede ja algatuste korral;
- patsiendiohutese reeglit rikkuva teise tervishoiutöötaja märkamine ja tema enesekindlalt hoiatamine reeglit rikkuva käitumise riskidest;
- teise tervishoiutöötaja negatiivsele reaktsioonile lugupidav ja enesekindel vastamine pärast seda, kui teist tervishoiutöötajat on hoiatatud tema patsiendiohutuse reegleid rikkuva käitumise riskidest patsiendi tervisele;
- toe ja abi pakkumine kolleegile, kes kannatab emotsionaalselt patsiendile kahju põhjustanud juhtumi tõttu.

Teises küsitlusvoorus soovimegi teada, **mis abinõud või tegevused on Teie arvates** nii tervishoiuorganisatsiooni-, poliitika-, õiguse- kui ka haridusetasandil vajalikud, et **suurendada residentide ja tervisevaldkonna üliõpilaste psüholoogilise turvalisuse pädevust neljas aspektis**, ning seeläbi aidata kaasa patsiendiohutuse tagamisele tervishoius. Lisaks palume, et tooksite oma **töökogemusele tuginedes** välja, mis **Teie arvates aitab kõige paremini nendes neljas aspektis residentide ja tervisevaldkonna üliõpilaste pädevuste omandamist parandada**.

**Suhtlemine patsiendiohutusega seotud murede ja algatuste korral**

Mis abinõusid või tegevusi Te soovitate, et residendid ja tervisevaldkonna üliõpilased oskaksid:

- teada anda patsiendiohutusega seotud muredest, kui nad on tuvastanud riske või ohtlikke tegevusi või teha omapoolseid algatusi patsiendiohutuse parandamiseks oma töökohas;
- valida sõnu, mida kasutada patsiendiohutuse mure ausal väljendamisel;
- jagada ideid või ettepanekuid patsiendiohutuse parandamiseks ükskõik kellega oma kolleegidest, ilma et kahtleks nende sobivuses või et kardaks hukkamõistu;
- rääkida patsiendiohutusega seotud muredest või jagada oma ideid või algatusi patsiendiohutuse parandamiseks ükskõik kellega oma kolleegidest, sõltumata kolleegi ametikohast (hierarhilisest tasandist), soost, vanusest jne;
- rääkima patsiendiohutusest hirmu tundmata.

Kirjutage oma ettepanekud siia:

|  |
| --- |

**Patsiendiohutuse reeglit rikkuva tervishoiutöötaja märkamine ja enesekindlalt hoiatamine tema käitumise riskidest;**

**Teise tervishoiutöötaja negatiivsele reaktsioonile lugupidav ja enesekindel vastamine pärast seda, kui teist tervishoiutöötajat oli hoiatatud tema patsiendiohutuse reegleid rikkuva käitumise riskidest patsiendi tervisele.**

Mis abinõusid või tegevusi te soovitate, et residendid ja tervisevaldkonna üliõpilased oskaksid:

- valida õigeid sõnu, et juhtida enesekindlalt (austades kolleegi õigusi) ja ausalt (sirgjooneliselt ja kõhkluseta) kolleegi tähelepanu tema käitumise riskidele, kui ta eirab olulist patsiendiohutuse reeglit;
- heaks kiita (positiivselt suhtuda) fakti, et iga tervishoiustöötaja võib kolleegi tähelepanu juhtida patsiendiohutuse reeglite eiramise riskidele;
- julgelt ja hirmu tundmata hoiata kolleegi reegleid rikkuva käitumise riskidest patsiendi tervisele, sõltumata kolleegi ametikohast;
- vastata enesekindlalt (lugupidavalt ja teise kolleegi õigusi austades) kolleegi negatiivsele reaktsioonile pärast seda, kui ta on hoiatanud kolleegi tema käitumise riskidest patsiendi tervisele.

Kirjutage oma ettepanekud siia:

|  |
| --- |

**Toe ja abi pakkumine kolleegile, kes kannatab emotsionaalselt (süütunne, alaväärsustunne, ärevus, häbi, ülivalvsus või lein) patsiendile kahju põhjustanud juhtumi tõttu**

Mis abinõusid või tegevusi te soovitate, et residendid ja tervisevaldkonna üliõpilased oskaksid:

- valida õigeid sõnu, et juhtida enesekindlalt (austades kolleegi õigusi) ja ausalt (sirgjooneliselt ja kõhkluseta) kolleegi tähelepanu tema käitumise riskidele, kui ta eirab olulist patsiendiohutuse reeglit;
- heaks kiita (positiivselt suhtuda) fakti, et iga tervishoiustöötaja võib kolleegi tähelepanu juhtida patsiendiohutuse reeglite eiramise riskidele;
- julgelt ja hirmu tundmata hoiata kolleegi reegleid rikkuva käitumise riskidest patsiendi tervisele, sõltumata kolleegi ametikohast;
- vastata enesekindlalt (lugupidavalt ja teise kolleegi õigusi austades) kolleegi negatiivsele reaktsioonile pärast seda, kui ta on hoiatanud kolleegi tema käitumise riskidest patsiendi tervisele.

Kirjutage oma ettepanekud siia:

|  |
| --- |

Kui soovite teema kohta veel midagi lisada, kirjutage siia:

|  |
| --- |

Täname Teid veelkord koostöö eest!

ERNST (The European Researchers' Network Working on Second Victims) konsortsium

**GERMAN VERSION**

**Kompetenzen von Auszubildenden in Gesundheitsberufen zur Förderung eines Klimas der psychologischen Sicherheit im klinischen Umfeld**

***Zweite Runde***

Die erste Befragung im Rahmen dieser Studie ergab, dass es vier verbesserungswürdige Bereiche im Zusammenhang mit der psychologischen Sicherheit gibt. Diese Ergebnisse deuten auf die Notwendigkeit hin, in akademischen und Gesundheitseinrichtungen sowie auf globaler Ebene Anstrengungen zu unternehmen, um bei Auszubildenden im Gesundheitswesen Kompetenzen zu fördern, die sie in die Lage versetzen, konstruktiv auf die folgenden für klinische Einrichtungen typischen Situationen zu reagieren:

- Kommunikation von Bedenken oder Initiativen zur Patientensicherheit.
- Eine andere medizinische Fachkraft dabei beobachten, wie sie eine wichtige Regel der Patientensicherheit ignoriert und sie mit Nachdruck vor den Risiken ihres Verhaltens warnen.
- Durchsetzungsfähig und selbstbewusst auf die negative Reaktion einer medizinischen Fachkraft reagieren, die wir vor den Risiken ihres Verhaltens für die Patientensicherheit gewarnt haben.
- Unterstützung für eine:n Kolleg:in, der oder die nach einem unerwünschten Zwischenfall emotional leidet.

Mit diesem Formular möchten wir wissen, welche Maßnahmen, Empfehlungen oder Aktionen Ihrer Meinung nach sinnvoll wären, um diese psychologischen Sicherheitskompetenzen bei Auszubildenden im Gesundheitswesen zu fördern und damit zur Patientensicherheit beizutragen. Außerdem möchten wir von Ihnen wissen, welche Maßnahmen Ihrer Erfahrung nach in Ihrem Arbeitsumfeld am besten funktionieren und als Beispiel für Kollegen in anderen Ländern dienen können, um den Erwerb dieser Kompetenzen bei ihren Auszubildenden zu fördern. Bitte denken Sie bei Ihren Beiträgen darüber nach, was auf jeder Ebene (institutionell, politisch, rechtlich, akademisch usw.) getan werden könnte, um zum Erwerb von psychologischen Sicherheitskompetenzen durch künftige Fachkräfte im Gesundheitswesen beizutragen.

**Kommunikation von Bedenken zur Patientensicherheit oder Initiativen**

Welche Maßnahmen schlagen Sie für Auszubildende vor, damit sie in der Lage sind, folgende Dinge zu tun:

- Erkennen der eigenen Bedenken hinsichtlich der Patientensicherheit in Bezug auf die Arbeitsweise im klinischen Umfeld.
- Wählen von Worten, um Bedenken bezüglich der Patientensicherheit ehrlich zu kommunizieren.
- Teilen ihrer Ideen oder Initiativen zur Verbesserung der Patientensicherheit mit allen Kolleg:innen im klinischen Umfeld, ohne Zweifel an ihrer Gültigkeit zu haben oder Angst zu haben, beurteilt zu werden.
- Mitteilung von Bedenken oder Initiativen zur Patientensicherheit an alle Kolleg:innen im klinischen Umfeld, unabhängig von Hierarchieebene, Geschlecht, Alter usw.
- Verlieren der Angst, über Patientensicherheit zu sprechen.

Nutzen Sie diesen Platz für Ihre Vorschläge:

|  |
| --- |

**Eine andere medizinische Fachkraft beobachten, wie sie eine wichtige Regel der Patientensicherheit ignoriert und sie mit Nachdruck vor den Risiken ihres Verhaltens warnen**

Welche Maßnahmen schlagen Sie für Auszubildende vor, damit sie in der Lage sind, folgende Dinge zu tun:

- Wählen der richtigen Worte, um eine:n andere:n Kolleg:in selbstbewusst (unter Wahrung der Rechte des anderen) und ehrlich (direkt und ohne zu zögern) vor den Risiken des eigenen Verhaltens zu warnen, wenn eine wichtige Regel zur Patientensicherheit ignoriert wird.
- Begrüßen (positive Einstellung) der Tatsache, dass jede Fachkraft im Zentrum eine andere vor den Risiken der Nichteinhaltung einer wichtigen Regel zur Patientensicherheit warnen kann.
- Verlieren der Angst oder den Mut finden, eine:n andere:n Kolleg:in vor den Risiken des eigenen Verhaltens für den Patienten zu warnen, unabhängig von Hierarchieebene, Geschlecht, Alter usw.
- Selbstbewusste Reaktion (unter Wahrung der eigenen Rechte und des Selbstwertgefühls) auf die negative Reaktion eines oder einer Kolleg:jin, der oder die vor den Risiken der eigenen Verhaltens für den Patienten gewarnt worden ist

Nutzen Sie diesen Platz für Ihre Vorschläge:

|  |
| --- |

**Unterstützung für eine:n Kolleg:in, der nach einem unerwünschten Vorfall emotional leidet**

Welche Maßnahmen schlagen Sie für Auszubildende vor, damit sie in der Lage sind, folgende Dinge zu tun:

- sich nicht unwohl zu fühlen, wenn ein:e Kolleg:in sein Unbehagen darüber äußert, an einem Zwischenfall beteiligt gewesen zu sein.
- Die Azubis sehen die Anforderung und Bereitstellung von Peer-Unterstützung (kollegiale Unterstützung) im Falle eines unerwünschten Ereignisses als positiv an (positive Einstellung).
- Unterstützen eines oder einer Kolleg:in, der unter der Beteiligung an einem unerwünschten Ereignis leidet, durch aktives Zuhören, ohne die Absicht, einzugreifen oder den Vorfall zu untersuchen.

Nutzen Sie diesen Platz für Ihre Vorschläge:

|  |
| --- |

Für weitere Kommentare können Sie diesen Platz nutzen:

|  |
| --- |

Vielen Dank für Ihre Mitarbeit.

ERNST Consortium

**PORTUGUESE VERSION**

**Competências que promovem um clima de segurança psicológica no contexto clínico em estagiários/estudantes da área da Ciência da Saúde**

***Formulário: segunda ronda***

A primeira ronda deste estudo permitiu identificar a existência de quatro áreas relacionadas com a segurança psicológica que podem ser melhoradas. Estes resultados sugerem a necessidade de haver um maior investimento por parte das instituições académicas e de saúde e, a um nível global, no sentido de fomentar competências que permitam que os estagiários/estudantes/internos possam responder de uma forma efetiva às seguintes situações características de contextos clínicos:

- Comunicar preocupações ou iniciativas relacionadas com segurança do doente;
- Observar outro profissional de saúde a ignorar um aspeto importante relacionado com a segurança do doente e assertivamente alertar/avisar acerca dos riscos associados a esse comportamento.
- Responder assertivamente e lidar bem com a situação aquando da reação negativa por parte de outro profissional de saúde, que foi alertado acerca de um comportamento que colocou em risco a segurança do doente.
- Dar apoio a um colega que se encontre emocionalmente vulnerável após o envolvimento na ocorrência de um evento adverso.

Com este formulário pretendemos saber quais são as medidas, recomendações ou ações relacionadas com competências psicológicas que consideraria útil fomentar entre os estagiários/alunos/internos e, deste modo, contribuir para melhorar a segurança do doente.

Para além disso, tendo com conta a sua experiência, gostaríamos de saber quais são as ações que resultam melhor para si e que podem ser um exemplo a ser partilhado com colegas de outras instituições ou países, de forma a promover a aquisição de competências entre os seus estagiários/alunos/internos. Por favor, quando partilhar as suas contribuições, pense no que poderá ser feito nos diferentes níveis (institucional, político, legal, académico, etc) para contribuir para a aquisição de competências por parte dos futuros profissionais de saúde na área da segurança psicológica.

**Comunicação de preocupações ou iniciativas relacionadas com a segurança do doente**

Que medidas ou ações propõe para os estagiários/alunos/internos serem capazes de:

- Reconhecer as preocupações individuais, relacionadas com aspetos de segurança do doente , que estão associadas ao modo de atuação no contexto clínico.
- Escolher como comunicar, de forma frontal e aberta, as preocupações associadas à segurança do doente.
- Partilhar as próprias ideias ou iniciativas de melhoria, no âmbito da segurança do doente, com qualquer colega do mesmo contexto clínico, sem duvidar da sua validade ou sem ter receio de ser julgado.
- Partilhar/comunicar preocupações ou iniciativas relacionadas com a segurança do doente, com qualquer colega do mesmo contexto clínico, independentemente do nível hierárquico, género, idade, anos de experiência, etc.
- Não ter receio de falar sobre assuntos relacionados com a segurança do doente.

Utilize este espaço para escrever as suas propostas:

|  |
| --- |

**Observar outro profissional de saúde a ignorar um aspeto importante relacionado com segurança do doente e assertivamente avisá-lo acerca dos riscos do seu comportamento**

Que medidas ou ações propõe para que os estagiários/alunos/internos possam ser capazes de:

- Escolher as palavras certas para avisar/comunicar com o colega acerca dos riscos do seu comportamento ao ignorar um aspeto importante relacionado com a segurança do doente (respeitando os direitos do outro) e de forma aberta e objetiva (diretamente e sem hesitação).
- Receber (com uma atitude positiva) o facto de qualquer profissional poder alertar acerca dos riscos de não respeitar regras /princípios importantes na área da segurança do doente
- Não ter receio ou permitir-se avisar outro colega acerca dos riscos do seu comportamento para a segurança do doente, independentemente, do seu nível hierárquico, género, idade, anos de experiência, etc.
- Responder assertivamente (de forma educada, respeitando os direitos do outro e preservando o seu bem-estar/autoestima) a uma reação negativa de um colega, após ter sido avisado acerca do risco que o seu comportamento tem para o doente.

Utilize este espaço para escrever as suas propostas:

|  |
| --- |

**Dar apoio a um colega que se encontra a vulnerável emocionalmente, após envolvimento num evento adverso**

Que medidas ou ações propõe para que os estagiários/alunos/internos possam ser capazes de:

- Não se sentirem desconfortáveis quando abordados por um colega que expressa vulnerabilidade emocional por ter estado envolvido num evento adverso.
- Olhar favoravelmente (com uma atitude positiva) quando for solicitado o seu apoio para dar “suporte ao colega” em caso de evento adverso.
- Apoiar um colega que se encontra em sofrimento (por ter estado envolvido num evento adverso) através de uma escuta ativa, sem intenção de investigar ou intervir no que aconteceu.

Utilize este espaço para escrever as suas propostas:

|  |
| --- |

Se tem algum comentário adicional, por favor utilize este espaço para o colocar:

|  |
| --- |

Agradecemos novamente a sua colaboração,

ERNST Consortium

**SERBIAN VERSION**

**Kompetencije studenata medicinskih nauka i stažera za promovisanje psihološke sigurnosti u zdravstvenim ustanovama**

***Forma drugog kola***

Prvi ciklus konsultacija u okviru ove studije ukazao je na postojanje četiri oblasti koje se odnose na psihološku sigurnost a koje bi se mogle poboljšati. Ovi rezultati ukazuju na potrebu angažovanja akademskih i zdravstvenih institucija, na širem nivou, kako bi se unapredile kompetencije koje će studentima medicinskih nauka i stažerima omogućiti da konstruktivno reaguju na sledeće situacije tipične za klinička okruženja:

- Kominikacija u vezi sa situacijama koje izazivaju zabrinutost po pitanju bezbednosti pacijenata ili iniciranje ovakve komunikacije
- Posmatranje drugog zdravstvenog radnika koji ignoriše pravilo važno za bezbednost pacijenata i asertivno upozenje u vezi sa rizicima takvog ponašanja.
- Asertivno reagovanje uključujući samopoštovanje na negativnu reakciju zdravstvenog radnika koga smo upozorili na rizike po bezbednost pacijenata usled njiegovog ponašanja.
- Pružanje podrške kolegi koji emotivno pati nakon što je bio uključen u neželjeni događaj.

Putem ovog istraživanja želimo da znamo koje **mere, preporuke ili aktivnosti bi po vašem mišljenju bile korisne za unapređenje kompetencija psihološke** bezbednosti kod studenata medicinskih nauka i stažera, te kako bismo doprineli bezbednosti pacijenata. Takođe, **značajno nam je da saznamo iz vašeg iskustva u vašem radnom okruženju, šta je ono što vama najviše koristi a može biti primer kolegama u drugim zemljama kako bi se ove kompetencije promovisale**. Molimo vas da, kada dajete svoj doprinos, razmislite o tome šta bi se moglo učiniti na bilo kom nivou (institucionalnom, političkom, pravnom, akademskom, itd.) kako bi se doprinelo sticanju kompetencija psihološke bezbednosti od strane budućih zdravstvenih radnika.

**Saopštavanje zabrinutosti za bezbednost pacijenata ili inicijativa**

Koje mere ili aktivnosti predlažete kako bi studenti medicinskih nauka i stažeri mogli da:

- Prepoznaju zabrinutost za bezbednost pacijenata u vezi sa načinom realizacije određenih aktivnosti u kliničkom okruženju.
- Biraju reči da bi iskreno preneli zabrinutost za bezbednost pacijenata.
- Podele svoje ideje ili inicijative za poboljšanje bezbednosti pacijenata sa kolegama u kliničkom okruženju bez sumnje u njihovu ispravnost ili straha od osude.
- Prenesu zabrinutost za bezbednost pacijenata ili inicijativu svakom kolegi u kliničkom okruženju bez obzira na hijerarhijski nivo, pol, godine itd.
- Nemaju strah od razgovora o bezbednosti pacijenata.

Koristite ovaj prostor da napišete svoje predloge:

|  |
| --- |

**Posmatranje drugog zdravstvenog radnika koji ignoriše važno pravilo o bezbednosti pacijenata i asertivno ih upozorava na rizike od njihovog ponašanja**

Koje mere ili aktivnosti predlažete da bi studenti medicinskih nauka i stažeri mogli da:

- Izaberu prave reči da asertivno (poštujući prava drugih) i iskreno (direktno i bez oklevanja) upozore drugog kolegu na rizike od njihovog ponašanja u ignorisanju važnog pravila bezbednosti pacijenata.
- Podrže (uz pozitivan stav) činjenicu da svaki zdravstveni profesionalac može upozoriti drugog na rizike nepoštovanja važnog pravila o bezbednosti pacijenata.
- Bez oklevanja upozore drugog kolegu na rizike njihovog ponašanja za pacijenta, bez obzira na njihov hijerarhijski nivo, pol, godine itd.
- Reaguju asertivno (samopouzdano i poštujući svoja prava) na negativnu reakciju kolege koji je upozoren na rizike njihovog ponašanja za pacijenta?

Koristite ovaj prostor da zapišete svoje predloge:

|  |
| --- |

**Pružanje podrške kolegi koji emotivno pati nakon što je bio uključen u neželjeni događaj**

Koje mere ili aktivnosti predlažete da bi studenti medicinskih nauka i stažeri mogli da:

- Ne osećaju neprijatnost kada mu se obrati kolega koji izražava nelagodu zbog toga što je bio umešan u neželjeni događaj.
- Budu blagonakloni (uz pozitivan stav) na zahtev i pružanje podrške kolegama u slučaju neželjenih događaja.
- Podrže kolegu koji pati zbog svog učešća u neželjenom događaju kroz aktivno slušanje, bez ikakve namere da proverava šta se dogodilo i da li su ispoštovana pravila.

Koristite ovaj prostor da napišete svoje predloge:

|  |
| --- |

Ukoliko imate dodatne komentare, možete koristiti ovaj poslednji prostor:

|  |
| --- |

Hvala vam još jednom na saradnji.

ERNST Consortium

**SLOVAK VERSION**

**Kompetencie stážistov v oblasti zdravotníckeho vzdelávania na podporu atmosféry psychologickej bezpečnosti v klinickom prostredí**

***Formulár***

Prvé kolo tejto štúdie odhalilo existenciu štyroch oblastí súvisiacich s psychologickou bezpečnosťou, ktoré by sa mohli zlepšiť. Tieto výsledky naznačujú, že je potrebné investovať úsilie akademických a zdravotníckych zariadení a tiež na globálnej úrovni na podporu kompetencií stážistov v zdravotníctve, ktoré by im umožnili konštruktívne reagovať na nasledovné situácie typické pre klinické prostredie:

- Komunikovať obavy týkajúce sa bezpečnosti pacientov alebo dávať podnety na zvýšenie bezpečnosti pacientov.
- Asertívne varovať zdravotníckeho pracovníka pred rizikami, ktoré môže jeho správanie priniesť, ak ho pozoruje, že ignoruje dôležité pravidlo bezpečnosti pacientov.
- Asertívne a so sebaúctou reagovať na negatívnu reakciu zdravotníckeho pracovníka, ktorého varovali pred rizikami ich správania pre bezpečnosť pacientov.
- Ponúknuť podporu kolegovi, ktorý emocionálne trpí po tom, čo bol zapojený do nežiaducej udalosti.

Prostredníctvom tohto formulára by sme chceli zistiť, aké opatrenia, **odporúčania alebo kroky by podľa Vás boli užitočné na podporu týchto kompetencií v oblasti psychologickej** **bezpečnosti**  u stážistov v zdravotníctve, a ktoré by tým prispeli k bezpečnosti pacientov. Tiež chceme zistiť, na základe Vašich skúseností vo Vašom pracovnom prostredí, **čo funguje najlepšie pre podporu týchto zručností u stážistov a môže to byť príkladom pre kolegov v iných krajinách.** Prosím, keď budete vypĺňať formulár, premýšľajte o tom, čo by sa dalo urobiť na ktorejkoľvek úrovni (inštitucionálnej, politickej, právnej, akademickej, atď.) aby sa prispelo k získaniu kompetencií v oblasti psychologickej bezpečnosti u budúcich zdravotníkov/čok.

**Komunikovanie obáv týkajúcich sa bezpečnosti pacientov alebo dávanie podnetov na zvýšenie bezpečnosti pacientov.**

Aké opatrenia alebo kroky navrhujete pre stážistov v zdravotníctve, aby boli schopní:

- rozpoznať svoje obavy o bezpečnosť pacientov, ktoré sa týkajú toho ako sa veci robia v klinickom prostredí.
- vyberať slová, ktorými úprimne vyjadria obavy o bezpečnosť pacientov.
- podeliť sa o svoje nápady alebo podnety na zlepšenie bezpečnosti pacientov s ktorýmkoľvek kolegom v klinickom prostredí bez pochybností o ich platnosti alebo strachu z posudzovania.
- komunikovať obavy alebo podnety týkajúce sa bezpečnosti pacientov ktorémukoľvek kolegovi v klinickom prostredí bez ohľadu na hierarchickú úroveň, pohlavie, vek atď.
- zbaviť sa strachu z rozprávania o bezpečnosti pacientov.

Využite tento priestor na napísanie svojich návrhov:

|  |
| --- |

**Asertívne varovanie zdravotníckeho pracovníka pred rizikami, ktoré môže jeho správanie priniesť, ak je pozorovaný, že ignoruje dôležité pravidlo bezpečnosti pacientov.**

Aké opatrenia alebo kroky navrhujete pre stážistov v zdravotníctve, aby boli schopní:

- vybrať správne slová, aby asertívne (rešpektujúc práva druhého) a úprimne (priamo a bez váhania) varovali iného kolegu pred rizikami jeho/jej správania ak ignoruje dôležité pravidla bezpečnosti pacienta.
- Pristupovať pozitívne k skutočnosti, že každý odborník v zdravotníckom zariadení môže varovať ostatných pred rizikami nedodržania dôležitého pravidla bezpečnosti pacienta.
- zbaviť sa strachu alebo sa odvážiť varovať iného kolegu pred rizikami jeho/jej správania pre pacienta bez ohľadu na jeho hierarchickú úroveň, pohlavie, vek atď.
- reagovať asertívne (rešpektujúc svoje práva a zachovávať si sebaúctu) na negatívnu reakciu kolegu/kolegyne, ktorá bola upozornená na riziká jej správania pre pacienta.

Využite tento priestor na zapisovanie svojich návrhov:

|  |
| --- |

**Ponúkanie podpory kolegovi/kolegyni, ktorý/á emocionálne trpí po tom, čo bol zapojený/á do nežiaducej udalosti.**

Aké opatrenia alebo kroky navrhujete pre stážistov v zdravotníctve, aby boli schopní:

- necítiť sa nepríjemne, keď sa na nich obráti kolega, ktorý vyjadruje diskomfort po tom, čo bol zapojený do nežiaducej udalosti.
- pristupovať pozitívne k žiadosti kolegu/kolegyne a poskytnúť podporu v prípade nežiaducej udalosti.
- podporiť kolegu, ktorý trpí po tom, čo bol zapojený do nežiaducej udalosti prostredníctvom aktívneho počúvania, bez úmyslu zasahovať alebo úmyslu vyšetrovať, čo sa stalo.

Využite tento priestor na napísanie svojich návrhov:

|  |
| --- |

Ak máte ďalšie komentáre, môžete ich uviesť tu:

|  |
| --- |

Ešte raz vám veľmi pekne ďakujeme za spoluprácu.

Konzorcium ERNST

**SPANISH VERSION**

**Competencias en residentes y estudiantes de disciplinas sanitarias para promover un clima de seguridad psicológica en contextos clínicos**

***Formulario segunda ronda***

La primera consulta de este estudio puso de manifiesto la existencia de cuatro áreas relacionadas con la seguridad psicológica susceptibles de mejora. Estos resultados sugieren la necesidad de invertir esfuerzos desde las instituciones académicas y sanitarias, y también a un nivel más global, para fomentar entre los residentes y estudiantes competencias que les permitan responder de forma constructiva a las siguientes situaciones propias de los entornos clínicos:

- Comunicación de preocupaciones o iniciativas sobre seguridad del paciente.
- Observar a otro profesional sanitario que está ignorando una norma de seguridad del paciente importante y advertirle de forma asertiva sobre los riesgos de su conducta.
- Responder de forma asertiva y con autoestima a la reacción negativa de un profesional sanitario al que hemos advertido de los riesgos de su conducta para la seguridad de los pacientes.
- Ofrecer apoyo a un compañero que está sufriendo emocionalmente tras verse involucrado en un evento adverso.

Con este formulario, nos gustaría conocer qué **medidas, recomendaciones o acciones crees que sería útil adoptar para fomentar estas competencias de seguridad psicológica** en los residentes y estudiantes de disciplinas sanitarias y contribuir así a la seguridad de los pacientes. También, en tu experiencia en tu entorno de trabajo, qué cosas os funcionan mejor y pueden ser un ejemplo para que colegas de otros países fomenten la adquisición de estas competencias entre sus residentes y estudiantes. Por favor, a la hora de realizar tus aportaciones piensa en aquello que se podría hacer a cualquier nivel (institucional, político, legal, académico, etc.) para contribuir a la adquisición de competencias en seguridad psicológica por parte de los futuros profesionales sanitarios.

**Comunicación de preocupaciones o iniciativas sobre seguridad del paciente**

Qué medidas o acciones propones para que los residentes y estudiantes de disciplinas sanitarias sean capaces de…

- Reconocer las propias preocupaciones sobre la seguridad de los pacientes en relación con en el modo en que se hacen las cosas en el entorno clínico.
- Escoger las palabras para comunicar de forma honesta las preocupaciones sobre seguridad del paciente.
- Compartir las propias ideas o iniciativas para la mejora de seguridad del paciente con cualquier compañero del entorno clínico sin dudar de su validez ni temor a ser juzgados.
- Comunicar las preocupaciones o iniciativas de seguridad del paciente a cualquier compañero del entorno clínico con independencia de su nivel jerárquico, sexo, edad, etc.
- Perder el miedo a hablar sobre la seguridad del paciente.

Utiliza este espacio para escribir tus propuestas:

|  |
| --- |

**Observar a otro profesional sanitario que está ignorando una norma de seguridad del paciente importante y advertirle de forma asertiva sobre los riesgos de su conducta**

Qué medidas o acciones propones para que los residentes y estudiantes de disciplinas sanitarias sean capaces de…

Escoger las palabras adecuadas para advertir de manera asertiva (respetando los derechos del otro) y honesta (directa y sin titubeos) a otro compañero de los riesgos de su conducta al ignorar una norma importante de seguridad del paciente.

- Ver con buenos ojos (actitud positiva) que cualquier profesional del centro pueda advertir a otro sobre los riesgos de incumplir una norma importante de seguridad del paciente.
- Perder el miedo o atreverse a advertir a otro compañero de los riesgos de su conducta para al paciente con independencia de su nivel jerárquico, sexo, edad, etc.
- Responder de forma asertiva (respetando los propios derechos y preservando la autoestima) ante la reacción negativa de un compañero al que se le ha advertido de los riesgos de su conducta para el paciente.

Utiliza este espacio para escribir tus propuestas:

|  |
| --- |

**Ofrecer apoyo a un compañero que está sufriendo emocionalmente tras verse involucrado en un evento adverso**

Qué medidas o acciones propones para que los residentes y estudiantes de disciplinas sanitarias sean capaces de…

- No sentirse incómodos al ser interpelados por un compañero que expresa su malestar por haberse visto implicado en un evento adverso.
- Ver con buenos ojos (actitud positiva) la solicitud y provisión de apoyo entre iguales en caso de evento adverso.
- Apoyar a un compañero que sufre por su implicación en un evento adverso a través de la escucha activa, sin intención intervencionista ni de investigar lo ocurrido.

Utiliza este espacio para escribir tus propuestas:

|  |
| --- |

Si quieres aportar algún comentario adicional puedes utilizar este último espacio.

|  |
| --- |

Muchas gracias por tu colaboración.

ERNST Consortium
