# Supplementary material for: Psychological Safety Competency Training During the Clinical Internship From the Perspective of Health Care Trainee Mentors in 11 Pan-European Countries: Mixed Methods Observational Study
Source: JMIR Med Educ. 2024 Oct 7;10:e64125. doi: 10.2196/64125 (PMC11494257; doi:10.2196/64125)
Supplement: Multimedia Appendix 3 [file mededu_v10i1e64125_app3.docx]

**Multimedia Appendix 3. Descriptive analysis (mean, SD, and coefficient of variability) of the degree of acquisition or implementation and significance of psychological safety competencies and institutional interventions (consensus conference).**

**Table S1. Degree of acquisition and significance of psychological safety competencies (knowledge component) in the opinion of mentors (n = 173).^a^**

| **In my opinion, internships in my work environment provide trainees with the competency to…** | **Acquisition** | | **Significance** | |
| --- | --- | --- | --- | --- |
|  | **M (SD)** | **CV** | **M (SD)** | **CV** |
| 1.1. …understand that an open and direct expression of concerns about patient safety can prevent the occurrence of incidents that could cause harm to the patient. | 3.8 (1.0) | 25.0 | 4.8 (0.6) | 25.0 |
| 1.2. …know how to communicate assertively a concern about patient safety to another healthcare professional (of the same level or higher) (what words to choose, how to start and finish the conversation, what tone of voice or gestures to use, etc.). | 3.3 (1.0) | 30.0 | 4.6 (0.8) | 30.0 |
| 1.3. …distinguish between situations that could cause avoidable harm to the patient from those that do not represent a high risk for the patient safety. | 3.7 (0.8) | 22.3 | 4.6 (0.7) | 22.3 |
| 1.4. …choose the best moment to communicate specific concerns about patient safety to another healthcare professional (of the same level or higher). | 3.5 (1.0) | 29.3 | 4.4 (0.9) | 29.3 |
| 1.5. …know how to assertively warn another healthcare professional (of the same level or higher) of the risk of ignoring an important patient safety rule (words to choose, how to start and finish the conversation, what tone of voice or gestures to use, etc.). | 3.1 (1.0) | 33.1 | 4.5 (0.8) | 33.1 |
| 1.6. …know how to deal constructively with the possible negative reaction of a healthcare professional (of the same level or higher) after having warned them that they were overlooking an important rule for patient safety. | 2.9 (1.1) | 38.6 | 4.5 (0.8) | 38.6 |
| 1.7. …know how to express specific proposals that could improve the patient safety in the unit. | 3.4 (1.1) | 31.4 | 4.5 (0.8) | 31.4 |

^a^Scale 1-5; CV: coefficient of variability.

**Table S2. Degree of acquisition and significance of psychological safety competencies (attitudes component) in the opinion of mentors (n = 173).^a^**

| **In my opinion, internships in my work environment provide trainees with the competency to…** | **Acquisition** | | **Significance** | |
| --- | --- | --- | --- | --- |
|  | **M (SD)** | **CV** | **M (SD)** | **CV** |
| 2.1. …commit to the identification and prevention of risks for patient safety. | 3.8 (1.0) | 25.3 | 4.7 (0.7) | 14.9 |
| 2.2. ...perceive risk situations in daily work as an opportunity to highlight the risk and take appropriate measures to prevent harm to patients. | 3.5 (1.0) | 27.5 | 4.5 (0.8) | 17.8 |
| 2.3. …respond positively to the expression of warnings or concerns that other healthcare professionals (of the same level or higher) make in relation to patient safety. | 3.6 (1.0) | 27.0 | 4.6 (0.8) | 17.0 |
| 2.4. …maintain a positive attitude towards warning other healthcare professionals if with their actions they are ignoring an important patient safety rule. | 3.3 (1.0) | 31.2 | 4.5 (0.8) | 18.0 |
| 2.5. …be willing to openly and directly share specific proposals to improve patient safety. | 3.5 (1.1) | 30.9 | 4.6 (0.7) | 15.3 |
| 2.6. …be willing to learn from mistakes and patient safety incidents in which other professionals have been involved, instead of judging them. | 3.6 (1.1) | 31.4 | 4.8 (0.6) | 13.2 |

^a^Scale 1-5; CV: coefficient of variability.

**Table S3. Degree of acquisition and significance of psychological safety competencies (skills component) in the opinion of mentors (n = 173).^a^**

| **In my opinion, internships in my work environment provide trainees with the competency to…** | **Acquisition** | | **Significance** | |
| --- | --- | --- | --- | --- |
|  | **M (SD)** | **CV** | **M (SD)** | **CV** |
| 3.1. …communicate openly and directly to other professionals (of same level or higher) specific concerns about patient safety by presenting information, asking questions, or expressing opinions. | 3.5 (1.1) | 30.5 | 4.7 (0.7) | 15.0 |
| 3.2. …request the responsible professionals' advice to report, in the appropriate system, the occurrence of a patient safety incident that has been witnessed and make the report (if necessary). | 3.4 (1.2) | 35.5 | 4.6 (0.7) | 15.3 |
| 3.3. …warn assertively to another healthcare professional (of the same level or higher) that, with their actions, they are ignoring an important patient safety rule. | 3.2 (1.1) | 35.6 | 4.6 (0.7) | 15.9 |
| 3.4. …respond assertively to the negative reaction of a healthcare professional (of the same level or higher) whom they have warned of ignoring an important patient safety rule. | 3.1 (1.2) | 37.8 | 4.5 (0.9) | 19.1 |
| 3.5. …verbally support and reinforce the initiative of other healthcare professionals (of the same level or higher) to share their specific concerns about patient safety with the rest of the team. | 3.4 (1.1) | 32.7 | 4.5 (0.8) | 17.9 |
| 3.6. …set and communicate concrete proposals to improve patient safety in the own unit or service. | 3.3 (1.1) | 33.6 | 4.5 (0.8) | 17.2 |
| 3.7. …offer peer support to a colleague involved in an adverse event to reduce some of the second victim syndrome (characterized by feelings of guilt, inadequacy, anxiety, shame, hypervigilance, or grief). | 3.1 (1.3) | 41.5 | 4.6 (0.8) | 18.3 |

^a^Scale 1-5; CV: coefficient of variability.

**Table S4. Degree of implementation and significance of institutional actions aimed at fostering the acquisition of psychological safety competencies by trainees in the opinion of mentors (n = 173).^a^**

| **My healthcare institution…** | **Implementation** | | **Significance** | |
| --- | --- | --- | --- | --- |
|  | **M (SD)** | **CV** | **M (SD)** | **CV** |
| 4.1. …implements a training program for new staff (especially trainees) to foster a positive patient safety culture and a psychological safety climate. | 2.8 (1.4) | 49.6 | 4.6 (0.9) | 19.0 |
| 4.2. …appoints an influential group of people to design an intervention plan to foster a trusting climate among healthcare professionals to ensure patient safety. | 2.5 (1.3) | 52.7 | 4.4 (1.0) | 22.2 |
| 4.3. …holds regular clinical sessions with trainees to share patient safety concerns and lessons learned. This measure translates into the set of shared spaces to exchange experiences on patient safety incidents, devise barriers to minimize risks, and provide emotional and instrumental support among peers. | 2.4 (1.3) | 56.9 | 4.5 (0.9) | 21.3 |
| 4.4. …raises awareness among the center’s professionals, with the collaboration of heads of service, of the need to encourage trainees and colleagues to express their concerns regarding patient safety openly and directly and to warn other professionals of the risks they identify in their daily work. | 2.8 (1.3) | 45.9 | 4.5 (0.9) | 20.7 |
| 4.5. …raises awareness among the center’s professionals, with the collaboration of heads of service, of the importance of responding positively to warnings from other professionals regarding compliance with relevant patient safety rules and reinforcing the open expression of specific patient safety concerns by trainees. | 2.8 (1.3) | 46.1 | 4.4 (0.9) | 21.5 |
| 4.6. …provides trainees with the opportunity to participate as observers during the planning of adverse event disclosure conversations with the affected patient and family. | 2.5 (1.4) | 57.5 | 4.2 (1.1) | 27.3 |
| 4.7. …allows trainees to have the opportunity to be present during the discussion and analysis following a patient safety incident. | 2.8 (1.4) | 50.8 | 4.4 (1.0) | 22.3 |
| 4.8. …provides trainees with specific training on reporting patient safety incidents by appropriate means. | 2.5 (1.3) | 52.4 | 4.3 (1.0) | 23.7 |
| 4.9. …offers institutional support to healthcare professionals involved in an adverse event to contribute to better safety at the workplace and patient safety. | 2.7 (1.3) | 49.8 | 4.6 (0.9) | 20.0 |

^a^Scale 1-5. CV: coefficient of variability.
