# Supplementary material for: Psychological Safety Competency Training During the Clinical Internship From the Perspective of Health Care Trainee Mentors in 11 Pan-European Countries: Mixed Methods Observational Study
Source: JMIR Med Educ. 2024 Oct 7;10:e64125. doi: 10.2196/64125 (PMC11494257; doi:10.2196/64125)
Supplement: Multimedia Appendix 4 [file mededu_v10i1e64125_app4.docx]

**Multimedia Appendix 4. Content analysis. Themes, categories, and examples of meaning units by core questions.**

*Abbreviations:*

*PS: patient safety*

*Spon.: spontaneity*

*Co.: countries*

**Figure 1. Themes and categories of key ideas to promote the acquisition of psychological safety competencies among healthcare trainees.**


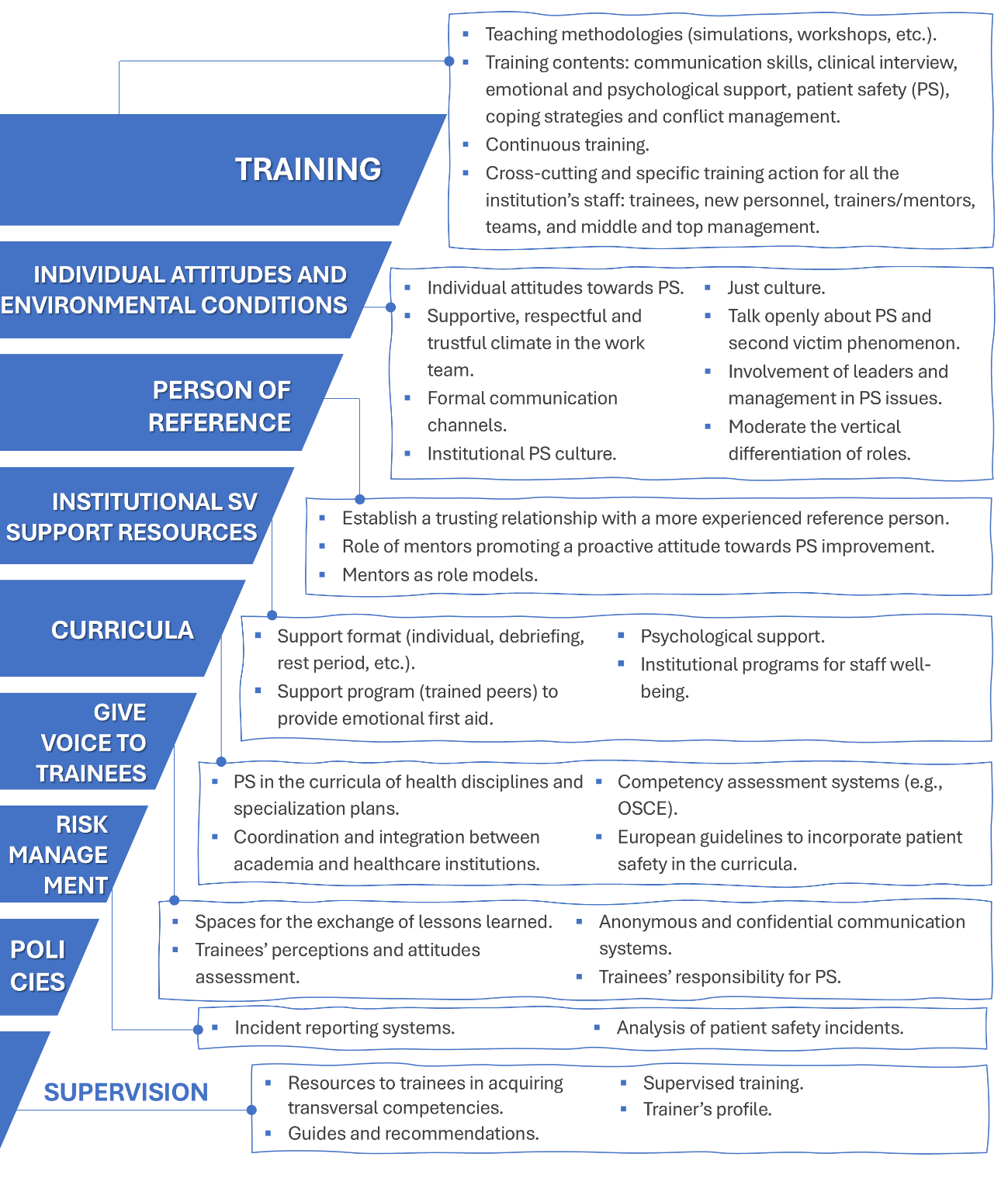


**Table 1. Communication of patient safety concerns or initiatives (Productivity = 154)**

| **Theme 1.1. Training (Spon. = 46)** |
| --- |
| C1.1.1. Teaching methodologies (simulations, workshops, etc.) (Spon. = 13, Co. = 4) |
| *“Communication of students with professional actors during medical studies - creation of a scenario involving different patients, whose safety could be compromised during the provision of health care”.*  *“Invite expert psychologists to meetings who will give advice on how to communicate and provide support”.*  *“Assertive communication workshop”.*  *“To focus on innovative teaching methods that emphasize the student (e.g., application of critical thinking – meaningful learning, moral responsibility)”.*  *“Educational videos with practical demonstrations and model situations”.* |
| C1.1.2. Implementing actions to train staff in communication skills so that they can discuss patient safety concerns and initiatives openly (Spon. = 12, Co. = 6) |
| *“Pre-gradual level: practical training of situation-specific communication skills – e.g., reporting adverse news; communication of concerns/mistakes/suggestions/other opinions, etc. to superiors, etc.”.*  *“Residents and students need to be taught how to communicate, including how to give constructive and negative feedback. Patient safety must be made a priority through training, so that students and residents are accustomed to asking questions of themselves at every point: "is the patient safe because of my actions/inaction?" Then talking about patient safety and expressing concern about it will become routine”.* |
| C1.1.3. Continuous training (including for middle and top management of healthcare institutions) (Spon. = 10, Co. = 4) |
| *“At the institutional level, as part of creating a culture of safety, to introduce regular trainings in communication skills and patient safety skills within lifelong learning, both at the level of management and management of the institution and at the level of executive staff”.*  *“Above all, such workshops should work according to the "top to bottom" principle, i.e., first address those persons in hierarchically higher structures (e.g., chief physicians/senior physicians)”.* |
| C1.1.4. Training for trainers/mentors (Spon. = 6, Co. = 4) |
| *“There is no professional discussion on how to incorporate the area (patient safety) into the education of mentors (clinical practice mentor)”.*  *“Trainees/students/interns should have supervisors/tutors trained in patient safety, as well as in communication and clinical supervision”.* |
| C1.1.5. Supervised training (Spon. = 3, Co. = 2) |
| *“I think that monthly clinical supervision should be introduced, individually and in groups”.*  *“Mentoring programs for interns: in* which each professional can more directly monitor one or more interns”. |
| C1.1.6. Clinical interview training (Spon. = 2, Co. = 2) |
| *“Education of students, interns, and residents about the role of asking open and closed questions at the beginning, during and at the end of the meeting with the patient. Use of standardized questionnaires during medical interviewing - to examine patients' awareness of the risk of medical procedures”.* |
| **Theme 1.2. Patient safety culture (Spon. = 26)** |
| C1.2.1. Foster a climate or environment of trust in the work team (Spon. = 10, Co. = 6) |
| *“All departments should continuously work on improving teamwork, openness and honesty in communication and mutual support”.*  *“Create social programs that allow the informal coexistence of all those involved, promoting the creation or reinforcement of personal and professional relationships of greater trust and empathy”.* |
| C1.2.2. Just safety culture. Systemic approach in dealing with adverse events (Spon. = 8, Co. = 5) |
| *“Promote a culture of learning and non-blame within multidisciplinary teams”.*  *“Psychological safety needs to be talked about more in workplaces/internship centers, where an accusatory-punitive culture currently prevails. Trainees cannot acquire knowledge and attitudes that are not used or are punished for being used. Fear is strongly linked to punishment. Fear does not disappear until the blame of the offender disappears from health care”.* |
| C1.2.3. Modelling behaviours for a positive safety culture and within a climate of open communication (Spon. = 6, Co. = 4) |
| *“Behaviours - Speak openly every day about risk for patient safety and show to the trainees with self-behaviour”.*  *“Their positive influence (persons in hierarchically higher structures -e.g., chief physicians/senior physicians-) can enable an atmosphere for open communication, e.g., in which the chief physicians/senior physicians openly ask the team for their opinion or for suggestions for improvement before the start of an intervention”.* |
| C1.2.4. Tools to promote patient safety (Spon. = 2, Co. = 2) |
| *“Jointly create checklists for certain groups of patients or workplaces where there is a greater safety risk for patients”.*  *“Establishment of a patient safety officer”.* |
| **Theme 1.3. Systems and mechanisms to give trainees a voice (Spon. = 20)** |
| C1.3.1. Create spaces for the exchange of ideas, lessons learned and agreement on best practices (Spon. = 10, Co. = 4) |
| *“Create spaces/moments in the internship fields, which bring together professionals and students, dedicated to reflection and debate on the areas of patient safety and moments of constraint, and the best ways to communicate and act in the prevention of risks and events adverse”.*  *“Encourage clinical sessions or open communication meetings. Systematically implement Patient Safety Briefing-Debriefing meetings”.* |
| C1.3.2. Encourage the assumption of responsibility for patient safety among trainees. To give meaning to their work (Spon. = 6, Co. = 5) |
| *“If we want that the trainees are able to recognize safety aspects and share improvement ideas, we need to have programs that are able to integrate the trainees and can give them some autonomy and independence. If the trainees feel that their daily tasks have a purpose and are integrated in the general teamwork, and that their opinions can be good and valued, they may be more prone to interact and communicate with their team about safety issues”.*  *“If trainees are made to feel that they have no idea about anything, they cannot be expected to muster the courage to raise awareness about possible risks or hazardous situations”.* |
| C1.3.3. Assess trainees' perceptions, concerns, attitudes, and experiences to facilitate the communication and expression of patient safety issues (Spon. = 2, Co. = 1) |
| *“At regular time intervals analyse with trainees their experiences, feelings, concerns”.* |
| C1.3.4. Anonymous and confidential communication systems (Spon. = 1, Co. = 1) |
| *“Establish a mailbox to anonymously deposit negative experiences on the part of the Residents”.* |
| C1.3.5. Other measures to give trainees a voice (Spon. = 1, Co. = 1) |
| *“Create a student diary in which concerns or proposals for ideas and initiatives to improve care safety could be described”.* |
| **Theme 1.4. Curricula (Spon. = 16)** |
| C1.4.1. Incorporate patient safety subjects and content in the curricula of health disciplines (Spon. = 10, Co. = 5) |
| *“Systematic steps to incorporate WHO patient safety curriculum guide: multi-professional edition at national level”.*  *“At the academic level, to introduce compulsory (compulsory optional) subjects: Patient Safety and Communication Enhancing Patient Safety in undergraduate and postgraduate education in all medical study programs”.* |
| C1.4.2. Incorporate patient safety content in specialisation plans and pathways (Spon. = 2, Co. = 2) |
| *“To integrate the topic of patient safety as well as postgraduate specialization training”.*  *“To offer training about safety to the intern”.* |
| C1.4.3. Coordination and integration between academia and healthcare institutions (Spon. = 2, Co. = 2) |
| *“Panel discussions (e.g., on how to share information about risks and errors in clinical practice, gather feedback) between the educational institution and the healthcare facility where the clinical practice is carried out. I would suggest incorporating it into clinical practice mentor training programs”.* |
| C1.4.4. Develop and agree on European guidelines on how to incorporate the subject of patient safety in the curricula of health disciplines (Spon. = 2, Co. = 2) |
| *“There are no guidelines at EU level addressing how patient safety should be taught and regulators have not yet put forward any criteria in this area. WHO guidelines for teaching patient safety are currently underused but could offer a structure and standard to address the shortcomings identified in this work”.*  *“Recommendations and defined competencies for interns of all health professions related to patient safety and communication with patients should be systematically introduced”.* |
| **Theme 1.5. Reference person for trainees (Spon. = 19)** |
| C1.5.1. Promote establishing a trusting relationship with a more experienced reference person (e.g., mentor, colleague, peer, institutional referent, etc.) to discuss questions and concerns and jointly propose appropriate changes in procedures and practices to improve patient safety (Spon. = 10, Co. = 6) |
| *“Have one or more permanent contact persons in whom trainees can confide without being judged or evaluated”.*  *“Create a unit or professional reference position with which the interested party can communicate, and which guarantees confidentiality”.*  *“Recruiting employees who are recognised for this assertive way of communication and proposing as supervisors of behaviours and implementation of this attitude and behaviour”.* |
| C1.5.2. Reinforce the role of mentors and teachers as key actors in promoting a proactive attitude towards improving patient safety and open communication among trainees (Spon. = 9, Co. = 5) |
| *“In my opinion, it would be beneficial if teachers communicated with trainees in an absolutely open way about this issue, led them to believe that patient safety is crucial for their correct diagnosis and treatment, and repeatedly explained to them that they must never put their comfort or safety at the forefront if they have doubts in this regard, but should openly communicate everything with colleagues. or with a supervisor to ensure the safe diagnosis and treatment of their patients”.*  *“Fear of talking about something can only be lost by talking about it and at the same time being encouraged to do so - all employees are required to teach their trainees that the safety and well-being of patients is paramount, and that each person has the right to express any concerns in the respective situations”.* |
| **Theme 1.6. Regulations, guidelines, standards, and policies at the institutional, national and international levels (Spon. = 10, Co. = 4)** |
| *“Each facility should have its own procedure and handbook for patient safety and communication related to patient safety and adverse events and information for healthcare workers on who to contact”.*  *“Legislation should protect the health professional”.*  *“Create a manual with the main principles and requirements for a health organization to be certified as an entity that promotes patient safety in the teaching and training of healthcare students - for example assign a Seal”.* |
| **Theme 1.7. Organisational structure and culture (Spon. = 9)** |
| C1.7.1. Moderate the vertical differentiation of roles in professional interactions and soften hierarchies in interpersonal relationships (Spon. = 6, Co. = 4) |
| *“Hierarchy is a very big problem in health care, because there is a perception of hierarchy and a fear of going against social conventions, which could cost the lower rung, for example, an internship or even a job”.*  *“Create a safe space for communication away from hierarchies”.* |
| C1.7.2. Create formal communication channels to facilitate direct communication between professionals (Spon. = 2, Co. = 2) |
| *“Create direct communication channels in a professional environment between all those involved”.* |
| C1.7.3. Involvement of leaders and management in patient safety issues (Spon. = 1, Co. = 1) |
| *“Leadership engagement in patient safety and that behaves according with patient safety values”.* |
| **Theme 1.8. Patient safety incident reporting systems (Spon. = 8)** |
| C1.8.1. Incident reporting (Spon. = 6, Co. = 3) |
| *“At the system level, to implement an anonymous system focused on reporting adverse events as well as factors that may contribute to adverse events”.*  *“Reporting of complications should be safe - a national system and protection of medical professionals is needed”.* |
| C1.8.2. Incident analysis and adoption of preventive and corrective measures (Spon. = 2, Co. = 2) |
| *“Acquaintance with current adverse events and their consequences, inclusion in their analysis and familiarization with ways to improve patient safety”.*  *“Responsive changes after patient safety incidents reported by students (there is a practical change after reporting)”.* |

**Table 2. Observing another healthcare professional ignoring an important patient safety rule and assertively warning them about the risks of their behaviour (Productivity = 101)**

| **Theme 2.1. Training and capacity building of stakeholders (Spon. = 52)** |
| --- |
| C2.1.1. Teaching methodologies (simulations, workshops, etc.) (Spon. = 24, Co. = 6) |
| *“The mentor organizes exercises in smaller groups of residents/students/trainees, in which they give each other negative feedback in fictional/acted scenarios and reactions are practiced”.*  *“To promote workshops/seminars/practical activities on active listening, communication, emotional intelligence, and conflict resolution”.*  *“Include simulations of clinical cases to discuss patient safety incidents in debriefing sessions”.*  *“The use of videos with simulated or real cases could be an interesting tool for learning ways of communicating in situations such as those described in the statement”.* |
| C2.1.2. Training for trainees (Spon. = 14, Co. = 6) |
| *“Training in assertive communication and interpersonal relations”.*  *“Train non-violent communication”.*  *“Learn to cope with an inadequate response from a colleague who does not yet understand the importance of seeing mistakes to correct them”.* |
| C2.1.3. Training for teams (Spon. = 7, Co. = 3) |
| *“Regular training of the team (including trainees) in the given issue or safety culture”.*  *“Teambuilding”.*  *“Team training in communication and conflict management”.* |
| C2.1.4. Training for new staff and ongoing training (Spon. = 4, Co. = 3) |
| *“To communicate with other colleagues, everyone in the department should undergo introductory training when they arrive (all the same) and then at certain periods of time [...] Education should be structured and continuous (and the same for everyone, regardless of the health profession so that everyone speaks the same language) and education and workshops should be repeated in possible time periods”.* |
| C2.1.5. Involve top and middle management in training their teams in determining appropriate communication channels and styles (Spon. = 2, Co. = 1) |
| *“Approaching site managers in what form they would suggest informing the staff. Every manager has experience of what form of effective communication his employees prefer (in writing, discussion, meeting, presentations...)”.*  *“Regular interviews of the management of individual clinics/departments about the right way of communication”.* |
| C2.1.6. Specialised training for mentors (Spon. = 1, Co. = 1) |
| *“I would suggest incorporating it (panel discussions between the educational institution and the healthcare facility) to clinical practice mentor training programs”.* |
| **Theme 2.2. Individual attitudes, work team and environmental conditioning factors determining behavioural patterns (Spon. = 23)** |
| C2.2.1. Individual attitudes and assertive ability to receive constructive criticism or feedback - Attitudinal awareness and moulding (Spon. = 7, Co. = 3) |
| *“Welcoming (positive attitude) the fact that any professional in the centre can warn another about the risks of not following an important patient safety rule”.*  *“Patient safety is the "be-all and end-all", therefore definitely address justified criticism, one's own ego should always take a back seat”.* |
| C2.2.2. Positive, non-punitive safety culture (systemic approach to adverse events) (Spon. = 7, Co. = 3) |
| *“An environment without primary treatment of adverse events by sanctions, but by subsequent education and prevention”.*  *“To promote a learning environment, with facilitators in inter-team communication”.*  *“If the general culture is not supportive of discussion on the subject and the creation of a blame-free environment, it is difficult to imagine how a resident or student could communicate their input”.* |
| C2.2.3. Organisational structure and culture (Spon. = 5, Co. = 4) |
| *“A non-strict hierarchical work environment (including trainees)”.*  *“The trainees must be accepted and welcomed as a full member of the team, especially by the team leader/supervisor”.* |
| C2.2.4. Leadership and teamwork: supportive and respectful work climate (Spon. = 4, Co. = 4) |
| *“Teamwork in the department and a sense of trust and respect among colleagues of the same health profession, but also of all health professions participating in the team, are extremely important”.*  *“Inclusive and ethical leadership”.* |
| **Theme 2.3. Supervision and support resources for trainees (Spon. = 8)** |
| C2.3.1. Supervised training (Spon. = 5, Co. = 2) |
| *“Mentors regularly ask students/specialists/interns to give them feedback on their behaviour during joint work, in the context of patient safety”.*  *“I think that monthly clinical supervision should be introduced, individually and in groups”.* |
| C2.3.2. Resources to support trainees in acquiring transversal competencies (Spon. = 1, Co. = 1) |
| *“To develop a support system for trainees (accompaniment and support of critical thinking)”.* |
| C2.3.3. Trainer's profile (Spon. = 1, Co. = 1) |
| *“Educators, psychologists with practice not only with theoretical knowledge should participate in teaching”.* |
| C2.3.4. Guides, recommendations (Spon. = 1, Co. = 1) |
| *“Agreed guidelines that can be referred to and followed would be a helpful tool”.* |
| **Theme 2.4. Competency-based curricula and assessment systems (Spon. = 7)** |
| C2.4.1. Incorporate patient safety subjects and content in the curricula of health disciplines (Spon. = 3, Co. = 2) |
| *“To include a subject called "Patient Safety" in non-medical as well as medical curricula”.*  *“Communication is everything: if during the course there are curricular units that address communication strategies towards each other, students will be more prepared to communicate with anyone else”.* |
| C2.4.2. Competency assessment systems (Spon. = 3, Co. = 2) |
| *“To include assessment of communication skills as a mandatory part of the practical part of the final evaluation of clinical subjects and in state and attestation examinations”.*  *“Introduction of Objective Structured Clinical Examination”.* |
| C2.4.3. Formal patient safety training in specialisation plans and pathways (Spon. = 1, Co. = 1) |
| *“Training of communication skills for medical professionals, doctors, also in relation to patient safety, which would be integrated into the teaching of clinical subjects, doctoral studies (as a compulsory subject) and specialization training”.* |
| **Theme 2.5. Institutional management of patient safety risks and adverse events (Spon. = 5)** |
| C2.5.1. Patient safety incident reporting systems (Spon. = 3, Co. = 1) |
| *“Introduce CIRS (critical incident reporting system) into the system”.*  *“An anonymous system for reporting adverse events as well as contributing factors should also be set up at institutional level”.* |
| C2.5.2. Group analysis of critical incidents (Spon. = 1, Co. = 1) |
| *“In the event of a critical incident, jointly and individually conduct a debriefing or seminar on the topic”.* |
| C2.5.3. Clinical sessions (Spon. = 1, Co. = 1) |
| *“Weekly meetings related to patient safety and staff safety topics in the department”.* |
| **Theme 2.6. Reference person for trainees (Spon. = 6)** |
| C2.6.1. Promote the establishment of a trusting relationship with a more experienced reference person (e.g., mentor, colleague) (Spon. = 3, Co. = 3) |
| *“In addition, the mentor makes it known that he is available and that he supports students/specialists/trainees in communication with senior colleagues, bosses, etc.”.*  *“Support from the peers, clinical and pedagogical supervisors”.* |
| C2.6.2. Strengthen the role of mentors as key actors in promoting the acquisition of psychological safety competencies (Spon. = 3, Co. = 3) |
| *“The mentor warns healthcare workers about an observed omission in writing or orally - using professional communication channels, e.g., e-mail or a meeting within a healthcare institution”.*  *“Encourage the trainee to address the person in question about the situation in a timely manner”.* |

**Table 3. Offer support to a colleague suffering emotionally after being involved in an adverse event (Productivity = 98)**

| **Theme 3.1. Training (Spon. = 29)** |
| --- |
| C3.1.1. Communication and support provision training (Spon. = 17, Co. = 6) |
| *“Work on empathy even before starting clinical practice: in this situation, empathy towards the colleagues and team members is necessary, however it is necessary to work on this competence which is not innate for everyone”.*  *“Training in assertive communication and interpersonal relations”.*  *“Health students and residents need to be taught how to support a colleague in crisis”.* |
| C3.1.2. Teaching methodologies (simulations, workshops, etc.) (Spon. = 7, Co. = 5) |
| *“The practice of empathic exercises, which allow you to perceive the moment and return to normality - talking if necessary - as soon as possible”.*  *“In work collectives, situations could be rehearsed where there has been a serious patient incident and where it is necessary to offer help and support to a colleague”.* |
| C3.1.3. Patient safety training (Spon. = 3, Co. = 2) |
| *“To train new employees in an adverse event reporting system so that they have enough information on how they can officially proceed”.*  *“Regular training of the team (including trainees) in the given issue or safety culture”.* |
| C3.1.4. Training in coping and conflict management skills and strategies (Spon. = 2, Co. = 2) |
| *“At professional forums (conferences, seminars) repeatedly inform about coping strategies for stress and stress management”.*  *“Training aimed at resolving internal conflicts”.* |
| **Theme 3.2. Institutional resources to support the second victim (Spon. = 27)** |
| C3.2.1. Support format (Spon. = 9, Co. = 5) |
| *“In the event of a critical incident, jointly and individually conduct a debriefing or seminar on the topic”.*  *“Support the colleague through active listening, without intending to investigate or intervene on the fact that occurred but observing their suffering”.*  *“To suggest a possible period of rest or absence to become aware of the consequences resulting from the event”.*  *“Give space to listen to them and, if necessary, refer to a psychological support consultation”.*  *“Anonymity”.* |
| C3.2.2. Psychological support (Spon. = 8, Co. = 5) |
| *“Organize a psychological support service for health workers, not only interns, at the level of the chambers of health workers, to whom they will be able to turn when they are involved in an unwanted event”.*  *“Although the trainee/student/intern can give some support in these issues, the institutions should have support mechanisms that can help in cases of emotional vulnerability”.* |
| C3.2.3. Support programme or network (trained peers) with training in emotional first aid provision (Spon. = 7, Co. = 3) |
| *“Organizing Balint groups for healthcare workers”.*  *“Designate a reference person who is trained to respond to people who are going through this type of situation. In such a way that anyone knows who to approach, knowing that they will be well received”.*  *“Create multidisciplinary support networks”.* |
| C3.2.4. Institutional programmes and activities for staff well-being (Spon. = 3, Co. = 2) |
| *“Try to create personal development programs that allow achieving an understanding and broad view of the context that surrounds them”.* |
| **Theme 3.3. Individual attitudes and environmental determinants of behavioural patterns (Spon. = 25)** |
| C3.3.1. Climate of support, respect, and trust (Spon. = 10, Co. = 6) |
| *“Respect”.*  *“Friendly behaviour”.*  *“We have to learn not to judge (it can happen to us) and to give support (we work in a team, and this has to be taught)”.* |
| C3.3.2. Talk openly about safety incidents and their impact on professionals (second victim phenomenon) (Spon. = 8, Co. = 6) |
| *“To talk more about errors and importance of the peer support”.*  *“That those responsible for the work centre and superiors help to understand that having a mistake is not being a bad professional and support the results and personal and social consequences”.* |
| C3.3.3. Safety culture (systemic approach to adverse events) (Spon. = 5, Co. = 3) |
| *“An environment without primary treatment of adverse events by sanctions, but by subsequent education and prevention”.*  *“To ensure that there is no punishment for reporting the occurrence of adverse events”.* |
| C3.3.4. Individual attitudes towards work (Spon. = 1, Co. = 1) |
| *“To be independent, responsible for your own work, be confident in your abilities and when you are not sure about it, improve it and work on yourself”.* |
| C3.3.5. Organisational structure and culture (Spon. = 1, Co. = 1) |
| *“A non-strict hierarchical work environment (including trainees)”.* |
| **Theme 3.4. Supervision and a trusted reference person (Spon. = 15)** |
| C3.4.1. Supervision (Spon. = 6, Co. = 4) |
| *“A supervisor position should be legislated in a medical institution”.*  *“Organization of supervision for students/specialists/interns who were exposed to unwanted events”.*  *“Supervision, regular after meetings of special events”.* |
| C3.4.2. Promote establishing of a trusting relationship with a more experienced reference person (e.g., mentor, colleague) (Spon. = 5, Co. = 3) |
| *“[…] willingness to contact a responsible person or a person who is in a position of support and understanding at any time…”.*  *“The mentor regularly checks the emotional state of the students/residents/interns and encourages them to ask for help/support when they need it”.*  *“In which the practice supervisors or area managers before the start of the training offers itself as a neutral person in such cases gladly to support. Possibly, examples could be given in which such a situation was solved by collegial support to encourage the trainees”.* |
| C3.4.3. Mentors as role models (Spon. = 4, Co. = 3) |
| *“The mentor serves as an example - in the conversation with students/residents/interns, he talks about the emotionally difficult experiences he went through and worked through, letting them know that this is normal and should not be hidden”.* |
| **Theme 3.5. Regulations, guidelines, standards, and policies (Spon. = 2, Co. = 2)** |
| *“To proceed in accordance with developed standards”.*  *“In the department/hospital, actively advocate and implement the policy of quality and process improvement to prevent future unwanted events instead of looking for the culprit”.* |
